# Effective Synthesis of 4-Quinolones by Reductive Cyclization of 2′-Nitrochalcones Using Formic Acid as a CO Surrogate

**DOI:** 10.3390/molecules28145424

**Published:** 2023-07-15

**Authors:** Francesco Ferretti, Manar Ahmed Fouad, Cecilia Abbo, Fabio Ragaini

**Affiliations:** 1 Dipartimento di Chimica, Università Degli Studi di Milano, Via C. Golgi 19, 20133 Milano, Italy; francesco.ferretti@unimi.it (F.F.); manar.abdellatif@unimi.it (M.A.F.);; 2 Chemistry Department, Faculty of Science, Alexandria University, P.O. Box 426, Alexandria 21321, Egypt

**Keywords:** quinolones, *N*-heterocycles, palladium, carbonylation, CO surrogate, chalcones, homogeneos catalysis, nitroarenes

## Abstract

4-Quinolones are the structural elements of many pharmaceutically active compounds. Although several approaches are known for their synthesis, the introduction of an aryl ring in position 2 is problematic with most of them. The reductive cyclization of *o*-nitrochalcones by pressurized CO, catalyzed by ruthenium or palladium complexes, has been previously reported to be a viable synthetic strategy for this aim, but the need for pressurized CO lines and autoclaves has prevented its widespread use. In this paper, we describe the use of the formic acid/acetic anhydride mixture as a CO surrogate, which allows us to perform the reaction in a cheap and commercially available thick-walled glass tube without adding any gaseous reagent. The obtained yields are often high and compare favorably with those previously reported by the use of pressurized CO. The procedure was applied to a three-step synthesis from commercially available and cheap reagents of the alkaloid Graveoline.

## 1. Introduction

The 4-quinolone moiety is the key scaffold not only of several antibiotics but also of other compounds with pharmaceutical properties, such as anticancer or anti-HIV activity [1,2,3]. As such, its synthesis continues to be the focus of new or improved synthetic methods. The list of reported synthetic strategies is too long to be discussed here, and we refer to recent reviews as leading guides [4,5]. We wish only to mention a work by Wu in which quinolones are obtained by the reaction of *o*-bromonitroarenes and alkynes because it is the only one, apart from our present work, in which a CO surrogate, Mo(CO)_6_, was employed to reduce the nitroarene [6]. All methods have positive aspects and limitations, but most of them produce stoichiometric amounts of coproducts that may cause problems in the purification step. Taking into account that the nitrogen atom in most aromatic compounds is initially introduced in the form of a nitro group and only at a later stage transformed into other groups, using a nitroarene as a reagent has the intrinsic advantage of saving at least one synthetic step. In this context, reactions employing nitroarenes or nitroalkenes as substrates and carbon monoxide as the reductant are particularly appealing because CO allows the selective reduction of the nitro group in the presence of other reducible groups, such as olefinic and keto ones [7,8], and only produces CO_2_ as an easily separable stoichiometric byproduct [9,10,11,12,13,14,15].

Many years ago, we first reported on the synthesis of 4-quinolones by a reduction of 2’-nitrochalcones by CO, catalyzed by a ruthenium complex with a chelating Schiff base of the Ar-BIAN [16,17] family as a ligand [18] (Ar-BIAN = bis-aryliminoacenaphthene). The method is particularly effective for the synthesis of 2-aryl-4-quinolones, whose production, on the other hand, is problematic with most of the traditional quinolone syntheses [19]. Later work in the same group showed that the use of palladium complexes with phenanthroline ligands can also be employed for this aim, although the reaction was only performed on one specific substrate [20]. In the absence of phenanthroline, the reaction rate and selectivity were low [21]. Note that this is one of the few cyclization reactions of nitroarenes affording a 6-membered ring [10]. The large majority of these reactions afford 5-membered *N*-heterocycles.

Despite the intrinsic advantages of this strategy, the need to employ pressurized CO and autoclaves has strongly limited its use by other groups. This problem is common to other carbonylation reactions, and in recent years several solid or liquid substances have been developed, so-called CO surrogates, which can liberate CO under the reaction conditions. This also allows the reaction to be performed in a thick-walled glass reactor [22,23,24,25,26,27,28,29,30,31,32,33,34]. In our group, we have developed the use of phenyl formate as a CO surrogate for the synthesis of several heterocycles [35,36,37,38,39,40]. During these studies, a single example of cyclization of 2′-nitro-4-methoxychalcone to the corresponding 4-quinolone in a 67% yield was reported, but this specific reaction was not investigated any further [38,39]. Very recently, we were able to also employ the HCOOH/Ac_2_O mixture for this aim, thus eliminating the problem of the formation of phenol as a coproduct, whose complete separation was problematic in some cases [41]. The use of this mixture also improves the atom efficiency of the reaction, since phenyl formate is itself synthesized by the reaction of phenol with the HCOOH/Ac_2_O mixture, although the use of gaseous CO is clearly unbeatable from this point of view.

**Scheme 1:**
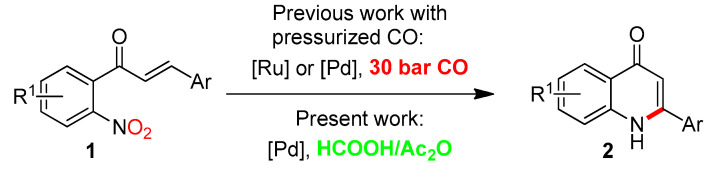
Previous and present work.

The catalyst employed in the present work is a complex of palladium with 1,10-phenanthroline (Phen) as a ligand, which is formed in situ. These complexes were shown to be the most active and robust catalysts for reactions involving a reduction of nitroarenes, not only in the field of cyclization reactions [42,43,44,45,46] but also when the synthesis of base chemicals, such as carbamates and ureas [47,48,49,50,51,52] is involved, where very high turnover numbers are required to make the catalyst economically interesting. By employing this catalytic system together with the HCOOH/Ac_2_O mixture, we were able to convert a series of 2′-nitrochalcones (**1**) into the corresponding 4-quinolones (**2**) in high yields, with acetic acid and CO_2_ as the only stoichiometric byproducts. Moreover, the use of both pressurized CO and an autoclave is avoided, since the reaction can be performed in a thick-walled glass tube, a kind of apparatus that is cheap and commercially available in many sizes (Scheme 1).

## 2. Results and Discussion

### 2.1. Optimization of the Reaction Conditions

As mentioned in the introduction, recently, some of us reported that phenyl formate can be used effectively as a carbon monoxide source in the cyclization of 2′-nitro-4-methoxychalcone to the corresponding 4-quinolone [38,39]. The same unoptimized reaction conditions allowed the isolation of 2-phenylquinolin-4(1*H*)-one (**2a**) in a 78% yield. Despite the satisfactory results, we directed our effort toward the optimization of a catalytic reaction in which the HCOOH/Ac_2_O mixture acts as the CO source for the reasons mentioned in the Introduction.

As a first attempt, the reaction conditions previously optimized for the reductive cyclization of *o*-nitrostyrenes were employed. The result was encouraging though not good (entry 1, Table 1); full conversion was reached but with a low **2a** yield (51%). A higher selectivity was obtained using acetonitrile as the solvent at the same temperature (140 °C) and CO-source to **1a** ratio (4.4) previously employed in the above-mentioned reaction in which HCOOPh had been employed as the CO surrogate. A decrease in the HCOOH/Ac_2_O amount, from 2.2-fold of the stoichiometric amount (i.e., two CO for each nitro group) to 1.5-fold, causes a slight decrease in the reaction rate concurrently with a selectivity increase (entries 2–3). Beneath this amount, the selectivity decreased again (entry 4). Note that the reaction is air sensitive. Assembling the reaction tube in the air led to a much lower yield (entry 5). At variance with what was observed for some related syntheses [53], the use of 3,4,7,8-tetramethylphenathroline (TMPhen) or 4,7-dimethoxyphenanthroline ((MeO)_2_Phen) as the ligands afforded lower selectivities (entries 6–7). A screening of temperatures evidenced that temperatures above 130 °C are needed to ensure high selectivities, with a maximum at 130 °C (entries 8–10). A decrease in the ligand amount led to a slower reaction, albeit a very good selectivity toward **2a** was maintained (entries 11–12). Finally, a variation in the polar non-protic reaction solvent led us to identify the best medium to perform the reaction (entries 12–15). An approximately quantitative HPLC yield was in fact obtained using DMF, which allows the reactions to be completed in 10 h instead of the 16 h required using acetonitrile (entry 16, best conditions). Note that the use of the CH_3_CN/DMF mixture (entry 13) was tested because in a previous work on the reductive cyclization of *o*-nitrostyrenes to indoles [38], this mixed solvent had provided better results than either solvent alone. However, that positive effect is not present in the present system.

### 2.2. Substrate Scope

**Figure 1:**
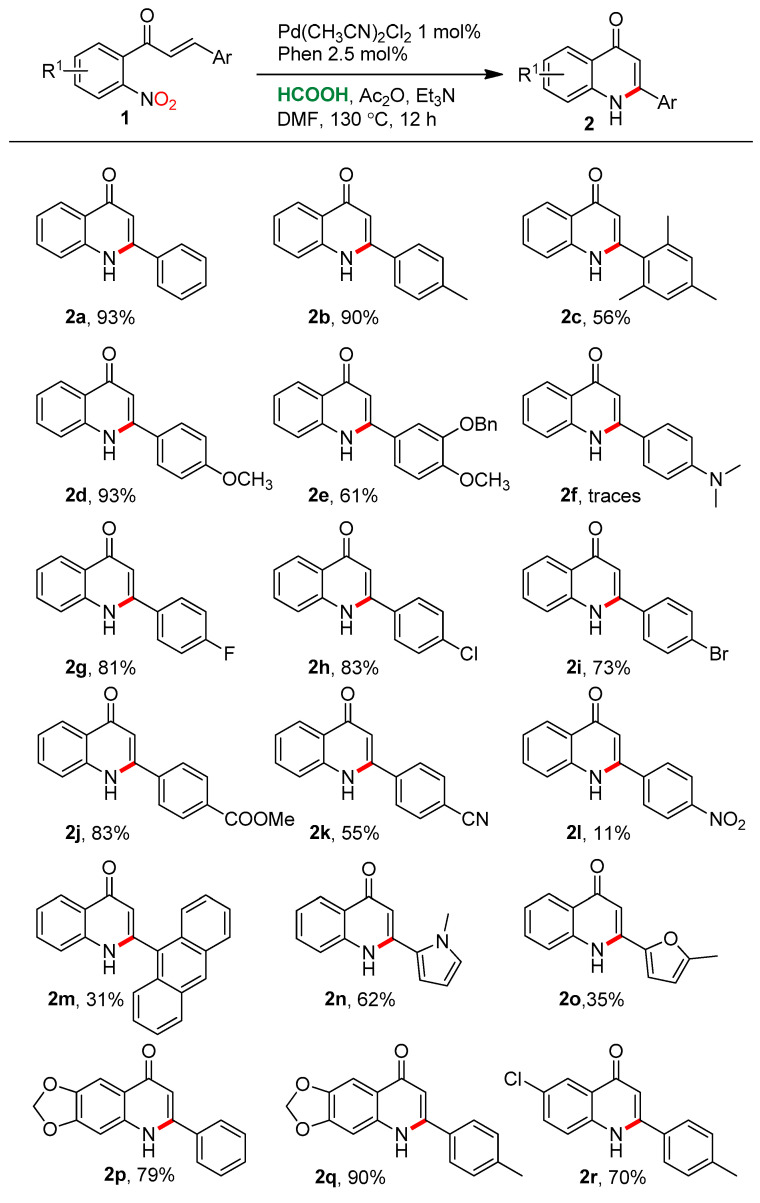
Substrate scope ^1^. ^1^ Experimental conditions: **1** (0.5 mmol), mol ratio FA/Ac_2_O/Et_3_N/**1** = 3, Pd(CH_3_CN)_2_Cl_2_ (1 mol%), ligand (2.5 mol%), DMF (10 mL) at 130 °C for 12 h. Isolated yields are reported.

Having identified the best experimental conditions, the substrate scope was investigated. The reaction time was elongated from 10 to 12 h for these experiments to make sure the reaction would be complete even for less reactive substrates. Results are shown in Figure 1.

Compound **2a** was isolated in a 93% yield. The loss in isolation with respect to the virtually quantitative yield measured by HPLC under the same experimental conditions is largely due to its very small solubility, which results in a longer chromatographic purification. Note that the same problem was encountered even with most of the products shown in Figure 1.

Concerning substituents on the dandling aryl ring, alkyl and alkoxy substituents were well-tolerated (**2b**–**2e**), although a lower yield was observed when they exerted significant steric shielding (**2c**). Surprisingly, when the dimethylamino group was present, only trace amounts of quinolone (**2f**) could be detected by NMR spectroscopy, and significant amounts of 4-dimethylaminobenzaldehyde, derived from a retro-aldol condensation, were formed. High yields were also obtained in the presence of the electron-withdrawing halides (**2g**–**i**) and carbomethoxy (**2j**) groups, although the presence of a cyano substituent (**2k**) gave somewhat worse results. Lower yields are anyway often obtained in this kind of cyclization reaction when a cyano group is present. The yield was low in the presence of a second nitro group (**2l**), but the quinolone could be isolated. For a discussion of this substrate, see the paragraph on the reaction mechanism.

A fused polyaromatic ring can be present, despite the steric hindrance it exerts (**2m**). Most significantly, both the highly oxidizable pyrrole and furan rings (**2n**,**o**) were tolerated. It should be noted that this would not be the case for several of the reported quinolone syntheses, which require the presence of an oxidant.

Finally, both electron-donating (**2p**,**q**) and electron-withdrawing (**2r**) substituents on the aryl ring bearing the nitro group can be present.

In general, yields compare very favorably with those previously obtained by the use of Ru_3_(CO)_12_/Ar-BIAN [18] or by the use of a palladium salt without any ligand [21]. The yield of the only compound (**2s**, see next paragraph) previously obtained by the use of a palladium/phenanthroline catalytic system under CO pressure (30 bar) and with more catalyst (2.5 mol%) is coincidentally the same. However, in the present case, there is no need to employ a pressurized CO line and an autoclave. It should be noted that in the case of the ruthenium catalyst, the main byproduct was the corresponding dihydroquinolone, derived from the Michael addition of an intermediately formed aniline to the conjugated C=C double bond. Indeed, the Ru_3_(CO)_12_/Ar-BIAN couple is known to be an excellent catalytic system for the reduction of nitroarenes to anilines by CO/H_2_O [7], and small amounts of water are unavoidably present as an impurity in carbon monoxide. Palladium catalysts are less prone to this secondary reaction.

**Scheme 2:**
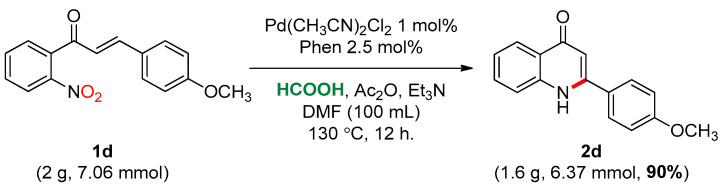
Large-scale synthesis of **2d**.

In order to fully exploit the potentialities of our synthetic protocol, a large-scale synthesis of **2d** was also performed (Scheme 2).

Gratifyingly, the product could be isolated in a 90% yield by simple evaporation of the solvent and crystallization, with only a minor decrease in isolated yield with respect to the smaller scale synthesis (93%).

### 2.3. Synthesis of Graveoline

Graveoline is an alkaloid isolable from *Ruta graveolens* L. but the purification procedure is complex, and only little amounts of pure compound can be obtained [54,55]. Graveoline has been shown to display different pharmacological properties [56]; it is also an apoptosis and autophagy inducer in skin melanoma cancer cells [57]. Moreover, it can even be employed as a platform molecule for the synthesis of more elaborate compounds [58].

**Scheme 3:**
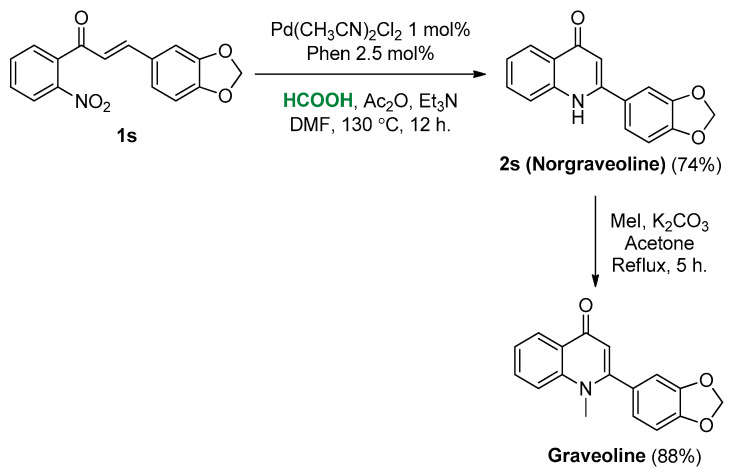
Synthesis of Graveoline.

Our synthetic approach could be successfully applied to the synthesis of Graveoline through methylation of the initially formed Norgraveoline (**2s**). Both the cyclization and the methylation steps occur in high yields (Scheme 3).

### 2.4. Reaction Mechanism

**Scheme 4:**
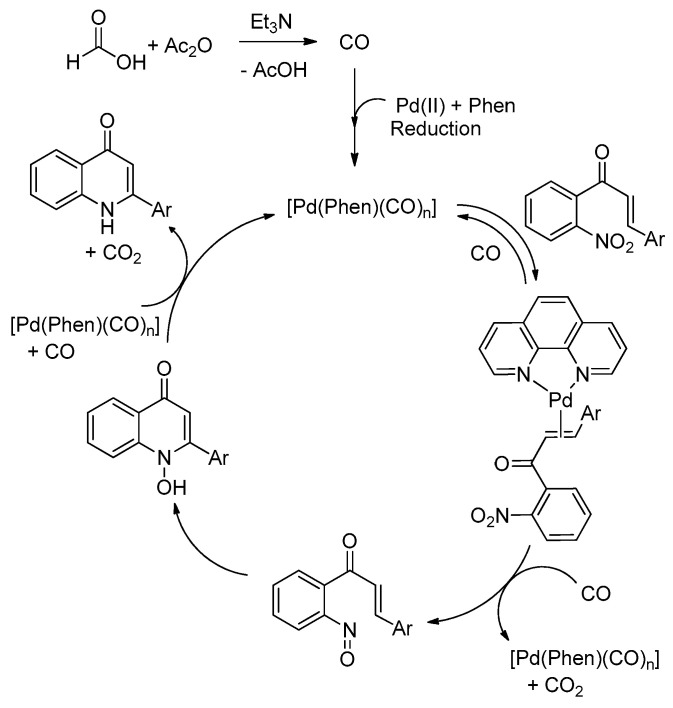
Proposed reaction mechanism.

Although it was not the aim of this work to investigate the reaction mechanism, some information comes from a few observations and previous data, which allow us to propose the reaction scheme shown in Scheme 4.

It is well-assessed that the activation of nitroarenes by transition metal complexes occurs by an electron transfer from the metal complex to the nitroarene, and this step requires the metal to be in a low oxidation state [59,60,61,62,63,64,65,66]. Because of the air sensitivity of zerovalent palladium complexes, palladium(II) precatalysts are typically employed, but their reduction in the presence of carbon monoxide is fast. The identity of the obtained zerovalent complex may depend on the reagents of the reaction. In the case of the reductive cyclization of *o*-nitrostyrenes to indoles, we proposed that in the formed complex the C=C double bond of the nitrostyrene coordinates to palladium. The rationale for this proposal was that the reaction of 2,4-dinitrostilbene afforded 2-phenyl-6-nitroindole in high yield despite the fact that the nitro group in the *para* position should be more easily accessible than that in the *ortho* one. Coordination of the C=C double bond would place the latter in a closer position to the metal and justify the observed selectivity. In the present work, an analogous situation occurs in substrate **1l**, where the second and more accessible nitro group is on the dandling aryl ring. The observed selectivity in nitroquinolone is not as high as in the case of the indole synthesis, but still, the product was obtained, suggesting that even in this case, the C=C double bond is coordinated to the metal. The lower selectivity observed may be due to the fact that the carbonyl group in **1l** increases the distance between palladium and the nitro group in the complex with respect to what occurs with *o*-nitrostyrenes, decreasing the tendency for the *ortho* nitro group to react.

Deoxygenation of the nitro group yields a nitrosoarene. Such kind of compound is known to react quickly with olefinic groups present in the same molecule and the previously reported isolation of *N*-hydroxy-quinolones when a palladium catalyst is employed in the absence of any ligand [21] strongly supports the idea that cyclization occurs at this stage.

As an alternative, one may consider that the reduction in the nitro group proceeds up to the stage of the amino group. The so-formed aminochalcone would afford a dihydroquinolone by a Michael addition and the latter would be oxidized to quinolone by a second molecule of nitrochalcone. However, we have already tested this possibility several years ago by running a competition experiment in which a 2’-nitrochalcone and a 2’-aminochalcone bearing different substituents on the aryl ring were reacted at the same time in the presence of a palladium catalyst and under pressurized CO. Only the quinolone derived from the nitrochalcone was observed at the end of the reaction, with the aminochalcone being recovered unreacted [67]. Although the experimental conditions of that experiment were different from those employed in the present study, it is unlikely that a completely different mechanism is operating in the two cases, and the intermediate formation of anilines can be discarded.

In the final reaction stage, the *N*-hydroxyquinolone is deoxygenated by CO. Such a reaction has been shown to be catalyzed by the same complexes able to reduce nitroarenes in several cases [10,68] and should proceed easily in the present system.

It is also worth noting that despite the fact that imido complexes are still often proposed as intermediates in the cyclization reactions of nitroarenes, no sound experimental evidence has ever been gained for their involvement, and that claimed as such is questionable [10]. We thus did not consider this possibility here.

## 3. Materials and Methods

### 3.1. General Procedures

Unless otherwise specified, all reactions and manipulations were performed under a dinitrogen atmosphere using a standard Schlenk apparatus. All glassware and magnetic stirring bars were kept in an oven at 120 °C for at least two hours and allowed to cool under vacuum before use. Et_3_N and CH_3_CN were dried by distillation from CaH_2_. DMF was dried by distillation over CaH_2_ under reduced pressure at 60 °C. Acetone was degassed and kept over molecular sieves. Dried solvents were stored under a dinitrogen atmosphere. Deuterated solvents were purchased from Sigma-Aldrich (St. Louis, MO, USA) or Eurisotop (Saint-Aubin, France). 1,10-Phenanthroline was purchased as a hydrate (TCI Europe NV, Tokyo, Japan). It was dissolved in CH_2_Cl_2_, dried over Na_2_SO_4_, followed by filtration under a dinitrogen atmosphere and evaporation of the solvent in vacuo. Phen was weighed in the air but stored under dinitrogen to avoid water uptake. The other phenanthrolines were purchased as anhydrous. Pd(CH_3_CN)_2_Cl_2_ was prepared starting from commercially available PdCl_2_ following a procedure reported in the literature [69]. All other reagents were purchased from Merck (Darmstadt, Germany) (Sigma-Aldrich), TCI Europe NV, or Fluorochem (Glossop, UK) and used without further purification. The syntheses of the starting 2′-nitrochalcones are described in the Supplementary Materials. ^1^H NMR and ^13^C NMR spectra were recorded on a Bruker (Billerica, MA, USA) Avance DRX 400 or Avance NEO 400. Chemical shifts are reported in ppm relative to TMS. HPLC analyses were performed using an HP 1050 chromatograph equipped with a LiChroCART 125-4 Purospher RP-18 (Merck, Darmstadt, Germany) end-capped (5 µm) column using isocratic elution (MeOH/H_2_O 6:4 + 0.1% formic acid). For a standard analysis, benzophenone was added to the reaction mixture in the pressure tube, after the reaction, and MeOH (5 mL) was added to ensure complete solubilization. After sonicating the mixture for 5 min, the opportune amount of the sample was taken and added to a 10 mL measuring flask containing the eluent used for the analysis (conc. 0.02 mg/mL calculated with respect to benzophenone used as the internal standard). Elemental analyses were performed on a Perkin Elmer (Shelton, CT, USA) 2400 CHN elemental analyzer.

### 3.2. Catalytic Reactions

Catalytic reactions were performed in 23 mL Duran heavy wall (2.5 mm) borosilicate glass tubes with Schott PTB screw caps completed with PTFE-protected seals placed in a custom-made aluminum block positioned on a magnetic stirring and heating plate (see [38] for details). CAUTION: Although we have never had accidents in our laboratories with over several hundred reactions, the tubes must always be checked for scratches or damages that may result in reduced resistance to pressure. The possibility that an explosion occurs should always be considered, thus the reactions must be performed in a well-ventilated hood and with the use of a protective shield.

To avoid weighing very small amounts of substance, stock solutions of the Pd catalyst and Phen were separately prepared under dinitrogen in the appropriate solvent. For a typical catalytic reaction, a pressure tube equipped with a magnetic stirring bar was charged with 2′-nitrochalcone (0.5 mmol). The tube was placed in a large-mouth Schlenk tube and evacuated and filled with dinitrogen three times. The appropriate volumes of the stock solutions of the catalysts and Phen were added, and the mixture was stirred for 10 min to allow the formation of the Pd/Phen complex. Subsequently, triethylamine (1.5 mmol in the optimized conditions) and acetic anhydride (1.5 mmol in the optimized conditions) were added with stirring by means of a micropipette, and then the remaining amount of solvent (10 mL total volume) was layered without stirring. Finally, formic acid (1.5 mmol in the optimized conditions) was added, and the pressure tube was sealed under dinitrogen. It is worth underlying that the order of addition of the reagents and solvent layering is critical to avoid loss of the formed carbon monoxide. Indeed, as soon as HCOOH, Ac_2_O, and the base are mixed, carbon monoxide started to evolve, even at room temperature. The pressure tube was then placed and heated while stirring in an aluminum block preheated at 130 °C. At the end of the reaction, the pressure tube was removed from the aluminum block, let to cool to room temperature, and slowly opened under a fume hood. For the optimization study, the internal standard was added, and the reaction mixture was analyzed by HPLC. For the substrate scope study, the reaction mixture was transferred to a one-neck round bottom flask in the air, the possibly formed solid was taken up from the pressure tube with CH_2_Cl_2_, and the volatiles were evaporated under reduced pressure at ca. 50 °C. The crude was subjected to silica-gel column chromatography using gradient elution from CH_2_Cl_2_ to CH_2_Cl_2_ + 4% MeOH, unless otherwise stated.

### 3.3. Procedure for Gram-Scale Reaction

A large-scale reaction was carried out in a 250 mL heavy-walled glass pressure bottle to prepare **2d** under the optimized conditions. The reaction was scaled up by increasing the substrate amount 14-fold with respect to the standard conditions. The pressure bottle was charged with solid reagents, substrate **1d** (2.00 g, 7.06 mmol), Pd(CH_3_CN)_2_Cl_2_ (18.4 mg, 0.07 mmol, 1 mol%), and phenanthroline (32.0 mg, 0.18 mmol, 2.5 mol%), and then placed in a Schlenk tube with a large mouth. The tube was evacuated and filled three times with dinitrogen. DMF (30 mL), triethylamine (3.0 mL, 21 mmol), and acetic anhydride (2.0 mL, 21 mmol) were added, and the mixture was stirred for 10 min. The stirring was stopped, and the remaining solvent amount (DMF, 70 mL) was layered. At last, formic acid (0.80 mL, 21 mmol) was added, and the bottle was sealed with the screw cap. The total amount of solvent was increased only 10-fold instead of 14-fold to facilitate the subsequent workup. Metallic palladium precipitated on the bottle walls at the end of the reaction. Subsequently, the solution was filtered on a short pad of Celite in a Pasteur pipette using the cannula technique to get rid of any potential colloidal palladium particles. The solvent was evaporated, and the crude was recrystallized from methanol/CH_2_Cl_2_ 75:15 (80 mL). The product was collected by suction filtration on a Buchner funnel and dried under vacuum for several hours. **2d** was obtained as an analytically pure white solid.

### 3.4. Characterization Data for Quinolones

*2-Phenylquinolin-4(1H)-one* (**2a**) [70]. White solid (103 mg, 93% yield). ^1^H NMR (400 MHz, DMSO-*d*_6_) δ 11.70 (br, 1H), 8.11 (d, *J* = 8.0 Hz, 1H), 7.87–7.80 (m, 2H), 7.77 (d, *J* = 8.3 Hz, 1H), 7.67 (t, *J* = 7.6 Hz, 1H), 7.61–7.57 (m, 3H), 7.34 (t, *J* = 7.5 Hz, 1H), 6.33 (s, 1H) ppm. ^13^C NMR (100 MHz, DMSO-*d*_6_) δ 176.9, 150.0, 140.5, 134.2, 131.8, 130.4, 129.0, 127.4, 124.9, 124.7, 123.2, 118.7, 107.3 ppm.
*2-(4-Methylphenyl)quinolin-4(1H)-one* (**2b**) [71]. Pale orange solid (106 mg, 90% yield). ^1^H NMR (DMSO-*d*_6_, 400 MHz): δ 11.63 (br, 1H), 8.10 (dd, *J* = 8.1, 1.2 Hz, 1H), 7.77 (d, *J* = 8.4 Hz, 1H), 7.74 (d, *J* = 8.0 Hz, 2H), 7.66 (ddd, *J* = 8.4, 7.0, 1.5 Hz, 1H), 7.39 (d, *J* = 8.0 Hz, 2H), 7.33 (t, *J* = 7.4 Hz, 1H), 6.32 (s, 1H), 2.40 (s, 3H) ppm. ^13^C NMR (DMSO-*d*_6_, 100 MHz): δ 176.9, 149.9, 140.5, 140.3, 131.7, 131.3, 129.5, 127.2, 124.8, 124.7, 123.1, 118.7, 106.9, 20.9 ppm.
*2-(2,4,6-trimethylphenyl)quinolin-4(1H)-one* (**2c**) White solid (74 mg, 56%yield). ^1^H NMR (DMSO-*d*_6_, 400 MHz): δ 11.74 (br, 1H), 8.23–8.04 (m, 1H), 7.64 (ddd, *J* = 8.4, 7.0, 1.5 Hz, 1H), 7.56 (d, *J* = 8.0 Hz, 1H), 7.38–7.29 (m, 1H), 7.02 (s, 2H), 2.30 (s, 3H), 2.13 (s, 6H) ppm. ^13^C NMR (DMSO-*d*_6_, 100 MHz): δ 176.7, 149.8, 140.4, 138.4, 135.6, 132.1, 131.6, 128.1, 124.85, 124.81, 123.1, 118.3, 109.6, 20.6, 19.3 ppm. Elemental analysis for C_18_H_17_NO Calcd.: C, 81.10; H, 6.51; N, 5.32%. Found: C, 80.75; H, 6.73; N, 5.10%.
*2-(4-Methoxyphenyl)quinolin-4(1H)-one* (**2d**) [71] Off-white solid (117 mg, 93% yield). ^1^H NMR (DMSO-*d*_6_, 400 MHz): δ 11.62 (br, 1H), 8.10 (d, *J* = 7.7 Hz, 1H), 7.87–7.75 (m, 3H), 7.65 (t, *J* = 7.2 Hz, 1H), 7.32 (t, *J* = 7.0 Hz, 1H), 7.12 (d, *J* = 8.1 Hz, 2H), 6.32 (s, 1H), 3.84 (s, 3H) ppm. ^13^C NMR (DMSO-*d*_6_, 100 MHz): δ 176.8, 161.0, 149.7, 140.5, 131.6, 128.8, 126.2, 124.8, 124.7, 123.1, 118.6, 114.4, 106.5, 55.4 ppm.
*2-(3-Benzyloxy-4-methoxy-phenyl)quinolin-4(1H)-one* (**2e**) Pale pink solid (109 mg, 61% yield). ^1^H NMR (DMSO-*d*_6_, 400 MHz): δ 11.59 (br, 1H), 8.10 (dd, *J* = 8.0, 1.1 Hz, 1H), 7.78 (d, *J* = 8.2 Hz, 1H), 7.69–7.64 (m, 1H), 7.55–7.39 (m, 6H), 7.37–7.29 (m, 2H), 7.18 (d, *J* = 8.5 Hz, 1H), 6.38 (s, 1H), 5.23 (s, 2H), 3.86 (s, 3H) ppm. ^13^C NMR (DMSO-*d*_6_, 100 MHz): δ 176.8, 151.1, 149.8, 147.9, 140.5, 136.9, 131.7, 128.5, 127.94, 127.88, 126.4, 124.73, 124.67, 123.2, 120.5, 118.7, 112.6, 112.1, 106.6, 70.2, 55.8 ppm. Elemental analysis for C_23_H_19_NO_3_ Calcd.: C, 77.29; H, 5.36; N, 3.36 %. Found: C, 77.02; H, 5.08; N, 2.97%.
*2-(4-Fluorophenyl)quinolin-4(1H)-one* (**2g**) [71] Golden yellow solid (97 mg, 81% yield). ^1^H NMR (DMSO-*d*_6_, 400 MHz): δ 11.71 (br, 1H), 8.10 (ddd, *J* = 8.1, 1.5, 0.5 Hz, 1H), 7.91 (dd, *J* = 8.7, 5.6 Hz, 2H), 7.75 (d, *J* = 8.4 Hz, 1H), 7.67 (ddd, *J* = 8.4, 6.9, 1.5 Hz, 1H), 7.47–7.40 (m, 2H), 7.34 (ddd, *J* = 8.1, 7.0, 1.3 Hz, 1H), 6.33 (br, 1H)ppm. ^13^C NMR (DMSO-*d*_6_, 100 MHz): δ 176.9, 163.4 (d *^1^J_C-F_*, 248.0 Hz), 149.0, 140.5, 131.8, 129.9 (d *^3^J_C-F_*, 8.7 Hz), 124.7, 123.3, 118.7, 116.0 (d *^2^J_C-F_*, 21.8 Hz), 107.4 ppm.
*2-(4-Chlorophenyl)quinolin-4(1H)-one* (**2h**) [72]. Off-white solid (106 mg, 83% yield). ^1^H NMR (DMSO-*d*_6_, 400 MHz): δ 11.72 (br, 1H), 8.10 (d, *J* = 7.9 Hz, 1H), 7.87 (d, *J* = 8.2 Hz, 2H), 7.80–7.62 (m, 4H), 7.34 (t, *J* = 7.3 Hz, 1H), 6.35 (br, 1H) ppm. ^13^C NMR (DMSO-*d*_6_, 100 MHz): 176.9, 148.8, 140.5, 135.2, 131.9, 129.3, 129.0, 124.7, 123.4, 118.8, 107.5 ppm.
*2-(4-Bromophenyl)quinolin-4(1H)-one* (**2i**) [73]. Pink solid (109 mg, 73% yield). ^1^H NMR (DMSO-*d*_6_, 400 MHz): δ 11.72 (s, 1H), 8.10 (d, *J* = 7.5 Hz, 1H), 7.80 (s, 4H), 7.75 (d, *J* = 8.3 Hz, 1H), 7.69 (dd, *J* = 11.0, 4.1 Hz, 1H), 7.35 (t, *J* = 7.4 Hz, 1H) ppm. ^13^C NMR (DMSO-*d*_6_, 100 MHz): 177.0, 148.9, 140.5, 133.3, 131.9, 129.5, 124.9, 124.7, 124.0, 123.4, 118.7, 107.5 ppm.
*2-(4-Carbomethoxyphenyl)quinolin-4(1H)-one* (**2j**) [74]. Pink solid (116 mg, 83% yield). ^1^H NMR (DMSO-*d*_6_, 400 MHz): δ 11.85 (br, 1H), 8.18–8.08 (m, 2H), 8.00 (d, *J* = 7.8 Hz, 2H), 7.79 (d, *J* = 8.3 Hz, 1H), 7.70 (t, *J* = 7.1 Hz, 1H), 7.36 (t, *J* = 7.4 Hz, 1H), 6.41 (br, 1H), 3.92 (s, 3H) ppm. ^13^C NMR (DMSO-*d*_6_, 100 MHz): 176.9, 165.7, 148.7, 140.5, 138.5, 132.0, 131.1, 129.6, 127.9, 124.9, 124.7, 123.4, 118.8, 108.0, 52.4 ppm.
*2-(4-Cyanophenyl)quinolin-4(1H)-one* (**2k**) [75]. Light orange solid (68 mg, 55% yield). ^1^H NMR (DMSO-*d*_6_, 400 MHz): δ 11.84 (br, 1H), 8.12 (d, *J* = 7.9 Hz, 1H), 8.09–8.02 (m, 4H), 7.77 (d, *J* = 8.3 Hz, 1H), 7.70 (t, *J* = 7.2 Hz, 1H), 7.37 (t, *J* = 7.4 Hz, 1H), 6.45 (br, 1H) ppm. ^13^C NMR (DMSO-*d*_6_, 100 MHz): δ 132.8, 132.0, 128.4, 124.6, 123.6, 118.4, 112.8 ppm. Due to the low solubility of the compound in DMSO-*d*_6_, no quaternary carbon was detected.
*2-(4-Nitrophenyl)quinolin-4(1H)-one* (**2l**) [76]. Purified by column chromatography using hexane: EtOAc (7:3). Red solid (14 mg, 11% yield). ^1^H NMR (DMSO-*d*_6_, 400 MHz): δ 10.17 (br, 1H), 8.27 (d, *J* = 8.7 Hz, 2H), 8.01 (dd, *J* = 49.0, 8.6 Hz, 2H), 7.62 (d, *J* = 7.6 Hz, 1H), 7.54 (dt, *J* = 14.2, 6.8 Hz, 1H), 7.16 (d, *J* = 8.0 Hz, 1H), 6.98 (t, *J* = 7.4 Hz, 1H), 6.67 (s, 1H) ppm. ^13^C NMR (DMSO-*d*_6_, 100 MHz): δ 186.6, 154.3, 146.0, 141.3, 137.0, 136.3, 130.4, 124.5, 123.9, 120.5, 119.7, 112.7, 106.1 ppm.
*2-(Anthracen-9-yl)quinolin-4(1H)-one* (**2m**) [75]. Tan solid (50 mg, 31%yield). ^1^H NMR (DMSO-*d*_6_, 400 MHz): δ 12.22 (br, 1H), 8.84 (s, 1H), 8.28 (dd, *J* = 8.1, 1.2 Hz, 1H), 8.24–8.19 (m, 2H), 7.80 (d, *J* = 8.3 Hz, 2H), 7.72 (ddd, *J* = 8.4, 7.1, 1.5 Hz, 1H), 7.64–7.52 (m, 5H), 7.47–7.41 (m, 1H), 6.20 (s, 1H) ppm. ^13^C NMR (DMSO-*d*_6_, 100 MHz):176.7, 147.9, 140.7, 132.0, 130.6, 129.1, 128.73, 128.74 128.64, 128.57, 127.2, 125.7, 125.3, 125.0, 123.5, 118.5, 112.1 ppm.
*2-(1-Methyl-1H-pyrrol-2-yl)quinolin-4(1H)-one* (**2n**) Purified by column chromatography using hexane: EtOAc (6:4). Dark solid (70 mg, 62% yield). ^1^H NMR (DMSO-*d*_6_, 400 MHz): δ 9.31 (br, 1H), 7.55 (d, *J* = 7.6 Hz, 1H), 7.47 (ddd, *J* = 8.3, 7.3, 1.3 Hz, 1H), 7.15 (d, *J* = 8.2 Hz, 1H), 7.06 (dd, *J* = 2.3, 1.6 Hz, 1H), 6.96 (ddd, *J* = 3.9, 1.6, 0.6 Hz, 1H), 6.88 (ddd, *J* = 7.6, 7.1, 0.7 Hz, 1H), 6.66 (br, 1H), 6.26 (ddd, *J* = 3.9, 2.6, 0.6 Hz, 1H), 3.74 (s, 3H) ppm. ^13^C NMR (DMSO-*d*_6_, 100 MHz): δ 184.8, 152.7, 135.3, 130.9, 127.6, 127.3, 123.6, 120.6, 119.2, 113.7, 112.5, 109.5, 99.8, 33.7 ppm. Elemental analysis for C_14_H_12_N_2_O Calcd.: C, 74.98; H, 5.39; N, 12.49%. Found: C, 75.34; H, 5.06; N, 12.67%
*2-(5-Methylfuran-2-yl)quinolin-4(1H)-one* (**2o**) Purified by column chromatography using hexane: EtOAc (8:2). Orange solid (40 mg, 35% yield). ^1^H NMR (CDCl_3_, 400 MHz): δ 7.70 (d, *J* = 7.7 Hz, 1H), 7.62 (br, 1H), 7.47–7.33 (m, 1H), 6.99 (d, *J* = 8.1 Hz, 1H), 6.94–6.84 (m, 1H), 6.60 (s, 1H), 6.53 (d, *J* = 3.3 Hz, 1H), 6.16–6.10 (m, 1H), 2.43 (s, 3H) ppm. ^13^C NMR (CDCl_3_, 100 MHz): δ 185.8, 155.1, 152.0, 150.6, 135.8, 132.4, 124.8, 121.5, 120.0, 116.5, 111.5, 109.6, 99.0, 14.4 ppm. Elemental analysis for C_14_H_11_NO_2_ Calcd.: C, 74.65; H, 4.92; N, 6.22%. Found: C, 74.27; H, 5.11; N, 5.97%.
*6-Phenyl-[1,3]dioxolo[4,5-g]quinolin-8(5H)-one* (**2p**) [77]. Pink solid (105 mg, 79% yield). ^1^H NMR (DMSO-*d*_6_, 400 MHz): δ 11.58 (br, 1H), 7.80 (br, 3H), 7.57 (m, 3H), 7.40 (s, 1H), 7.20 (s, 1H), 6.27 (br, 1H), 6.15 (s, 3H) ppm. ^13^C NMR (DMSO-*d*_6_, 100 MHz): δ 175.8, 151.1, 148.5, 145.20, 137.4, 134.2, 130.2, 129.0, 127.2, 120.4, 106.5, 101.9, 101.2, 97.2 ppm.
*6-(4-Methylphenyl)-[1,3]dioxolo[4,5-g]quinolin-8(5H)-one* (**2q**) [78]. Pink solid (126 mg, 90% yield). ^1^H NMR (DMSO-*d*_6_, 400 MHz): δ 11.51 (s, 1H), 7.70 (d, *J* = 8.2 Hz, 2H), 7.38 (m, 3H), 7.21 (s, 1H), 6.24 (d, *J* = 1.8 Hz, 1H), 6.15 (s, 2H), 2.40 (s, 3H) ppm. ^13^C NMR (DMSO-*d*_6_, 100 MHz): δ 175.7, 151.0, 148.5, 145.0, 140.1, 137.4, 131.2, 129.5, 127.0, 120.4, 106.2, 101.9, 101.2, 97.2, 20.9 ppm.
*6-Chloro-2-(4-methylphenyl)quinolin-4(1H)-one* (**2r**) Pink solid (95 mg, 70% yield). ^1^H NMR (DMSO-*d*_6_, 400 MHz): δ 11.81 (br, 1H), 8.03 (d, *J* = 2.4 Hz, 1H), 7.81 (d, *J* = 8.9 Hz, 1H), 7.75 (d, *J* = 8.0 Hz, 2H), 7.71 (dd, *J* = 8.9, 2.4 Hz, 1H), 7.40 (d, *J* = 8.0 Hz, 2H), 6.38 (br, 1H), 2.41 (s, 3H) ppm. ^13^C NMR (DMSO-*d*_6_, 100 MHz): δ 150.3, 140.5, 131.8, 131.0, 129.57, 127.8, 127.25, 123.58, 107.00, 20.89 ppm. Due to the low solubility of the compound in DMSO, four quaternary carbons were not detected. Elemental analysis for C_16_H_12_ClNO Calcd.: C, 71.25; H, 4.48; N, 5.19%. Found: C, 71.04; H, 4.66; N, 4.93%
*2-(Benzo[d][1,3]dioxol-5-yl)quinolin-4(1H)-one* (**2s**) [72]. Colorless solid (98 mg, 74% yield). ^1^H NMR (DMSO-*d*_6_, 400 MHz): δ 11.56 (br, 1H), 8.08 (dd, *J* = 8.0, 1.0 Hz, 1H), 7.75 (d, *J* = 8.2 Hz, 1H), 7.70–7.62 (m, 1H), 7.42 (d, *J* = 1.6 Hz, 1H), 7.37 (dd, *J* = 8.1, 1.7 Hz, 1H), 7.32 (t, *J* = 7.3 Hz, 1H), 7.11 (d, *J* = 8.1 Hz, 1H), 6.30 (s, 1H), 6.14 (s, 2H) ppm. ^13^C NMR (DMSO-*d*_6_, 100 MHz): δ 176.9, 149.5, 149.2, 147.9, 140.4, 131.7, 128.0, 124.8, 124.7, 123.1, 121.8, 118.6, 108.7, 107.6, 106.8, 101.8 ppm.

### 3.5. Synthesis of Graveoline from ***2s***

Norgraveoline **2s** was prepared and isolated according to the general procedure described above. In an oven-dried Schlenk flask, **2s** (50 mg, 0.19 mmol), potassium carbonate (52 mg, 0.38 mmol) and methyl iodide (40 mg, 0.28 mmol) were dissolved in acetone (5 mL) and refluxed for 5 h. After reaction completion, acetone was removed by rotary evaporation, and the crude was purified by filtration over silica-gel using hexane/ethyl acetate (1:9) as the eluent to produce the final product as yellow crystals (46.5 mg, 0.17 mmol, 88%). ^1^H NMR (CDCl_3_, 400 MHz): δ 8.17 (dd, *J* = 8.3, 1.0 Hz, 1H), 8.06 (d, *J* = 8.2 Hz, 1H), 7.74–7.66 (m, 2H), 7.62 (dd, *J* = 8.1, 1.8 Hz, 1H), 7.46 (ddd, *J* = 8.2, 6.9, 1.1 Hz, 1H), 7.10 (s, 1H), 6.94 (d, *J* = 8.1 Hz, 1H), 6.04 (s, 2H), 4.11 (s, 3H) ppm. ^13^C NMR (CDCl_3_, 100 MHz): δ 162.9, 158.38, 149.3, 148. 9, 148.4, 135.0, 130.1, 129.2, 125.3, 121.8, 121.8, 120.4, 108.5, 108.2, 101.5, 97.7, 55.8 ppm. Elemental analysis for C_17_H_13_NO_3_ Calcd.: C, 73.11; H, 4.69; N, 5.02%. Found: C, 73.16; H, 4.57; N, 5.14%.

## 4. Conclusions

In this paper, we reported a new protocol for the synthesis of 2-aryl-4-quinolones that, despite employing carbon monoxide as a reducing agent, can be performed without employing any gaseous reagent. Rather, cheap formic acid activated by acetic anhydride is employed as a CO surrogate, and the reaction can be performed in an economical pressure tube or flask, which are easily available in different dimensions from a few milliliters to about 1 L. The only stoichiometric byproducts are CO_2_ and acetic acid, both of which are easily separable from the other products of the reaction. The reaction tolerated different types of substituents, and the isolated yields were higher than those previously obtained using pressurized CO, demonstrating the efficiency of the CO surrogate employed.

## Figures and Tables

**Table 1:** Synthesis of 2-phenylquinolin-4(1*H*)-one (**2a**) by Pd-catalyzed reductive cyclization of 2′-nitrochalcone (**1a**) using HCOOH/Ac_2_O as a CO source: optimization of the reaction conditions ^1^.

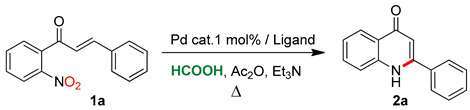							
**Entry**	**T (°C)**	**t (h)**	**Solvent**	**FA/Ac_2_O/Et_3_N to 1a mol Ratio**	**Ligand**	**Conv. % ^2^**	**Select. % ^3^**
1 ^4^	110	10	Acetone	2.5	Phen	98	51
2	140	10	CH_3_CN	4.4	Phen	>99	69
3	140	10	CH_3_CN	3	Phen	96	76
4	140	10	CH_3_CN	2.5	Phen	84	62
5 ^5^	140	10	CH_3_CN	3	Phen	37	24
6	140	10	CH_3_CN	3	TMPhen	>99	47
7 ^6^	140	10	CH_3_CN	3	(MeO)_2_Phen	98	58
8	150	10	CH_3_CN	3	Phen	100	74
9	130	10	CH_3_CN	3	Phen	84	80
10	120	16	CH_3_CN	3	Phen	>99	55
11 ^6^	130	10	CH_3_CN	3	Phen	57	85
12 ^6^	130	16	CH_3_CN	3	Phen	96	76
13 ^6^	130	16	CH_3_CN/DMF ^7^	3	Phen	>99	75
14 ^6^	130	16	MEK	3	Phen	64	72
15 ^6^	130	16	DMF	3	Phen	>99	>99
**16 ^6^**	**130**	**10**	**DMF**	**3**	**Phen**	**99**	**>99**
17 ^6^	130	6	DMF	3	Phen	88	93
18 ^6^	130	4	DMF	3	Phen	59	93

^1^ Experimental conditions: **1a** (127 mg, 0.5 mmol), Pd(CH_3_CN)_2_Cl_2_ (1 mol%), ligand (5 mol%), solvent (10 mL). Equal molar amounts of formic acid (FA), Ac_2_O, and Et_3_N are always employed. Best conditions highlighted in bold. ^2^ Calculated with respect to the starting **1a**, measured by HPLC. ^3^ Calculated with respect to the reacted **1a**, measured by HPLC. ^4^ 1 mol% Pd(acac)_2_. ^5^ The reaction was assembled in the air. ^6^ Ligand 2.5 mol%. ^7^ CH_3_CN/DMF = 9:1.

## Data Availability

^1^H and ^13^C NMR spectra of the new 2′-nitrochalcones and all quinolones are reported in the Supporting Information.

## Supplementary Materials

The following supporting information can be downloaded at https://www.mdpi.com/article/10.3390/molecules28145424/s1, Procedures employed for the preparation of previously unreported nitrochalcones. Copies of ^1^H and ^13^C NMR spectra of the new chalcones and all prepared quinolones. References [79,80] are cited in the Supplementary Materials.
