# Supplementary material for: Effective Synthesis of 4-Quinolones by Reductive Cyclization of 2′-Nitrochalcones Using Formic Acid as a CO Surrogate
Source: Molecules. 2023 Jul 15;28(14):5424. doi: 10.3390/molecules28145424 (PMC10386197; doi:10.3390/molecules28145424)
Supplement: Supplementary file 1 [file molecules-28-05424-s001.zip › molecules-2494174-supplementary.pdf]

Supplementary Materials for:

Effective Synthesis of 4-Quinolones by Reductive Cyclization of 2′-Nitrochalcones Using Formic Acid as a CO Surrogate

Francesco Ferretti [1], Manar Ahmed Fouad [1,2], Cecilia Abbo [1] and Fabio Ragaini [1,*]

[1] Dipartimento di Chimica, Università Degli Studi di Milano, Via C. Golgi 19, 20133 Milano, Italy; francesco.ferretti@unimi.it (F.F.); manar.abdellatif@unimi.it (M.A.F.);
[2] Chemistry Department, Faculty of Science, Alexandria University, P.O. Box 426, Alexandria 21321, Egypt
* Correspondence: fabio.ragaini@unimi.it

Summary

1. Synthesis of 2'-nitrochalcones (1a-r) 1
2. NMR spectra 7
3. References 36

1. Synthesis of 2'-nitrochalcones (1a-r)

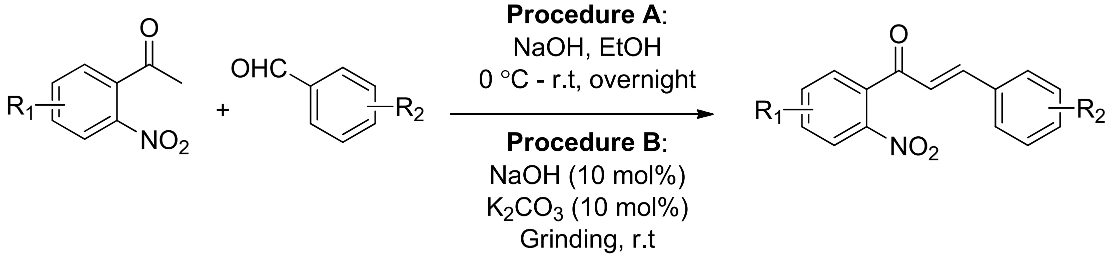

General Procedure A

Under nitrogen atmosphere, 2-nitroacetophenone derivative (3 mmol) was dissolved in ethanol (5 mL), then a solution of sodium hydroxide (3 mmol) in ethanol (5 mL) was added dropwise at 0 °C. Subsequently a solution of the benzaldehyde derivative (3 mmol) in ethanol (5 mL) was added to the reaction mixture over 30 minutes at 0 °C. The reaction was stirred at room temperature overnight to allow the precipitation of the desired product. The product was recovered by filtration and washed with cold ethanol and hexane.

General Procedure B

In a dry mortar, benzaldehyde derivative (3 mmol) was grinded with potassium carbonate (0.3 mmol) and sodium hydroxide (0.3 mmol). 2-Nitroacetophenone derivative (3 mmol) was added dropwise at room temperature. After reaction completion (followed by TLC), water (15 mL) and ethyl acetate (15 mL) were added, and the mixture transferred to a separating funnel. The organic layer was separated, and the aqueous phase extracted with ethyl acetate (3 × 15 mL). The combined organic layer was washed with water (3 × 50 mL) and brine (50 mL), dried over Na_2SO_4, filtered and the organic solvent removed under reduced pressure to afford the crude product. The crude was recrystallized from methanol or purified by column chromatography to give the final product.

2′-Nitrochalcones **1a**, **b**, **d**, **f-i** were prepared according to general procedure A and their NMR analyses were in accordance to those previously reported in the literature.[1] **1k** was prepared following the general procedure B and its analytical data are in accordance to those previously reported in the literature.[2] The analyses for the other starting compounds are reported below.

(*E*)-3-mesityl-1-(2-nitrophenyl)prop-2-en-1-one (1c)

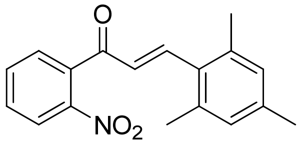

Prepared according to general procedure **A**. Yellow solid (40% yield), ^{1}H NMR ($CDCl_3$, 400 MHz): δ 8.18 (dd, *J* = 8.2, 0.8 Hz, 1H), 7.77 (td, *J* = 7.5, 1.1 Hz, 1H), 7.65 (td, *J* = 8.2, 1.4 Hz, 1H), 7.54 (dd, *J* = 7.5, 1.3 Hz, 1H), 7.38 (d, *J* = 16.6 Hz, 1H), 6.88 (s, 2H), 6.62 (d, *J* = 16.6 Hz, 1H), 2.27 (d, *J* = 4.0 Hz, 9H) ppm.^{13}C NMR ($CDCl_3$, 100 MHz): δ 193.28, 146.90, 145.21, 139.23, 137.03, 136.42, 134.19, 131.55, 130.69, 130.48, 129.43, 128.89, 124.55, 21.17, 21.07 ppm. Elemental Analysis for $C_{18}H_{17}NO_3$ Calcd.: C, 73.20; H, 5.80; N, 4.74%. Found: C, 72.97; H, 5.96; N, 4.68%.

(*E*)-3-(3-(benzyloxy)-4-methoxyphenyl)-1-(2-nitrophenyl)prop-2-en-1-one (1e)

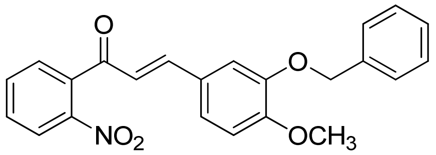

Prepared according to general procedure **A**. Yellow solid (91% yield), ^{1}H NMR ($CDCl_3$, 400 MHz): δ 8.16 (d, *J* = 8.2 Hz, 1H), 7.74 (t, *J* = 7.5 Hz, 1H), 7.63 (t, *J* = 7.8 Hz, 1H), 7.48 (d, *J* = 7.5 Hz, 1H), 7.44 (d, *J* = 7.5 Hz, 2H), 7.38 (t, *J* = 7.4 Hz, 2H), 7.33 (d, *J* = 7.2 Hz, 1H), 7.15 (d, *J* = 16.2 Hz, 1H), 7.11 – 7.03 (m, 2H), 6.87 (d, *J* = 8.1 Hz, 1H), 6.80 (d, *J* = 16.1 Hz, 1H), 5.15 (s, 2H), 3.91 (s, 3H) ppm.^{13}C NMR ($CDCl_3$, 100 MHz): δ 192.9, 152.73, 148.63, 146.94, 146.63, 136.68, 134.05, 130.54, 129.00, 128.79, 128.23, 127.52, 126.96, 124.66, 124.30, 124.12, 112.91, 111.66, 71.29, 56.19 ppm. Elemental Analysis for $C_{23}H_{19}NO_5$ Calcd.: C, 70.94; H, 4.92; N, 3.60%. Found: C, 71.07; H, 4.94; N, 3.65%.

(*E*)-methyl 4-(3-(2-nitrophenyl)-3-oxoprop-1-en-1-yl)benzoate (1j)

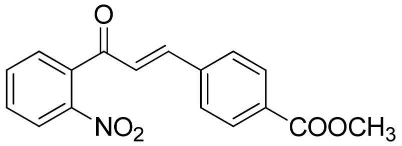

Prepared according to general procedure **B**. White solid (68% yield), ^{1}H NMR ($CDCl_3$, 400 MHz): δ 8.19 (d, *J* = 8.2 Hz, 1H), 8.03 (d, *J* = 7.1 Hz, 2H), 7.78 (t, *J* = 7.5 Hz, 1H), 7.67 (t, *J* = 7.9 Hz, 1H), 7.55 (d, *J* = 8.1 Hz, 2H), 7.51 (d, *J* = 7.4 Hz, 1H), 7.27 (d, *J* = 16.3 Hz, 1H, overlapped with $CDCl_3$), 7.05 (d, *J* = 16.3 Hz, 1H), 3.92 (s, 3H) ppm.^{13}C NMR ($CDCl_3$, 100 MHz): δ 192.62, 166.41, 146.82, 144.40, 138.27, 136.26, 134.31, 132.10, 130.93, 130.27, 128.92, 128.48, 128.30, 124.74, 52.46 ppm. Elemental Analysis for $C_{17}H_{13}NO_5$ Calcd.: C, 65.59; H, 4.21; N, 4.50%. Found: C, 65.58; H, 4.35; N, 4.51%.

(*E*)-1-(2-nitrophenyl)-3-(4-nitrophenyl)prop-2-en-1-one (1l)

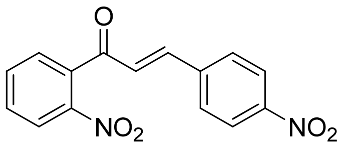

Prepared according to general procedure **B**. Yellow solid (42% yield), ^{1}H NMR ($CDCl_3$, 400 MHz): δ 8.26 - 8.12 (m, 3H), 7.80 (td, *J* = 7.5, 1.0 Hz, 1H), 7.70 (td, *J* = 8.0, 1.4 Hz 1H), 7.67 (d, *J* = 8.8 Hz, 2H), 7.52 (dd, *J* = 7.5, 1.3 Hz, 1H), 7.31 (d, *J* = 16.3 Hz, 1H), 7.08 (d, *J* = 16.3 Hz, 1H) ppm.^{13}C NMR ($CDCl_3$, 100 MHz): δ 192.11, 148.92, 146.76, 142.28, 140.22, 136.00, 134.48, 131.19, 129.74, 129.21, 128.89, 124.79, 124.32 ppm. Elemental Analysis for $C_{15}H_{10}N_2O_5$ Calcd.: C, 60.41; H, 3.38; N, 9.39%. Found: C, 60.32; H, 3.53; N, 9.66%.

(*E*)-3-(anthracen-9-yl)-1-(2-nitrophenyl)prop-2-en-1-one (1m)

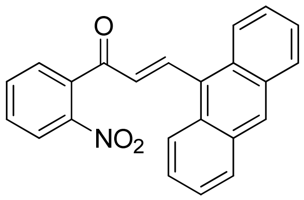

Prepared according to general procedure **B**. Yellow solid (90% yield), ^{1}H NMR ($CDCl_3$, 400 MHz): δ ^{1}H NMR (400 MHz, $CDCl_3$) δ 8.45 (d, *J* = 6.5 Hz, 1H), 8.26 (s, 1H), 8.23 (d, *J* = 8.8 Hz, 1H), 8.12 (d, *J* = 8.5 Hz, 2H), 8.00 (d, *J* = 8.1 Hz, 2H), 7.85 (t, *J* = 7.5 Hz, 1H), 7.70 (t, *J* = 7.8 Hz, 2H), 7.58 – 7.43 (m, 4H), 6.99 (d, *J* = 16.5 Hz, 1H) ppm.^{13}C NMR ($CDCl_3$, 100 MHz): δ 192.49, 146.98, 143.39, 136.55, 135.26, 134.47, 131.31, 130.97, 129.46, 129.13, 129.06, 128.97, 128.74, 126.89, 125.60, 124.96, 124.81 ppm. Elemental Analysis for $C_{23}H_{15}NO_3$ Calcd.: C, 78.17; H, 4.28; N, 3.96%. Found: C, 78.56; H, 4.19; N, 4.09%.

(*E*)-3-(1-methyl-1H-pyrrol-2-yl)-1-(2-nitrophenyl)prop-2-en-1-one (1n)

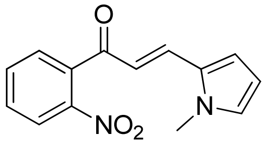

Prepared according to general procedure **A**. Yellow solid (44% yield), ^{1}H NMR ($CDCl_3$, 400 MHz): δ 8.09 (dd, *J* = 8.2, 0.9 Hz, 1H), 7.72 (td, *J* = 7.5, 1.1 Hz, 1H), 7.60 (td, *J* = 8.0, 1.4 Hz, 1H), 7.51 (dd, *J* = 7.5, 1.3 Hz, 1H), 7.36 (d, *J* = 15.7 Hz, 1H), 6.84 – 6.78 (m, 1H), 6.75 – 6.72 (m, 1H), 6.71 (d, *J* = 15.6 Hz, 1H), 6.19 (dd, *J* = 3.8, 2.7 Hz, 1H), 3.65 (s, 3H) ppm.^{13}C NMR ($CDCl_3$, 100 MHz): δ 191.82, 147.18, 137.11, 133.80, 133.68, 130.45, 129.38, 129.03, 128.90, 124.50, 119.88, 114.41, 110.29, 34.60 ppm. Elemental Analysis for $C_{14}H_{12}N_2O_3$ Calcd.: C, 65.62; H, 4.72; N, 10.93%. Found: C, 65.58; H, 4.83; N, 11.12%.

(*E*)-3-(5-methylfuran-2-yl)-1-(2-nitrophenyl)prop-2-en-1-one (1o)

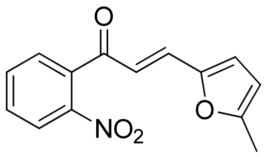

Prepared according to general procedure **A**. White solid (66% yield), ^{1}H NMR ($CDCl_3$, 400 MHz): δ 8.17 – 8.07 (m, 1H), 7.72 (tdd, *J* = 7.5, 2.2, 1.2 Hz, 1H), 7.63 – 7.60 (m, 1H), 7.54 – 7.44 (m, 1H), 7.01 (dd, *J* = 15.8, 3.0 Hz, 1H), 6.79 (dd, *J* = 15.8, 2.7 Hz, 1H), 6.60 – 6.54 (m, 1H), 6.11 – 6.10 (m, 1H), 2.34 (d, *J* = 3.0 Hz, 3H) ppm.^{13}C NMR ($CDCl_3$, 100 MHz): δ 192.26, 156.97, 149.33, 146.90, 136.76, 133.98, 132.19, 130.52, 128.89, 124.59, 121.57, 119.16, 109.73, 14.08 ppm. Elemental Analysis for $C_{14}H_{11}NO_4$ Calcd.: C, 65.37; H, 4.31; N, 5.44%. Found: C, 65.25; H, 4.70; N, 5.09%.

(*E*)-1-(6-nitrobenzo[d][1,3]dioxol-5-yl)-3-phenylprop-2-en-1-one (1p)

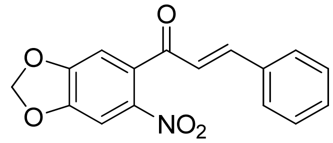

Prepared according to general procedure **A**. White solid (90% yield), ^{1}H NMR ($CDCl_3$, 400 MHz): δ 7.63 (d, *J* = 2.1 Hz, 1H), 7.54 – 7.44 (m, 2H), 7.40-7.36 (m, 3H), 7.24 (d, *J* = 17.2 Hz, 1H, overlapped with $CDCl_3$), 6.93 (d, *J* = 16.2 Hz, 1H), 6.83 (s, 1H), 6.21 (s, 2H) ppm.^{13}C NMR ($CDCl_3$, 100 MHz): δ 192.39, 152.69, 149.10, 145.76, 141.35, 134.13, 133.23, 131.11, 129.13, 128.65, 126.47, 107.79, 105.20, 103.77 ppm. Elemental Analysis for $C_{16}H_{11}NO_5$ Calcd.: C, 64.65; H, 3.73; N, 4.71%. Found: C, 64.88; H, 3.95; N, 4.52%.

(*E*)-1-(6-nitrobenzo[d][1,3]dioxol-5-yl)-3-(p-tolyl)prop-2-en-1-one (1q)

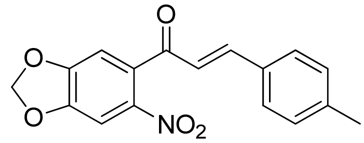

Prepared according to general procedure **A**. Yellow solid (72% yield), ^{1}H NMR ($CDCl_3$, 400 MHz): δ 7.62 (s, 1H), 7.38 (d, *J* = 7.6 Hz, 2H), 7.25-7.17 (m, 3H), 6.89 (d, *J* = 16.2 Hz, 1H), 6.82 (s, 1H), 6.20 (s, 2H), 2.37 (s, 3H) ppm.^{13}C NMR ($CDCl_3$, 100 MHz): δ 192.50, 152.63, 149.01, 146.01, 141.78, 141.33, 133.35, 131.40, 129.88, 128.69, 125.50, 107.81, 105.19, 103.73, 21.67 ppm. Elemental Analysis for $C_{17}H_{13}NO_5$ Calcd.: C, 65.59; H, 4.21; N, 4.50%. Found: C, 65.83; H, 4.56; N, 4.39%.

(*E*)-1-(5-chloro-2-nitrophenyl)-3-(p-tolyl)prop-2-en-1-one (2r)

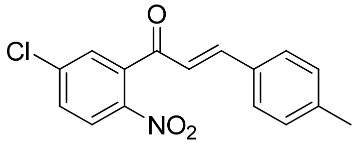

Prepared according to general procedure **A**. White solid (55% yield), ^{1}H NMR ($CDCl_3$, 400 MHz): δ 8.14 (d, *J* = 8.8 Hz, 1H), 7.59 (dd, *J* = 8.8, 2.3 Hz, 1H), 7.45 (d, *J* = 2.2 Hz, 1H), 7.40 (d, *J* = 8.1 Hz, 2H), 7.24 – 7.17 (m, 3H), 6.94 (d, *J* = 16.2 Hz, 1H), 2.37 (s, 3H) ppm.^{13}C NMR ($CDCl_3$, 100 MHz): δ 191.40, 147.17, 145.01, 142.18, 140.99, 138.17, 131.16, 130.58, 129.94, 128.98, 128.83, 126.17, 124.91, 21.69 ppm. Elemental Analysis for $C_{16}H_{12}ClNO_3$ Calcd.: C, 63.69; H, 4.01; N, 4.64%. Found: C, 63.92; H, 3.91; N, 4.78%.

2. NMR spectra

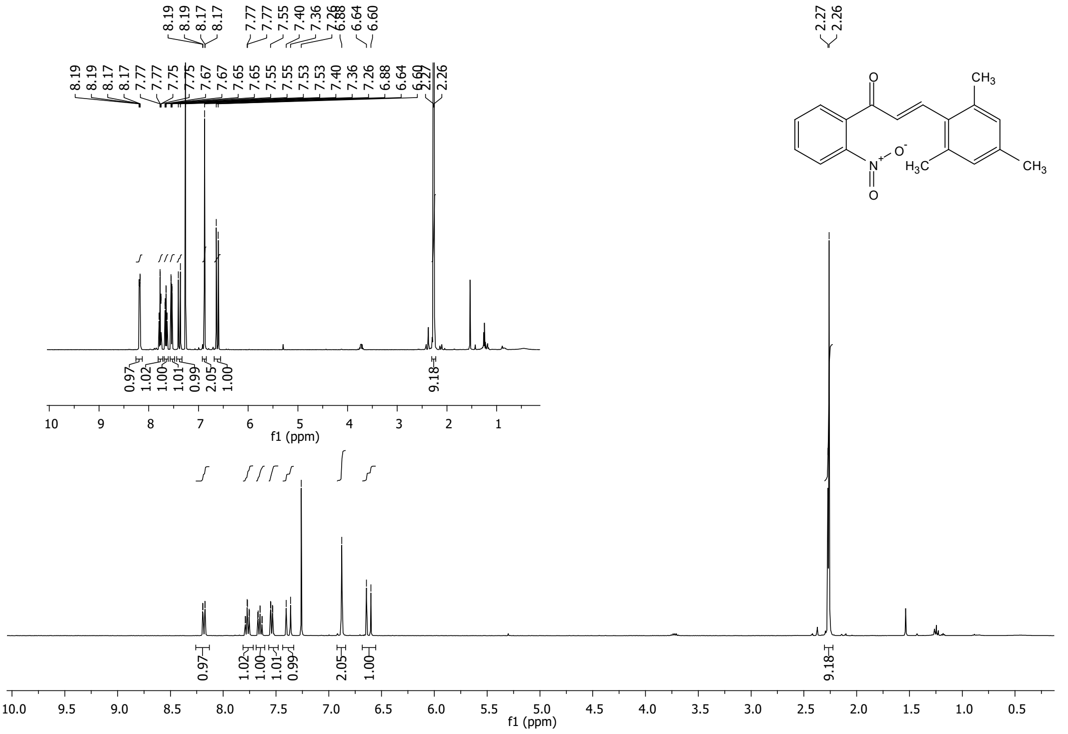

Figure S1. ^{1}H NMR of (*E*)-3-mesityl-1-(2-nitrophenyl)prop-2-en-1-one (**1c**).

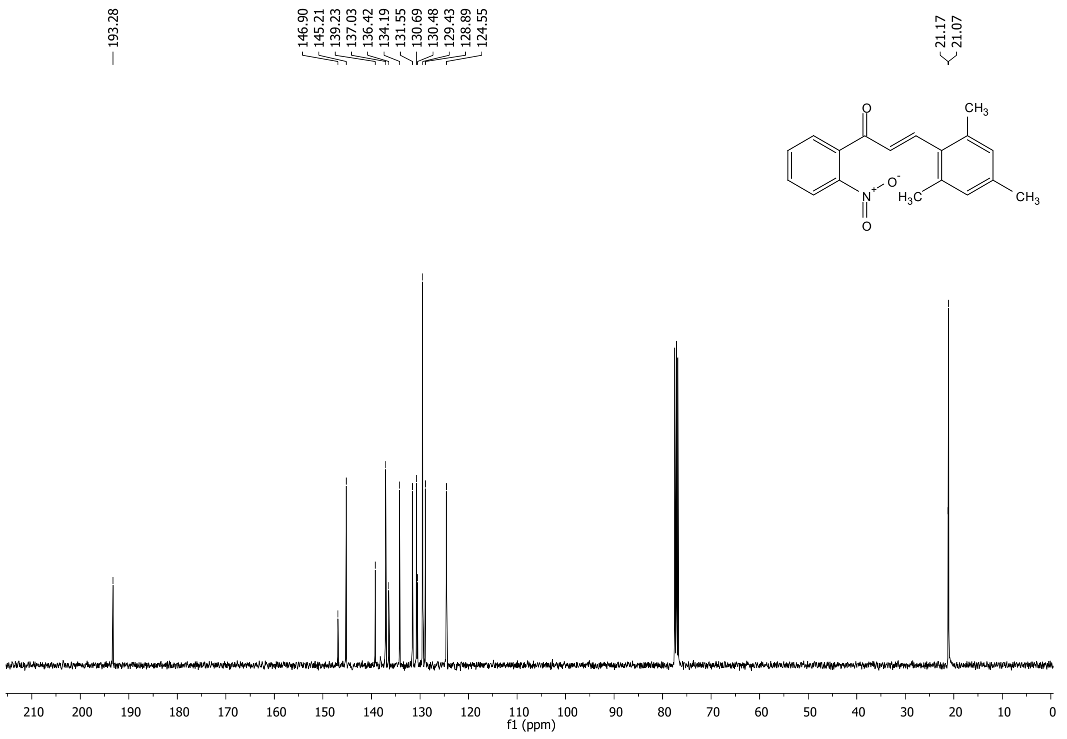

Figure S2. ^{13}C NMR of (*E*)-3-mesityl-1-(2-nitrophenyl)prop-2-en-1-one (**1c**).

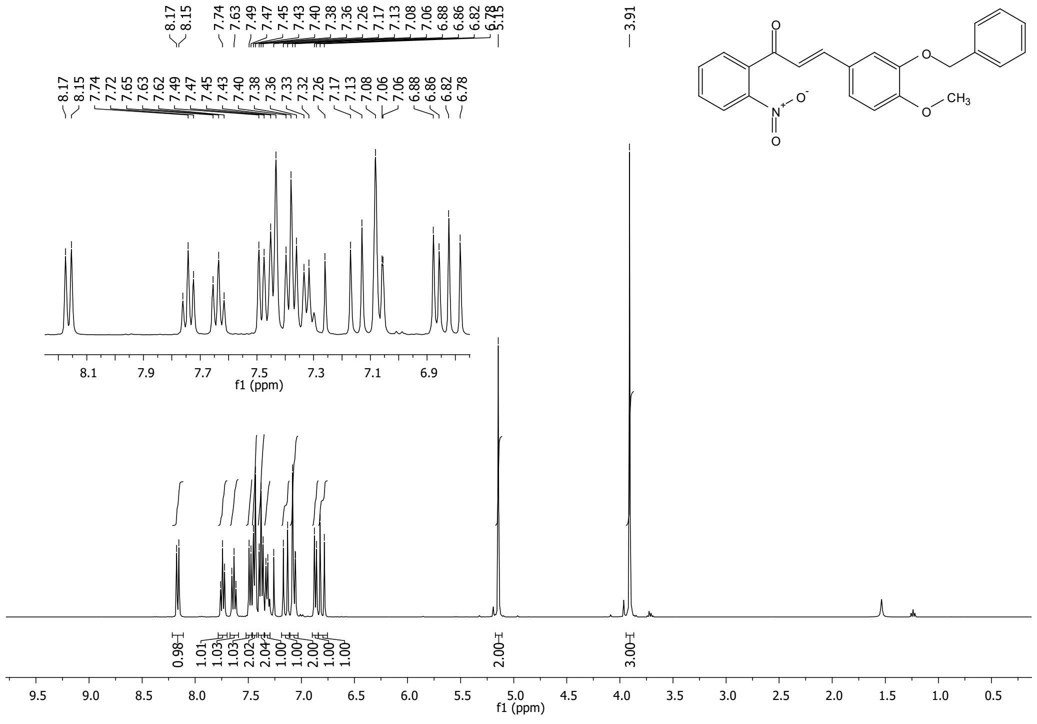

Figure S3. ^{1}H NMR of (*E*)-3-(3-(benzyloxy)-4-methoxyphenyl)-1-(2-nitrophenyl)prop-2-en-1-one (**1e**).

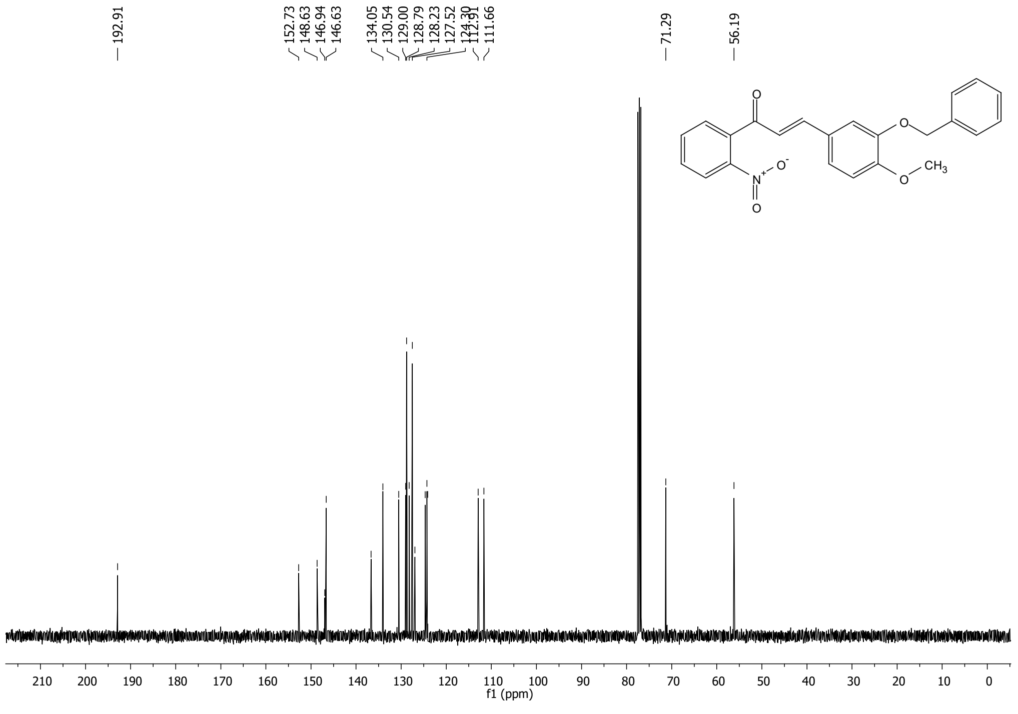

Figure S4. ^{13}C NMR of (*E*)-3-(3-(benzyloxy)-4-methoxyphenyl)-1-(2-nitrophenyl)prop-2-en-1-one (**1e**).

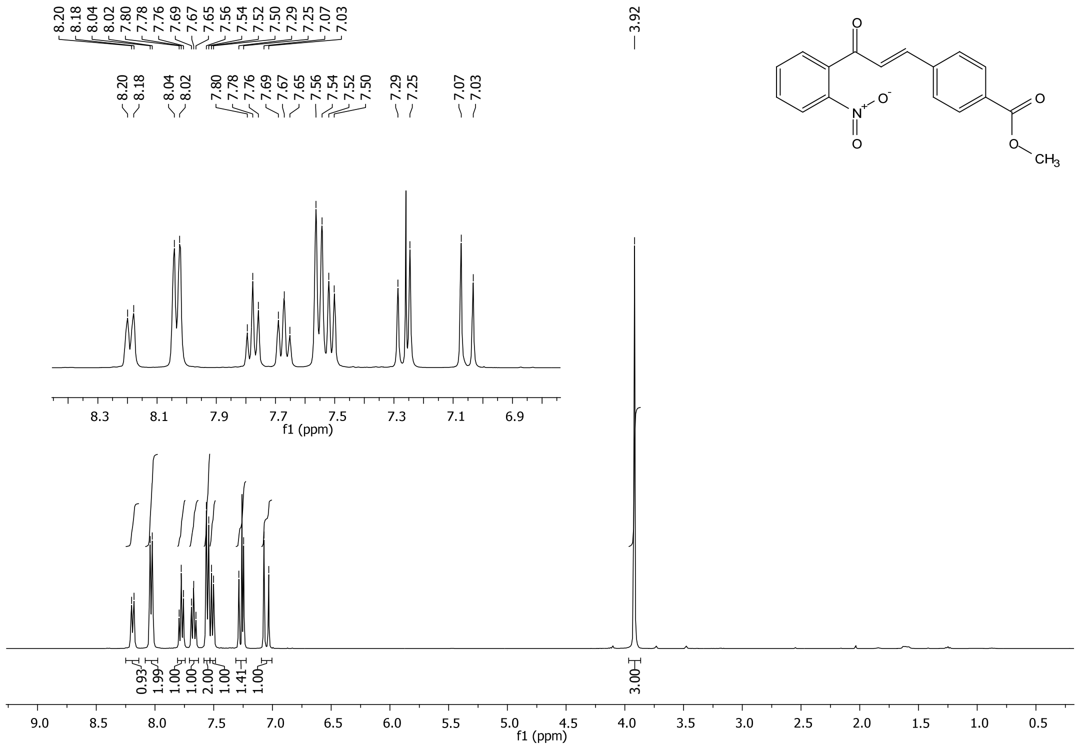

Figure S5. ^{1}H NMR of (*E*)-methyl 4-(3-(2-nitrophenyl)-3-oxoprop-1-en-1-yl)benzoate (**1j**).

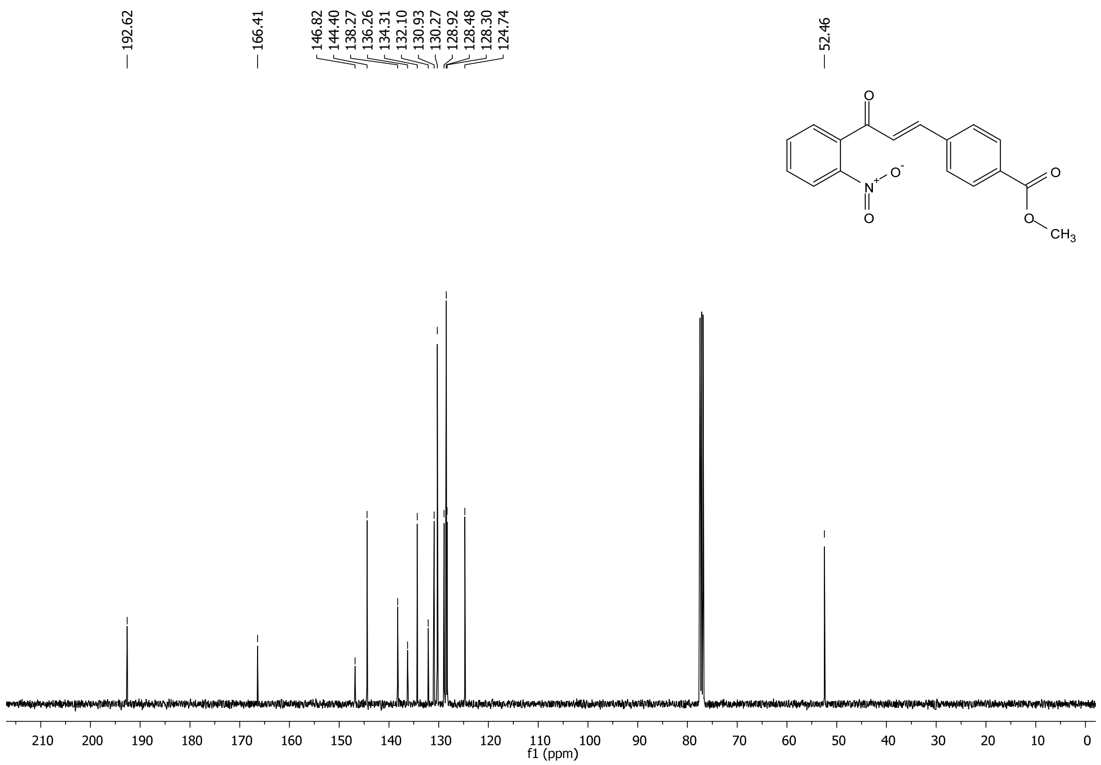

Figure S6. ^{13}C NMR of (*E*)-methyl 4-(3-(2-nitrophenyl)-3-oxoprop-1-en-1-yl)benzoate (**1j**).

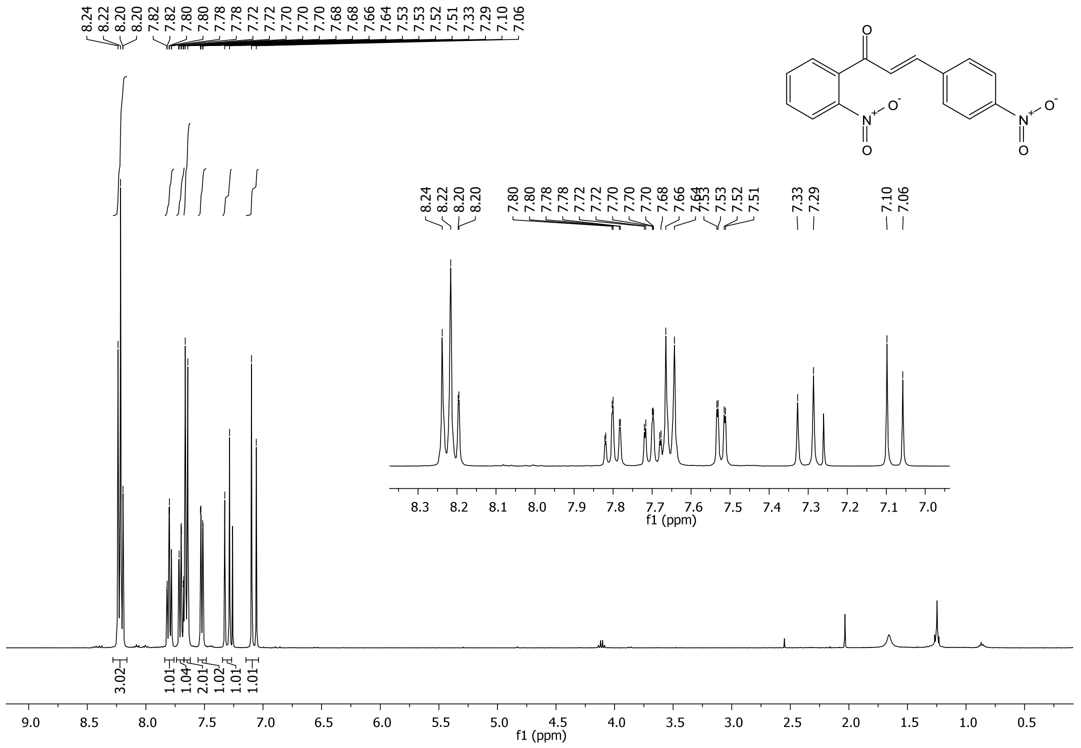

Figure S7. ^{1}H NMR of (*E*)-1-(2-nitrophenyl)-3-(4-nitrophenyl)prop-2-en-1-one (**1l**).

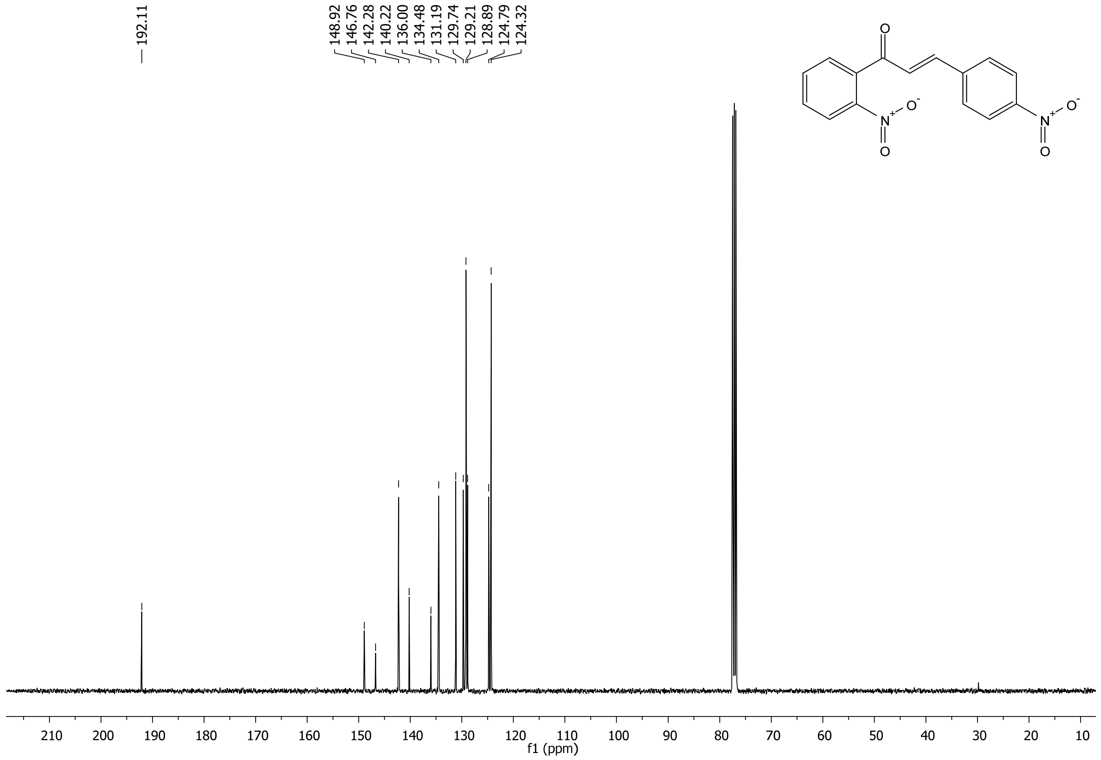

Figure S8. ^{13}C NMR of (*E*)-1-(2-nitrophenyl)-3-(4-nitrophenyl)prop-2-en-1-one (**1l**).

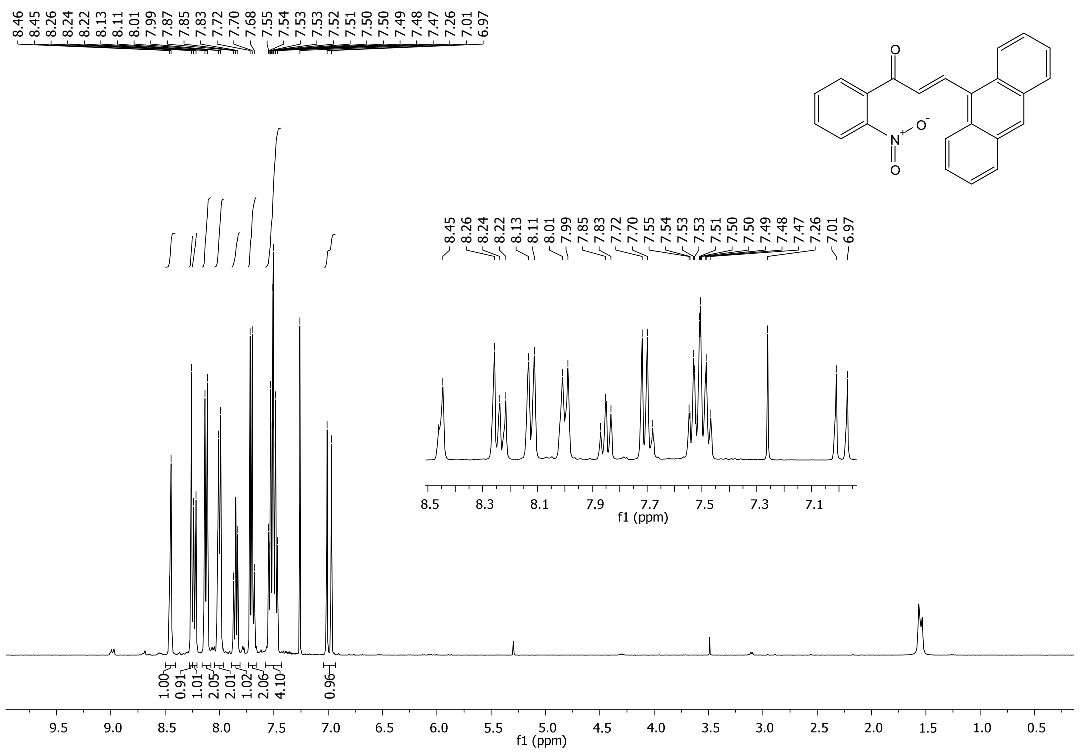

Figure S9. ^{1}H NMR of (*E*)-3-(anthracen-9-yl)-1-(2-nitrophenyl)prop-2-en-1-one (**1m**).

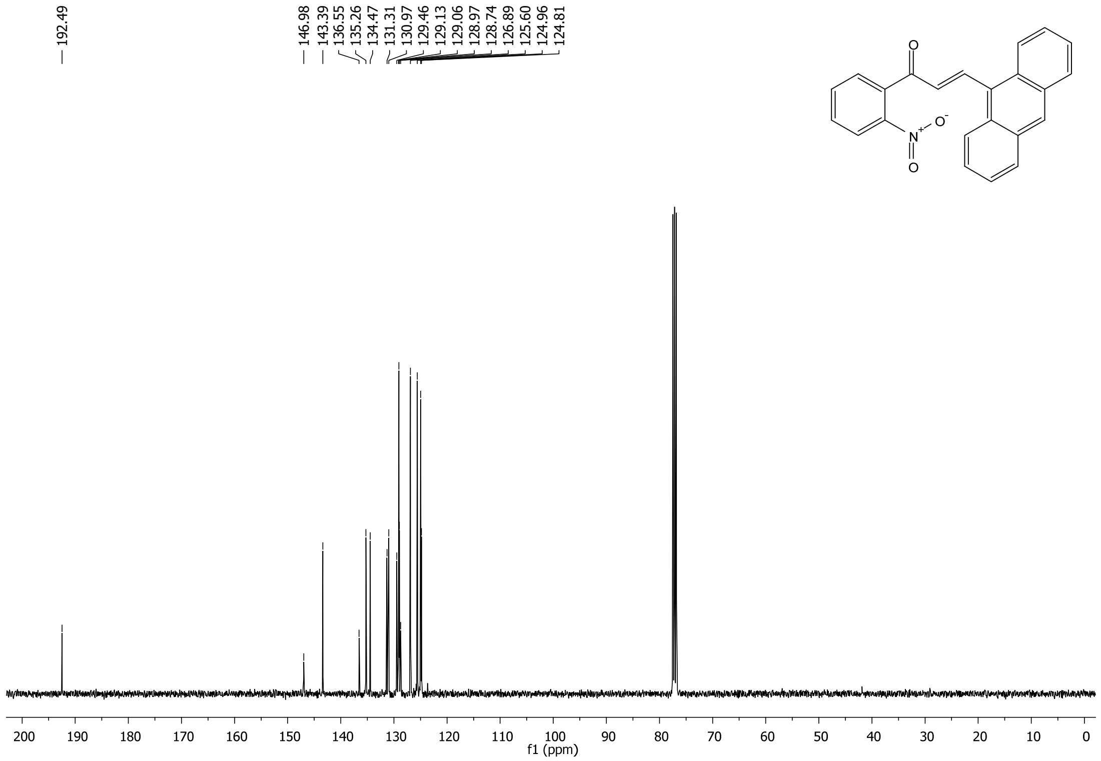

Figure S10. ^{13}C NMR of (*E*)-3-(anthracen-9-yl)-1-(2-nitrophenyl)prop-2-en-1-one (**1m**).

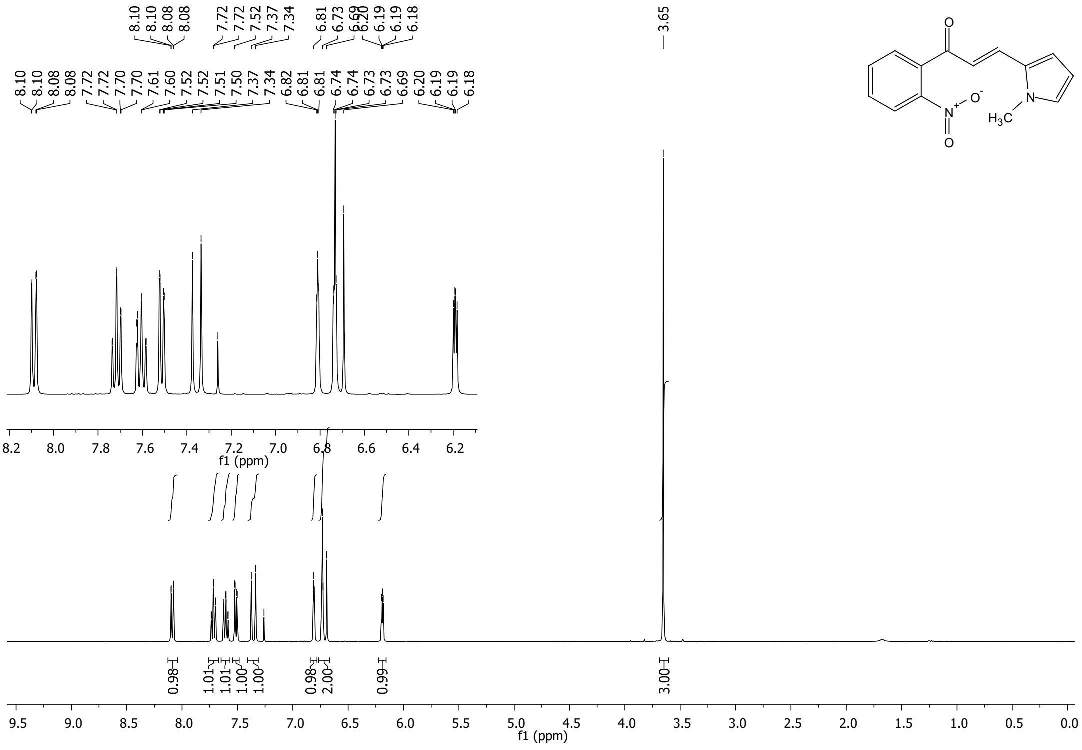

Figure S11. ^{1}H NMR of (*E*)-3-(1-methyl-1H-pyrrol-2-yl)-1-(2-nitrophenyl)prop-2-en-1-one (**1n**).

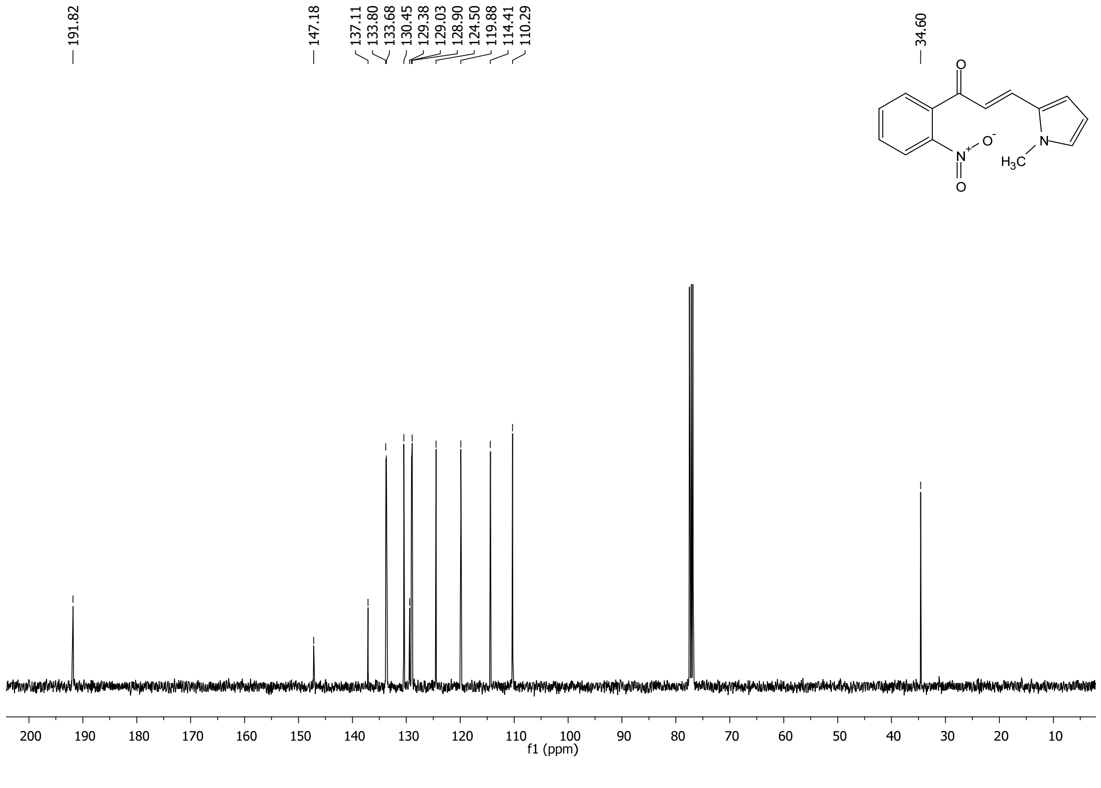

Figure S12. ^{13}C NMR of (*E*)-3-(1-methyl-1H-pyrrol-2-yl)-1-(2-nitrophenyl)prop-2-en-1-one (**1n**).

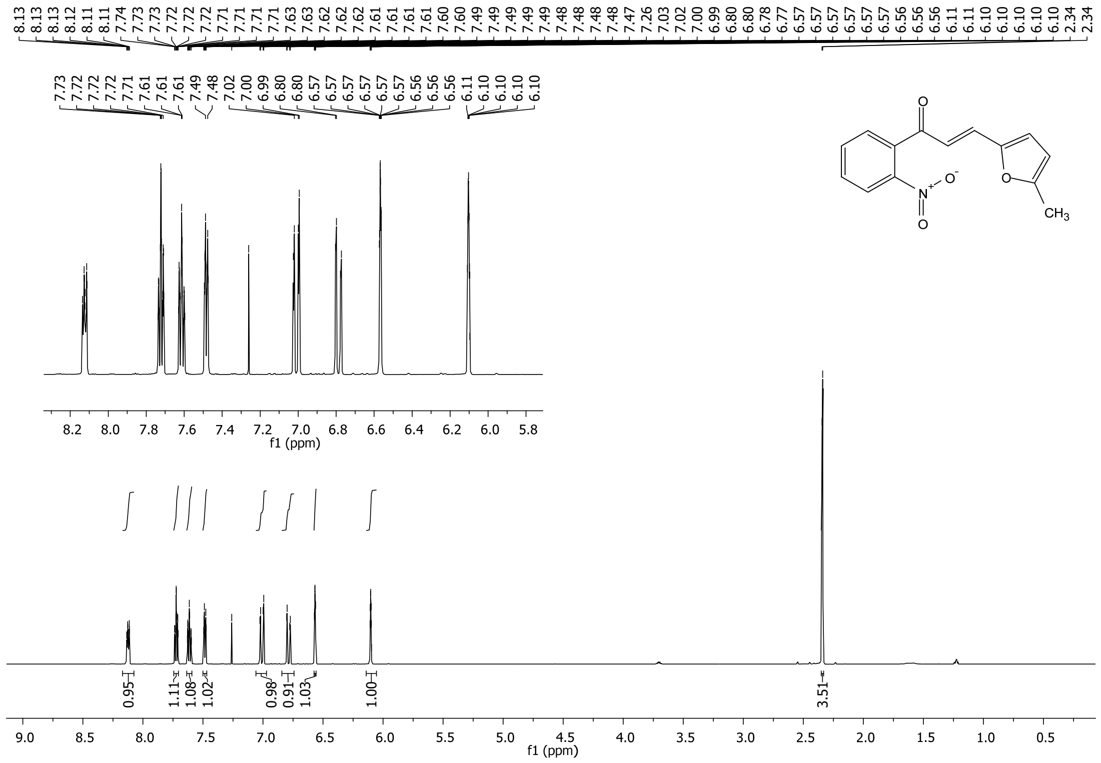

Figure S13. ^{1}H NMR of (*E*)-3-(5-methylfuran-2-yl)-1-(2-nitrophenyl)prop-2-en-1-one (**1o**).

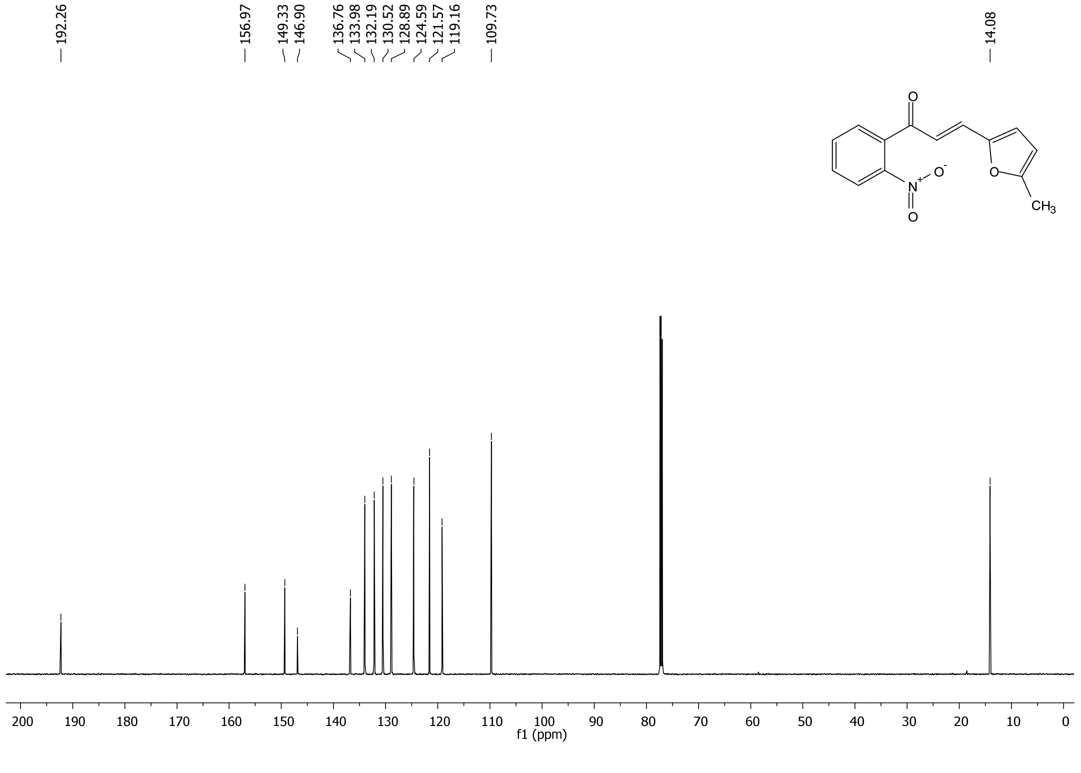

Figure S14. ^{13}C NMR of (*E*)-3-(5-methylfuran-2-yl)-1-(2-nitrophenyl)prop-2-en-1-one (**1o**).

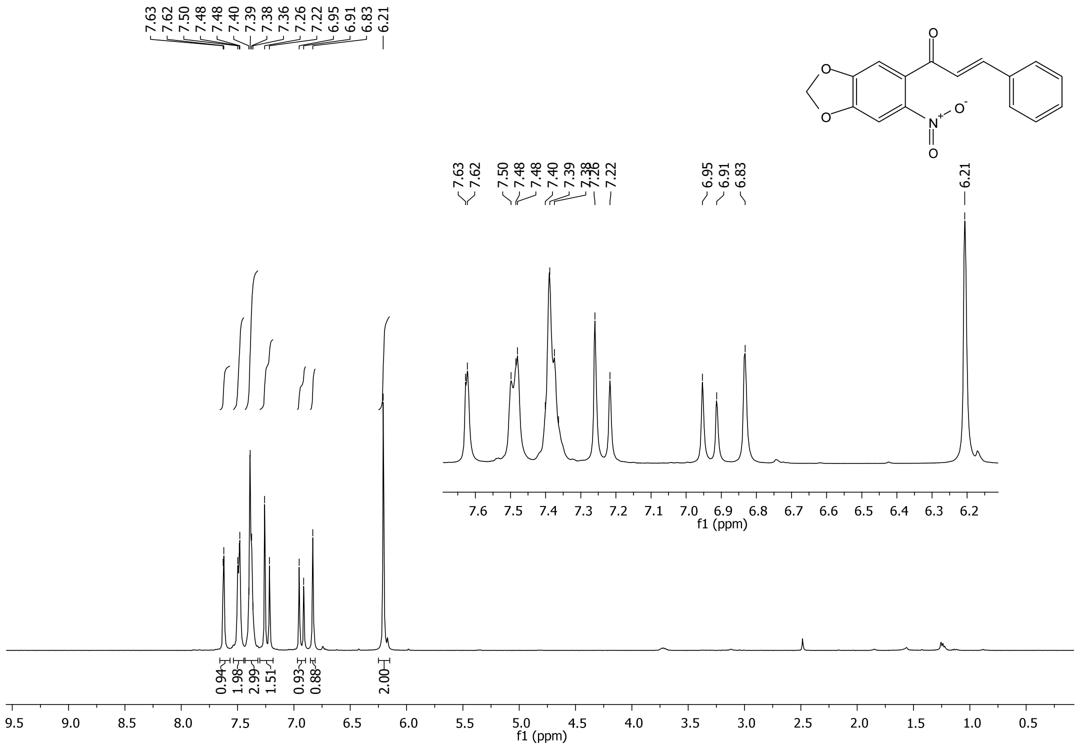

Figure S15. ^{1}H NMR of (*E*)-1-(6-nitrobenzo[d][1,3]dioxol-5-yl)-3-phenylprop-2-en-1-one (**1p**).

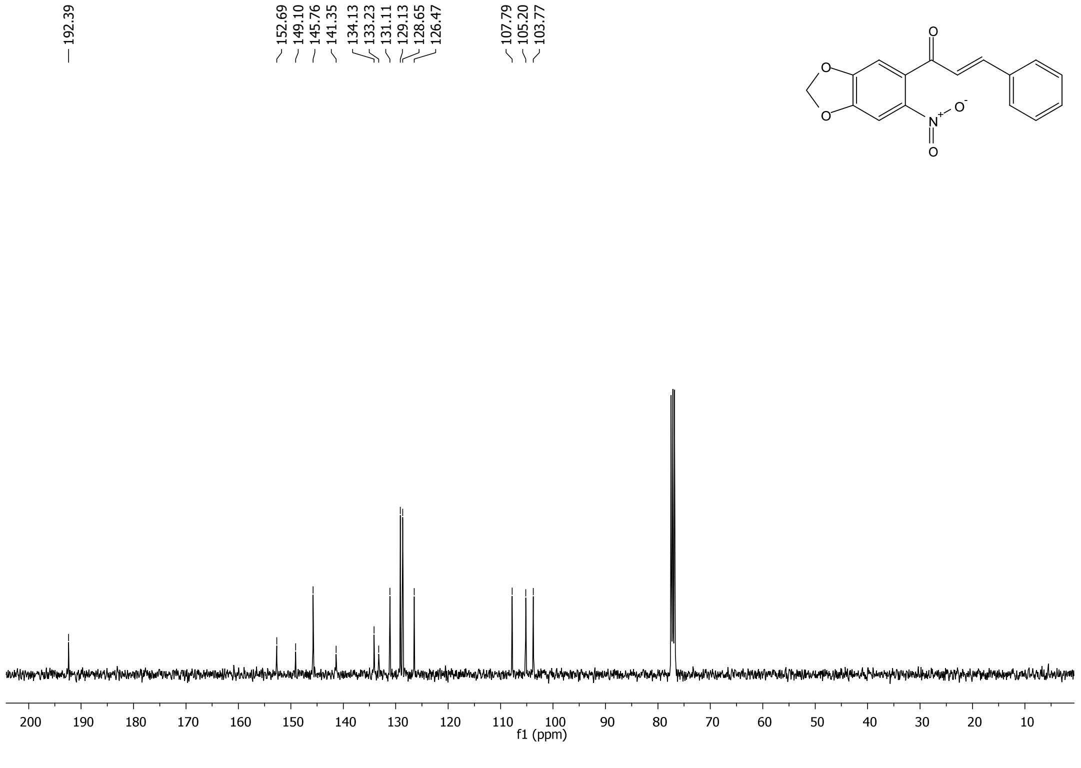

Figure S16. ^{13}C NMR of (*E*)-1-(6-nitrobenzo[d][1,3]dioxol-5-yl)-3-phenylprop-2-en-1-one (**1p**).

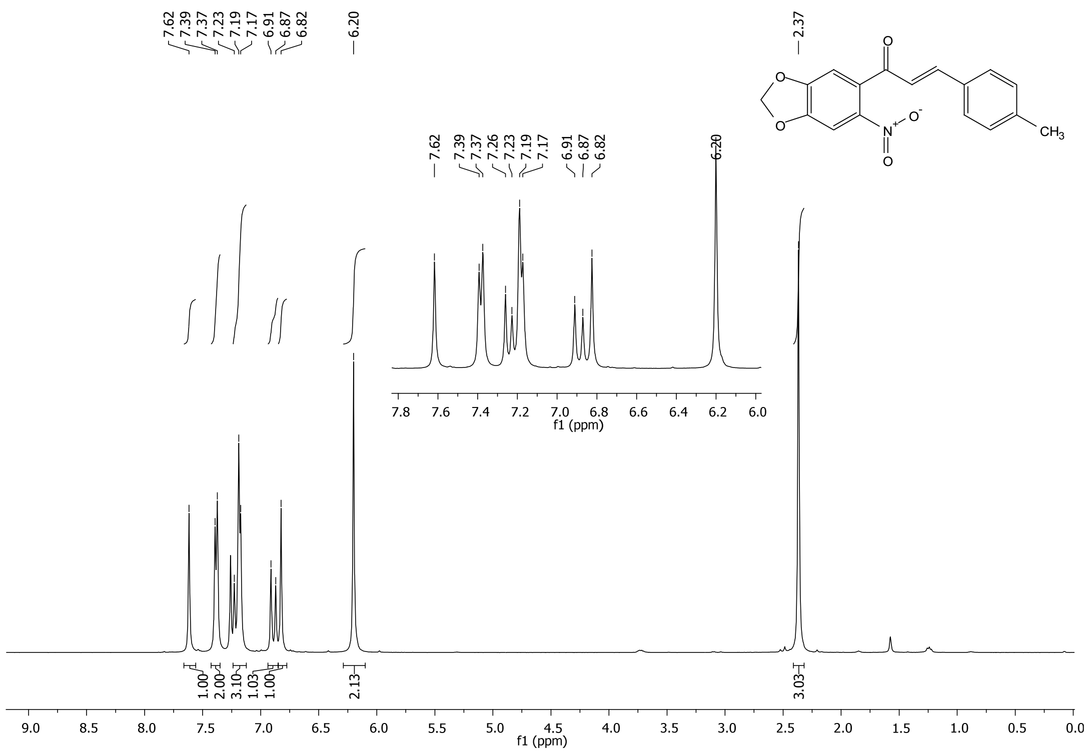

Figure S17. ^{1}H NMR of (*E*)-1-(6-nitrobenzo[d][1,3]dioxol-5-yl)-3-(p-tolyl)prop-2-en-1-one (**1q**).

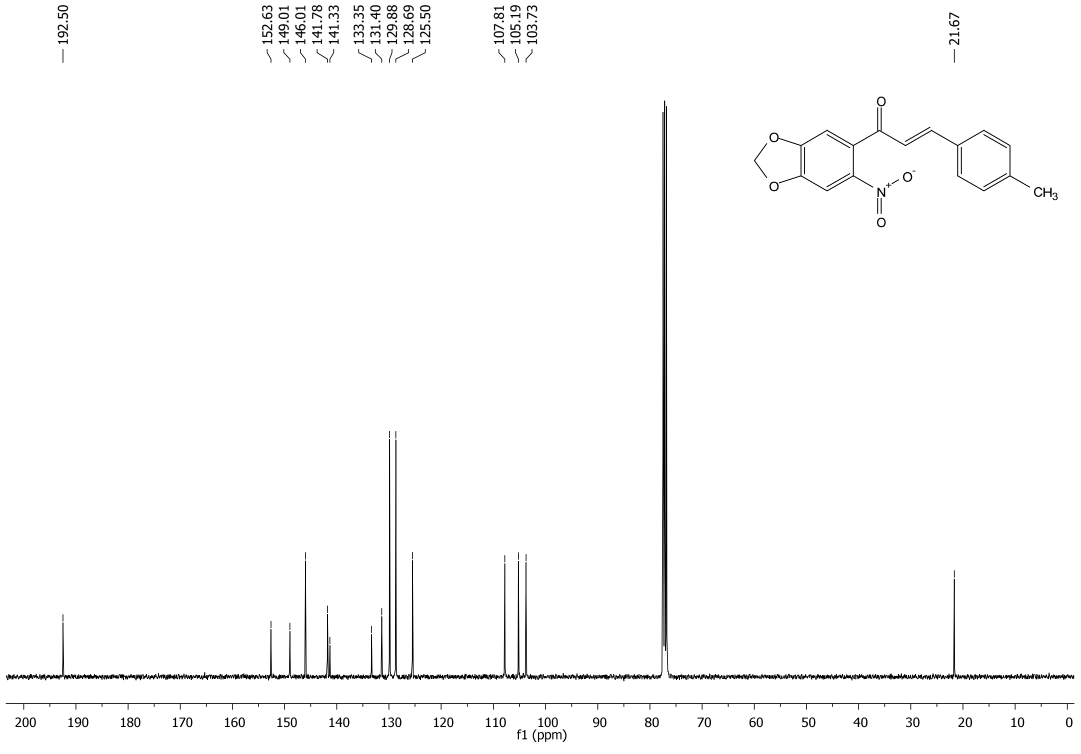

Figure S18. ^{13}C NMR of (*E*)-1-(6-nitrobenzo[d][1,3]dioxol-5-yl)-3-(p-tolyl)prop-2-en-1-one (**1q**).

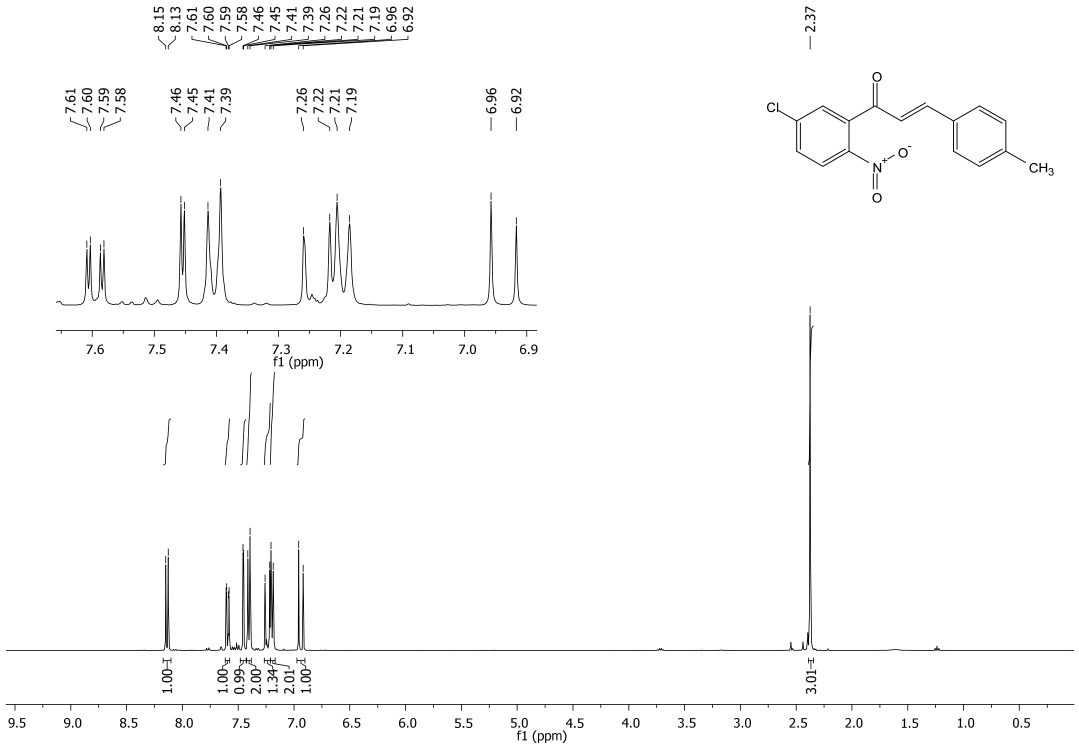

Figure S19. ^{1}H NMR of (*E*)-1-(5-chloro-2-nitrophenyl)-3-(p-tolyl)prop-2-en-1-one (**1r**).

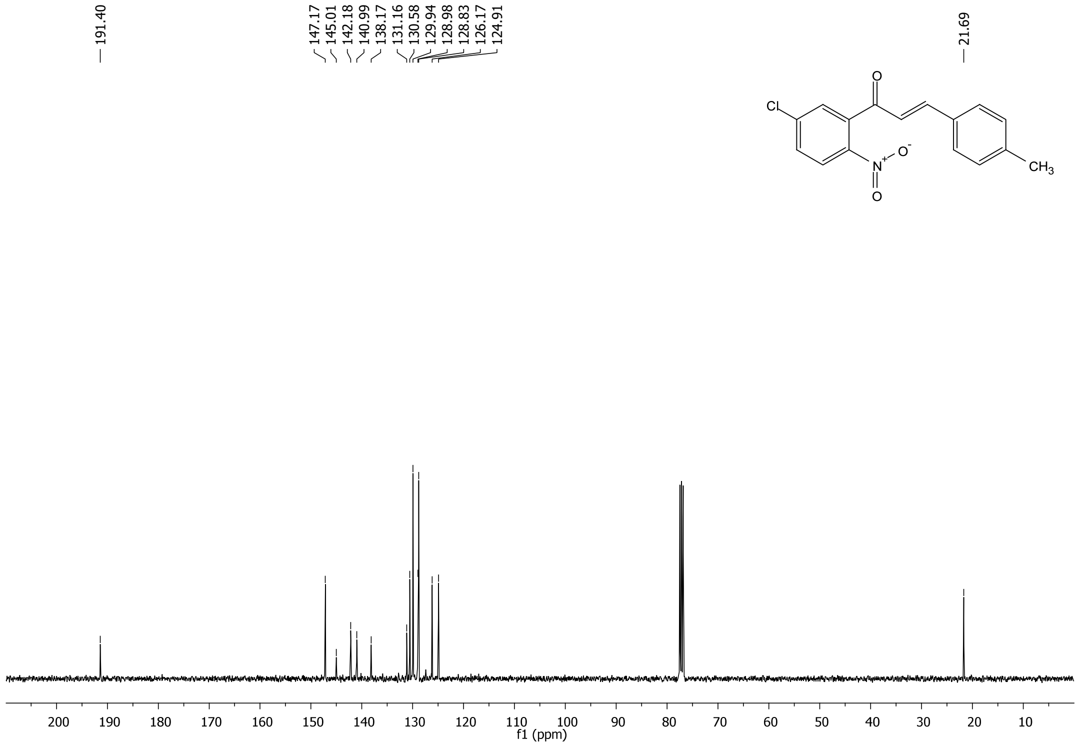

Figure S20. ^{13}C NMR of (*E*)-1-(5-chloro-2-nitrophenyl)-3-(p-tolyl)prop-2-en-1-one (**1r**).

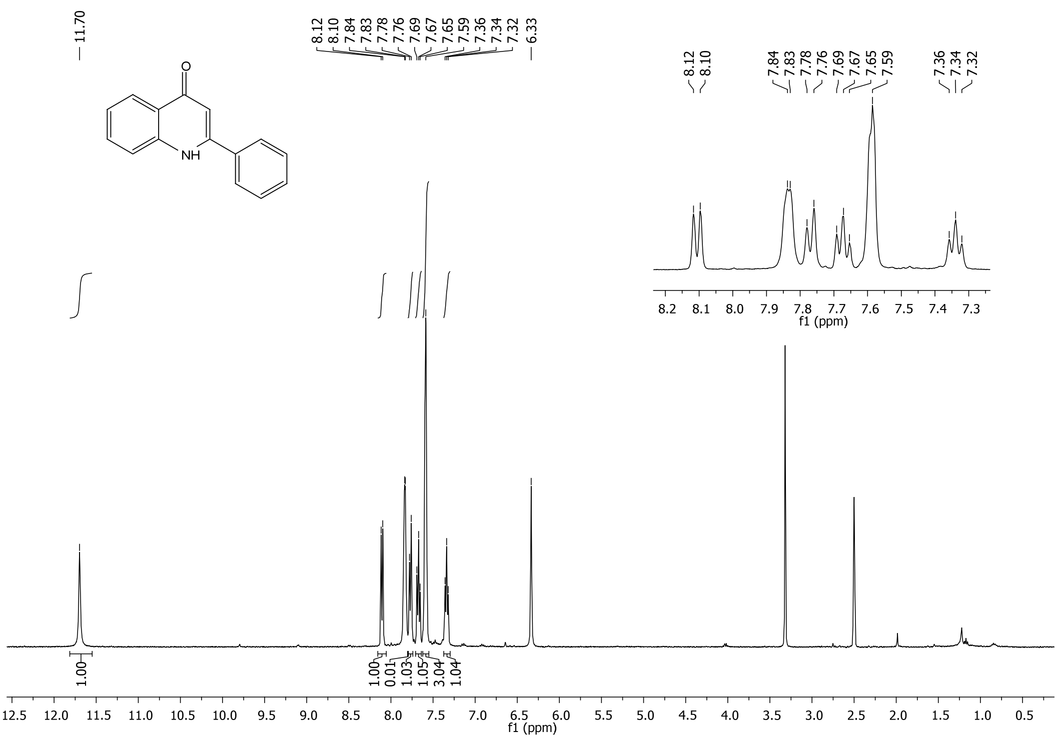

Figure S21. ^{1}H NMR of 2-phenylquinolin-4(1*H*)-one (**2a**)

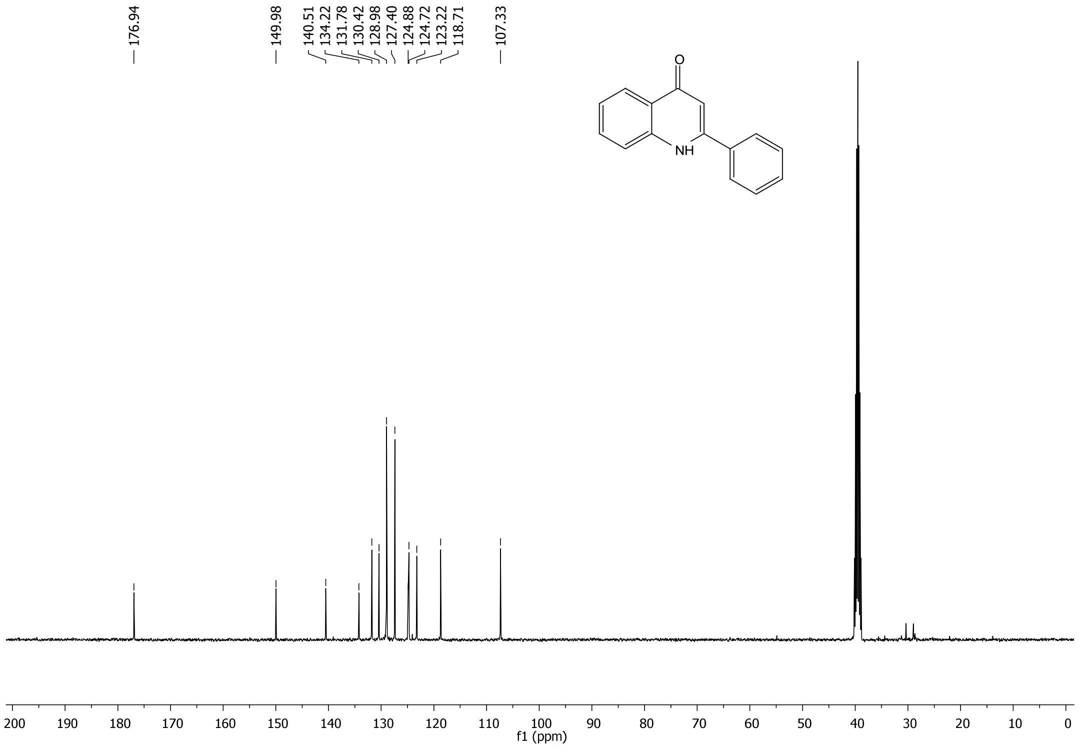

Figure S22. ^{13}C NMR of 2-phenylquinolin-4(1*H*)-one (**2a**).

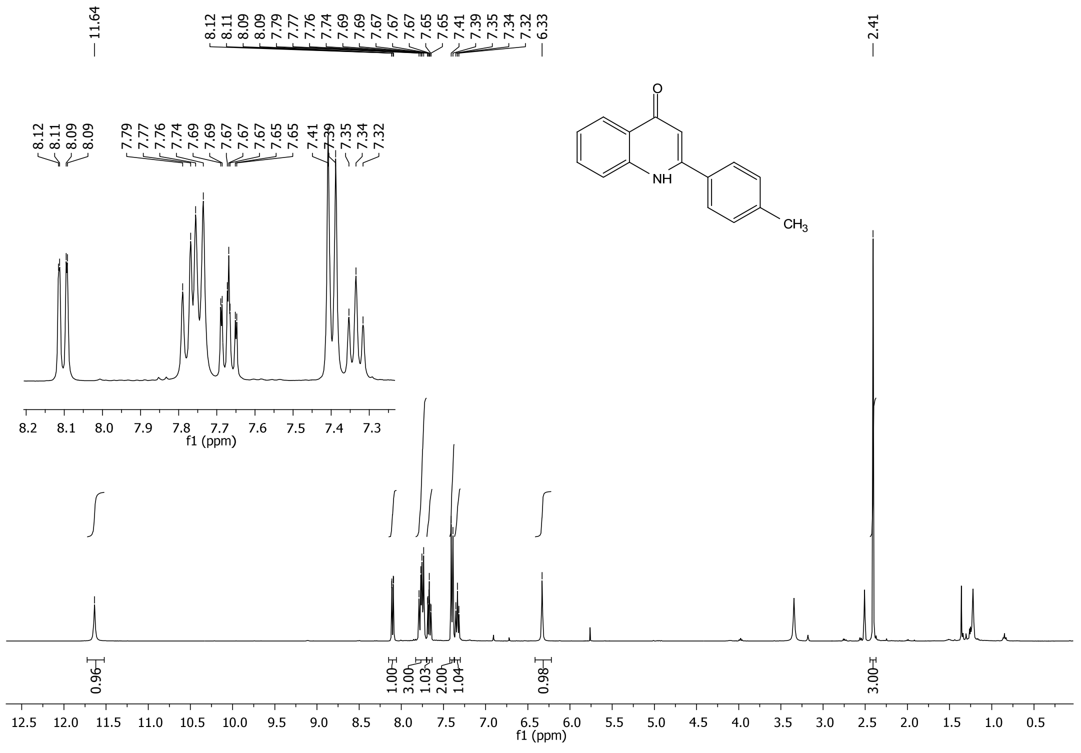

Figure S23. ^{1}H NMR of 2-(4-methylphenyl)quinolin-4(1*H*)-one (**2b**).

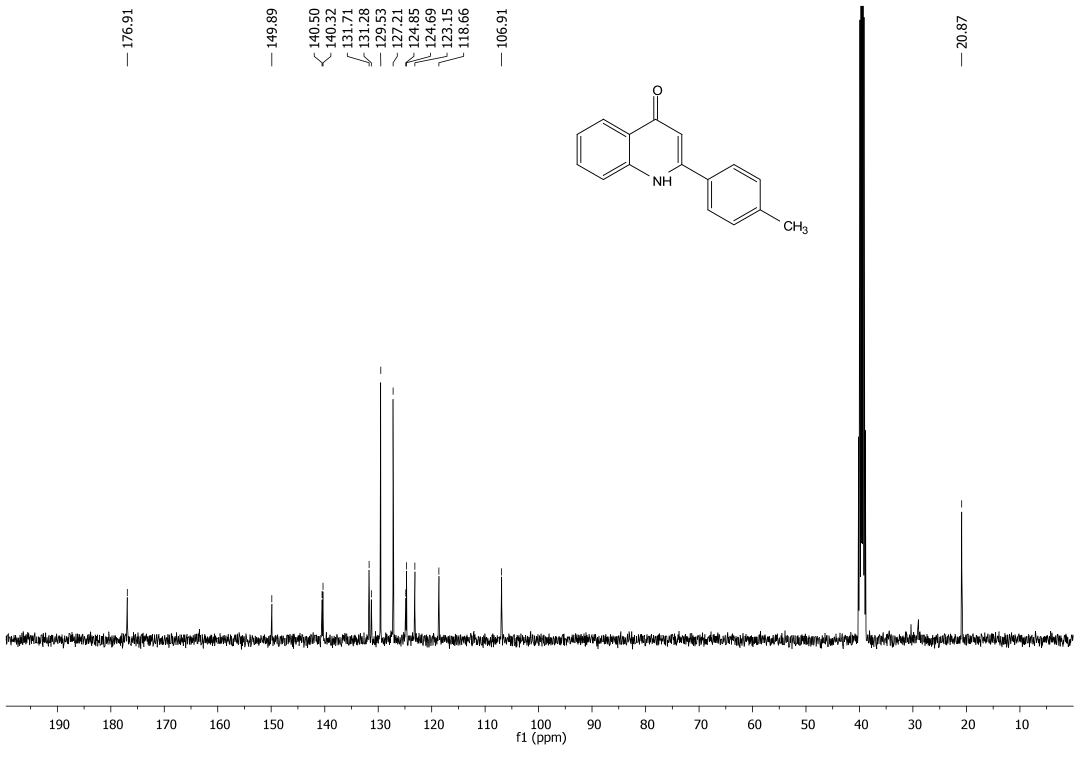

Figure S24. ^{13}C NMR of 2-(4-methylphenyl)quinolin-4(1*H*)-one (**2b**).

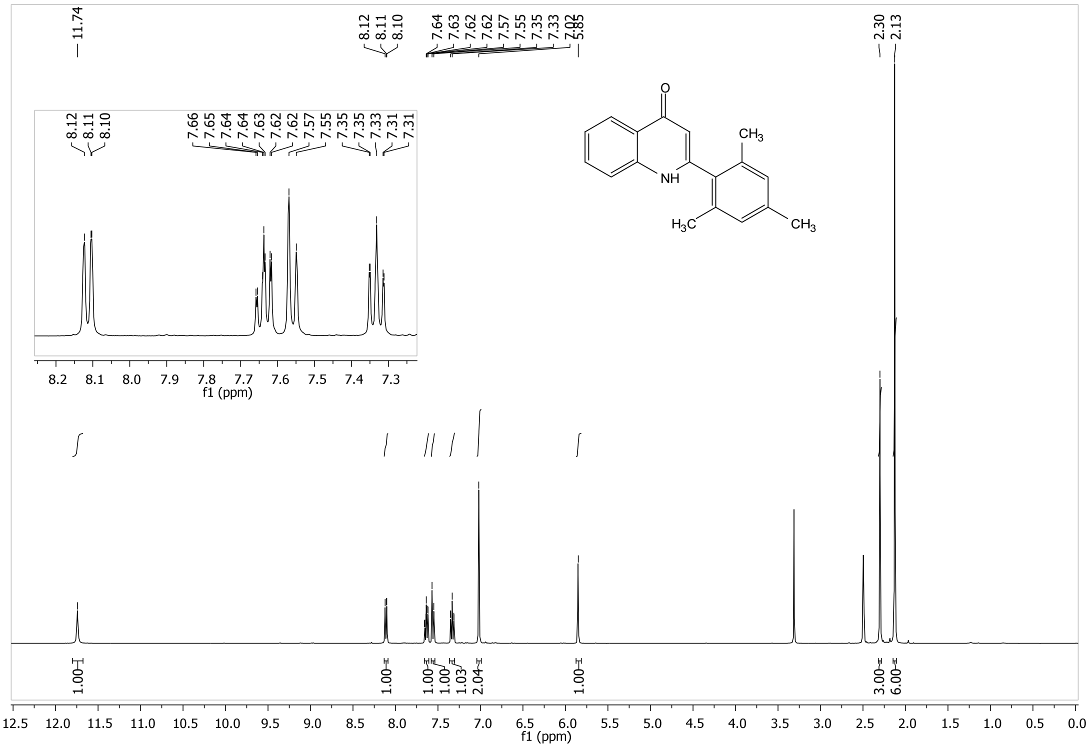

Figure S25. ^{1}H NMR of 2-(2,4,6-trimethylphenyl)quinolin-4(1*H*)-one (**2c**).

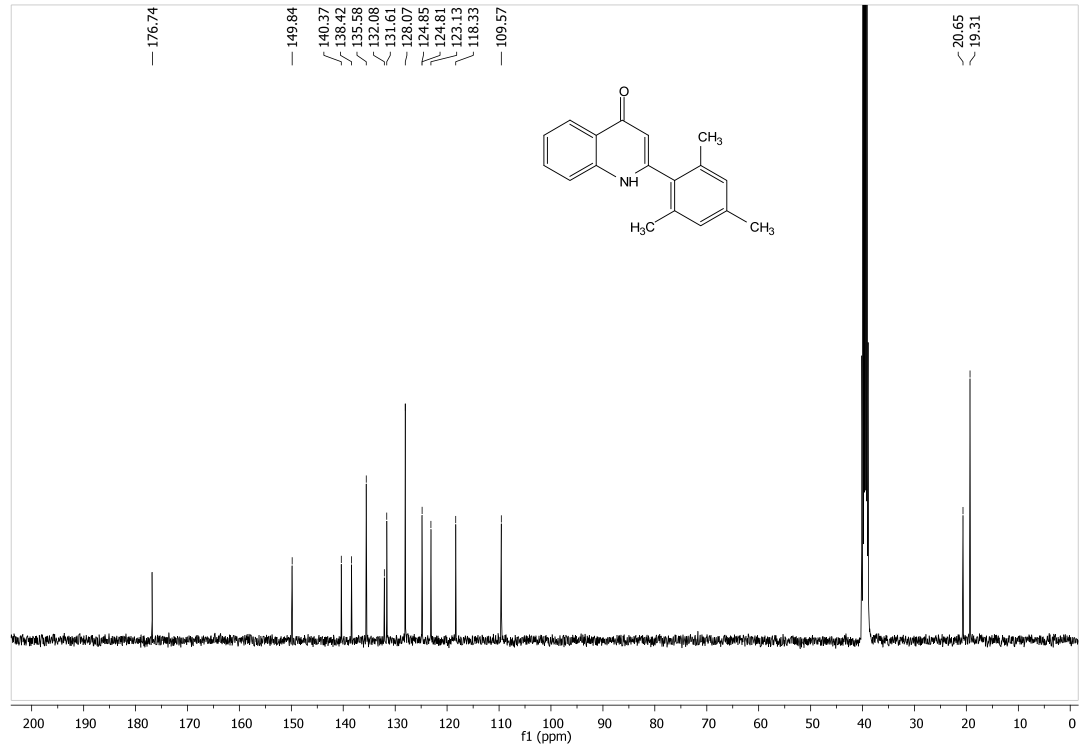

Figure S26. ^{13}C NMR of 2-(2,4,6-trimethylphenyl)quinolin-4(1*H*)-one (**2c**).

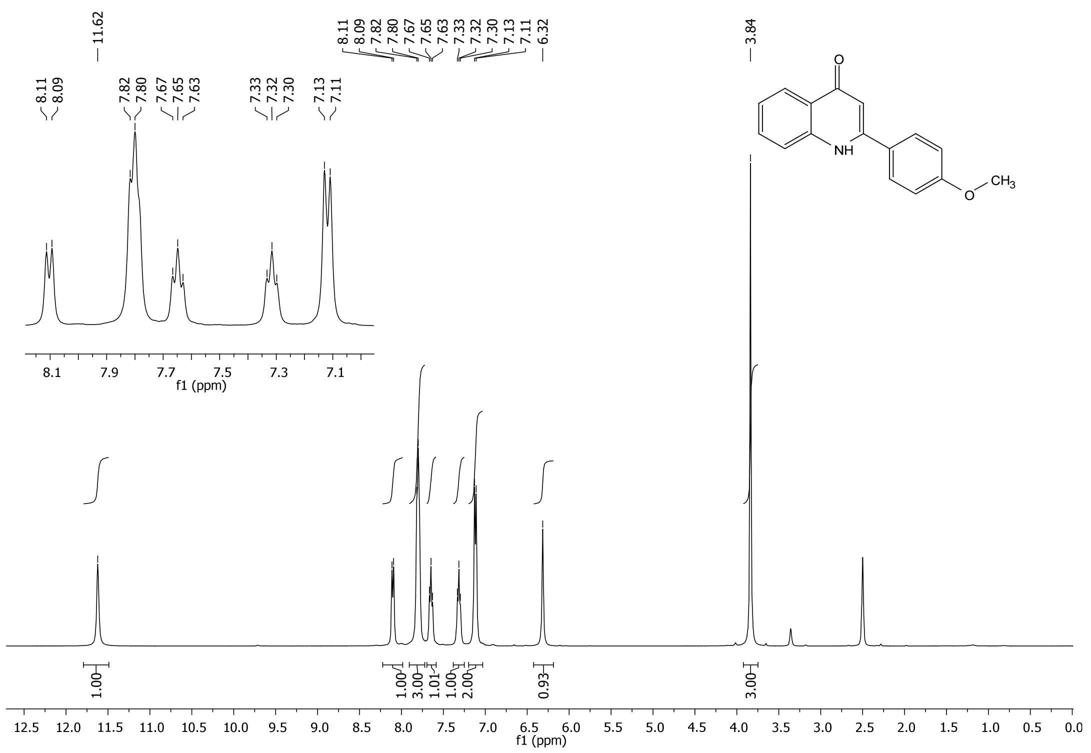

Figure S27. ^{1}H NMR of 2-(4-methoxyphenyl)quinolin-4(1*H*)-one (**2d**).

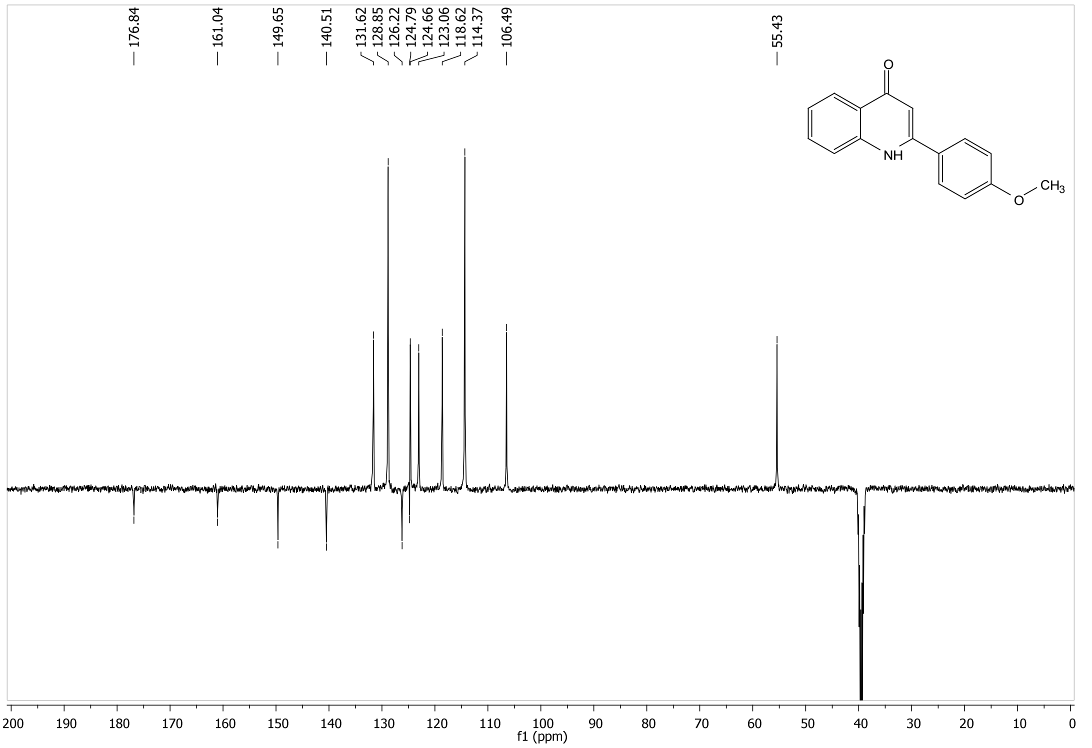

Figure S28. ^{13}C APT NMR of 2-(4-methoxyphenyl)quinolin-4(1*H*)-one (**2d**).

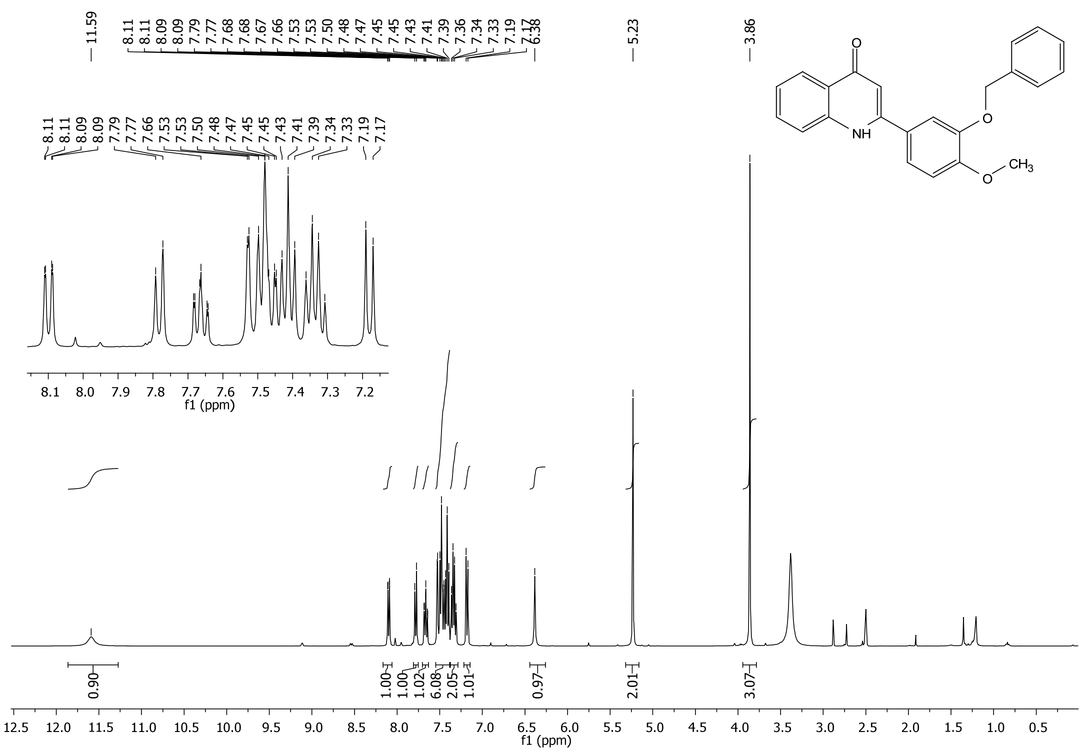

Figure S29. ^{1}H NMR of 2-(*p*-tolyl)quinolin-4(1*H*)-one (**2e**).

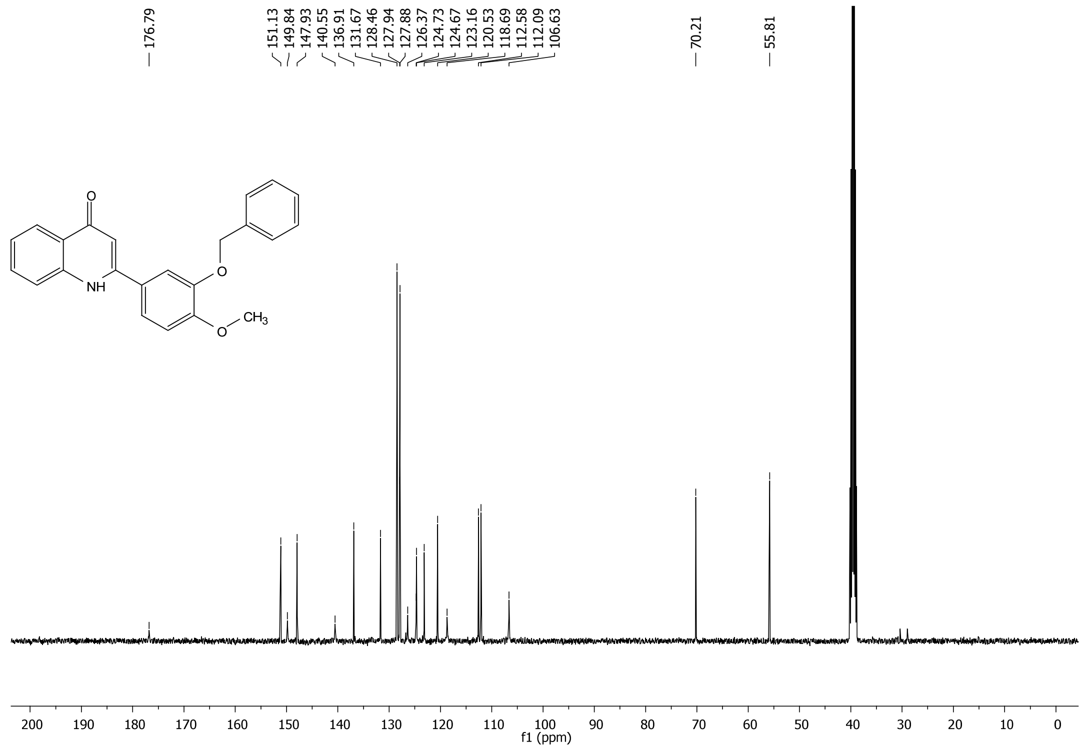

Figure S30. ^{13}C NMR of 2-(3-benzyloxy-4-methoxy-phenyl)quinolin-4(1*H*)-one (**2e**).

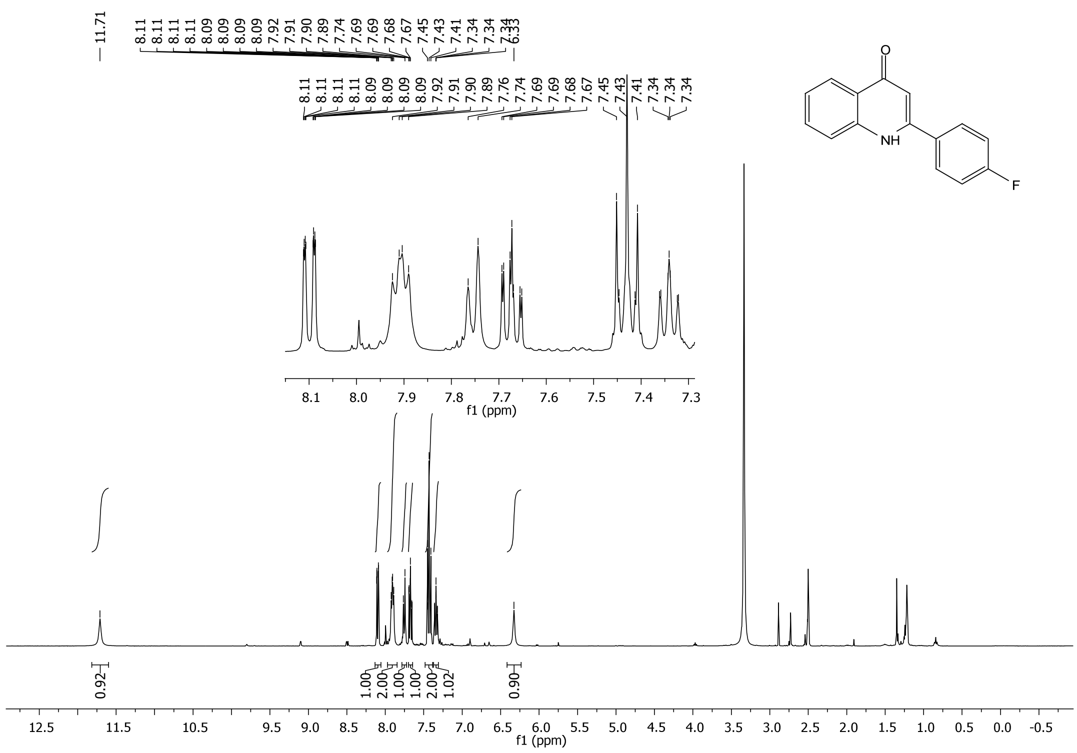

Figure S31. ^{1}H NMR of 2-(4-fluorophenyl)quinolin-4(1*H*)-one (**2g**).

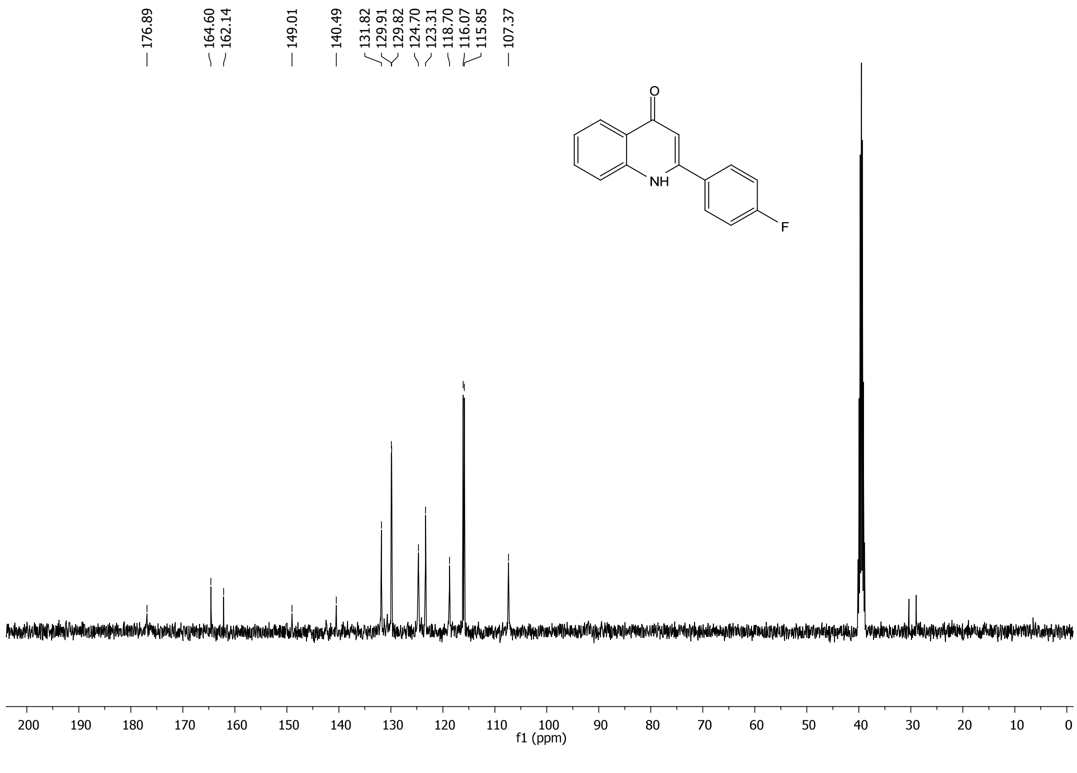

Figure S32. ^{13}C NMR of 2-(4-fluorophenyl)quinolin-4(1*H*)-one (**2g**).

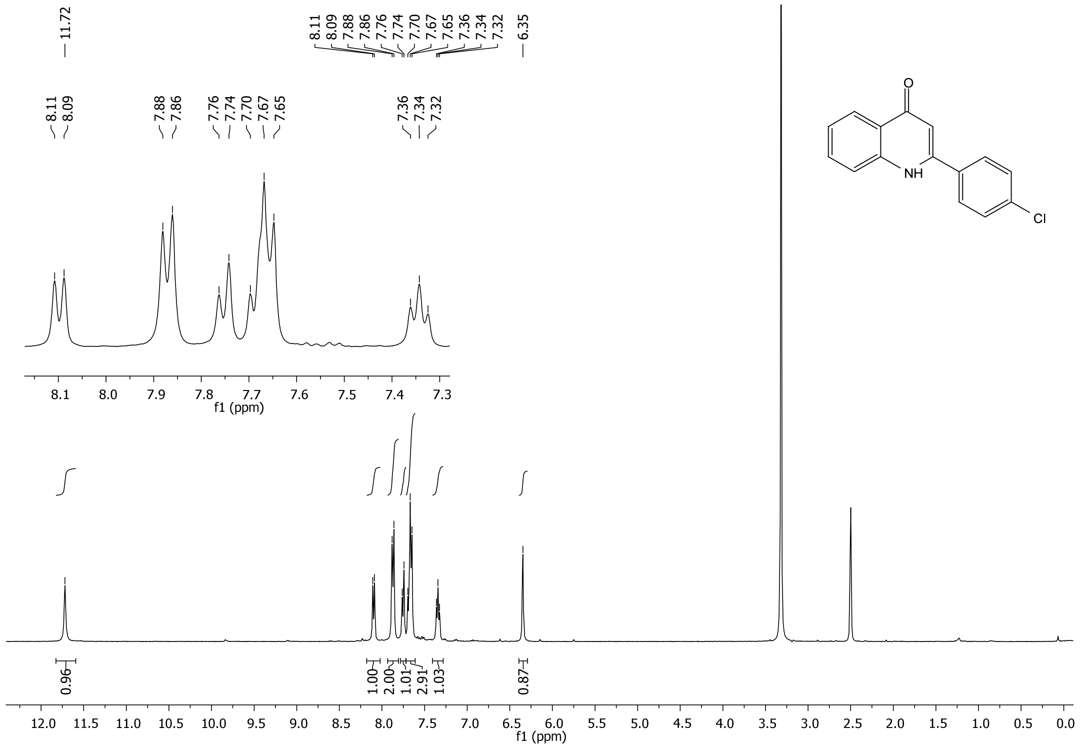

Figure S33. ^{1}H NMR of 2-(4-chlorophenyl)quinolin-4(1*H*)-one (**2h**).

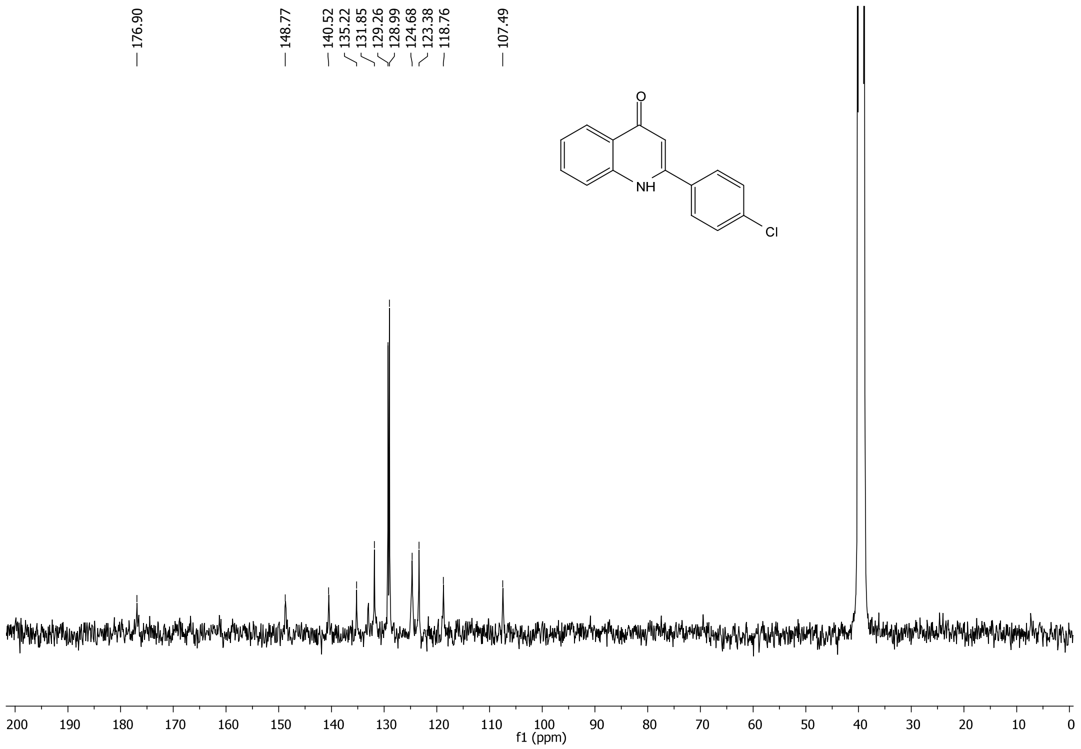

Figure S34. ^{13}C NMR of 2-(4-chlorophenyl)quinolin-4(1*H*)-one (**2h**).

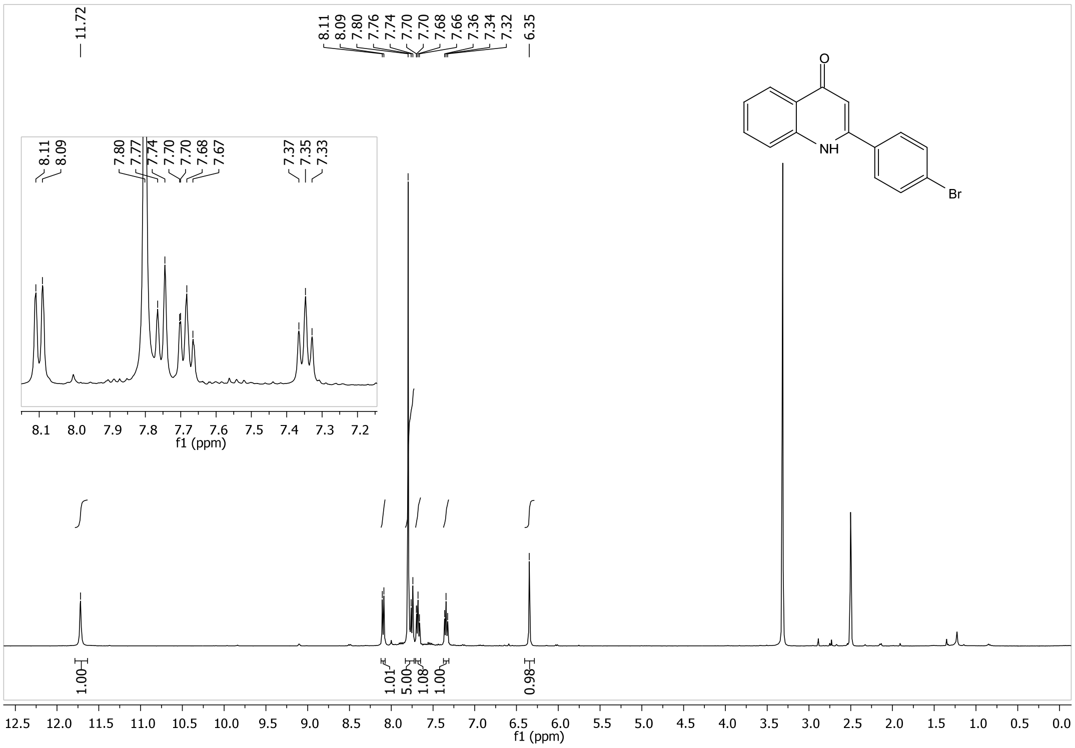

Figure S35. ^{1}H NMR of 2-(4-bromophenyl)quinolin-4(1*H*)-one (**2i**).

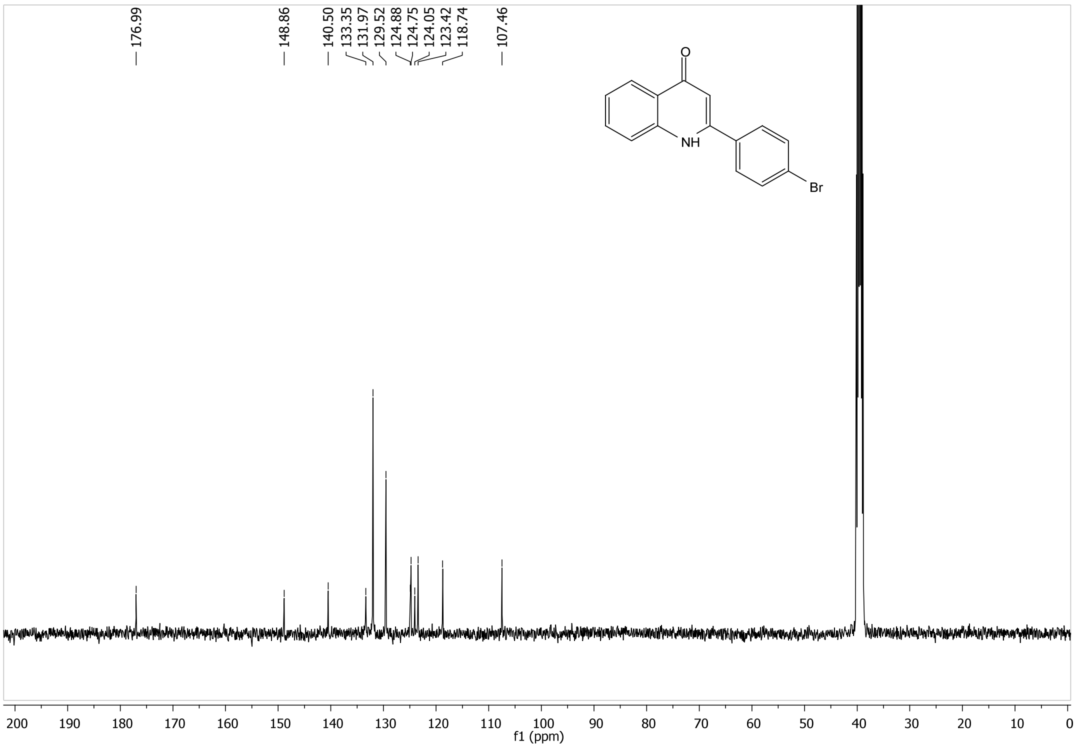

Figure S36. ^{13}C NMR of 2-(4-bromophenyl)quinolin-4(1*H*)-one (**2i**).

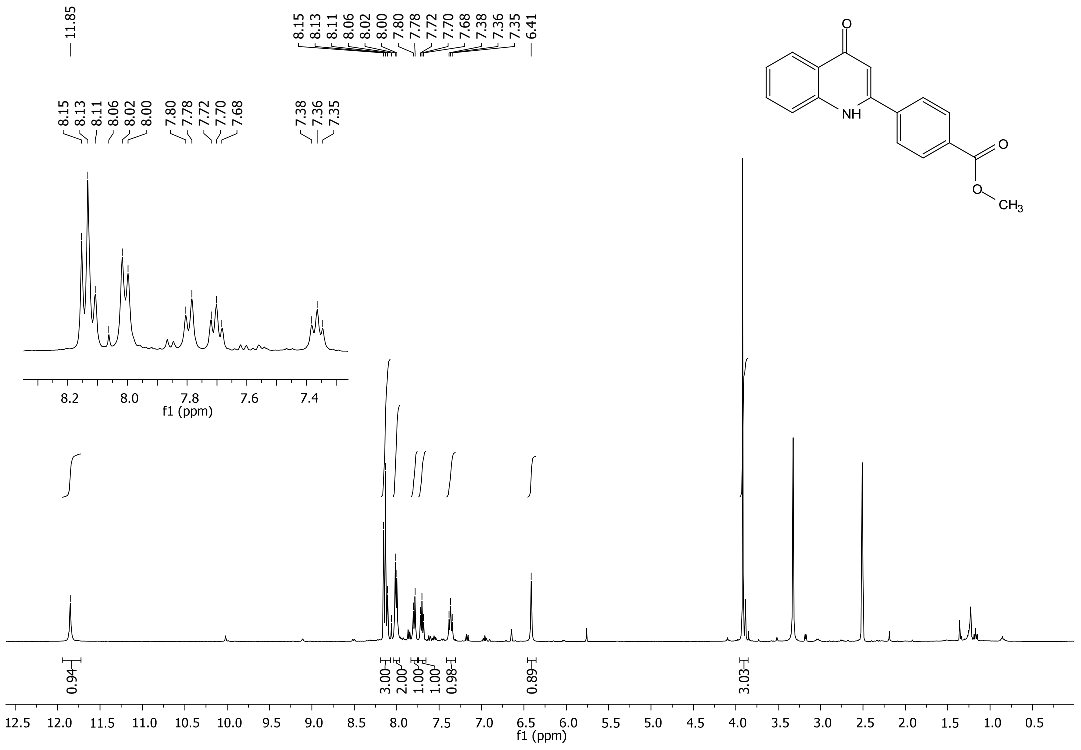

Figure S37. ^{1}H NMR of 2-(4-carbomethoxyphenyl)quinolin-4(1*H*)-one (**2j**).

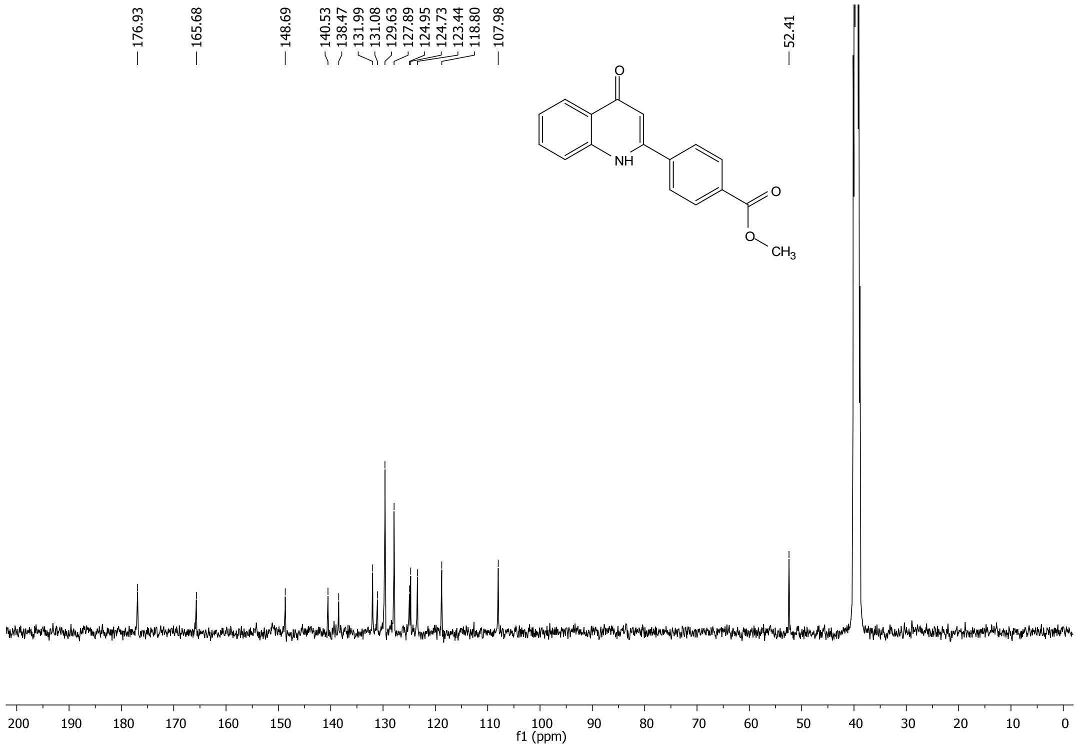

Figure S38. ^{13}C NMR of 2-(4-carbomethoxyphenyl)quinolin-4(1*H*)-one (**2j**).

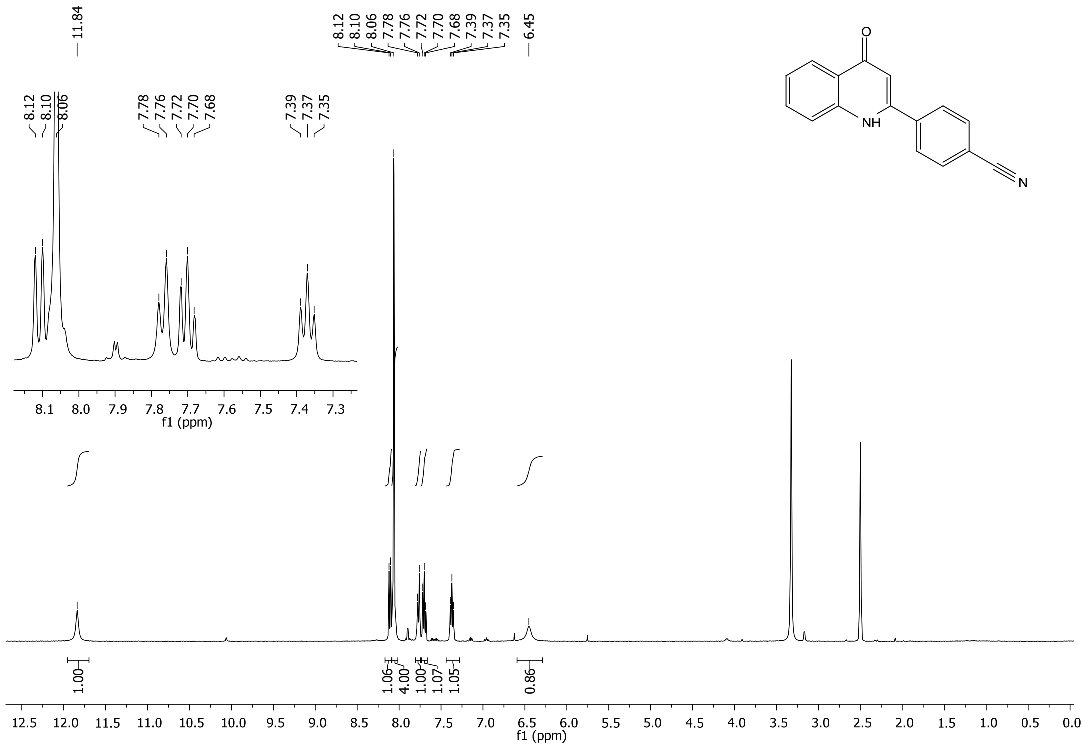

Figure S39. ^{1}H NMR of 2-(4-cyanophenyl)quinolin-4(1*H*)-one (**2k**).

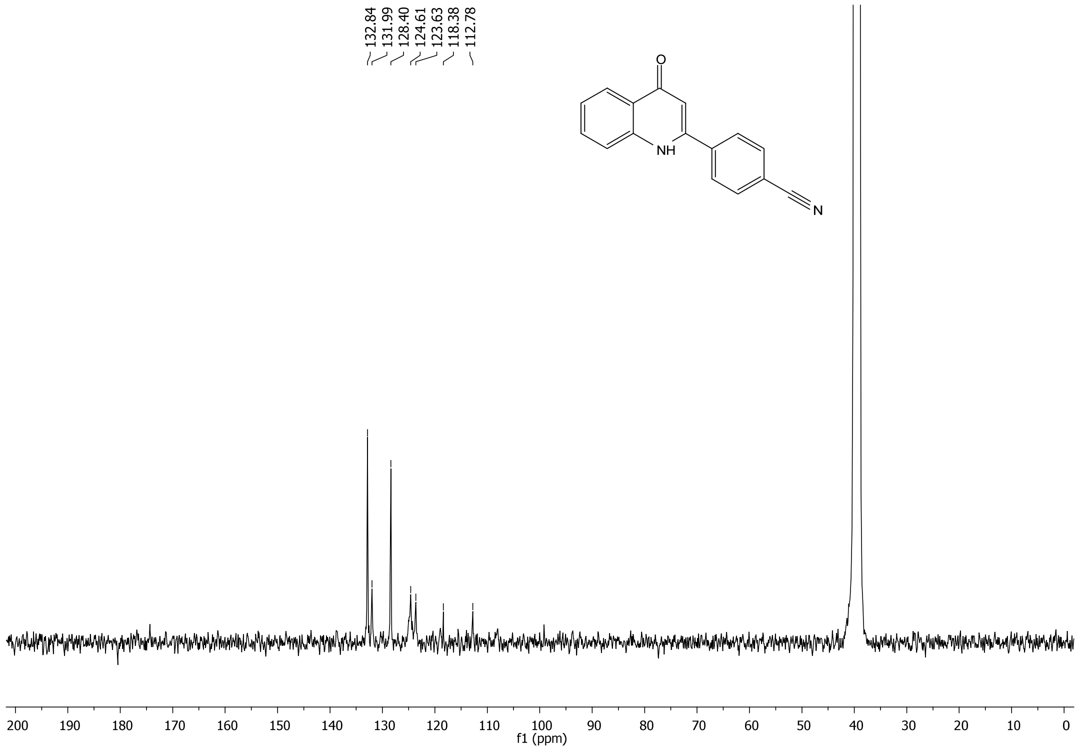

Figure S40. ^{13}C NMR of 2-(4-cyanophenyl)quinolin-4(1*H*)-one (**2k**).

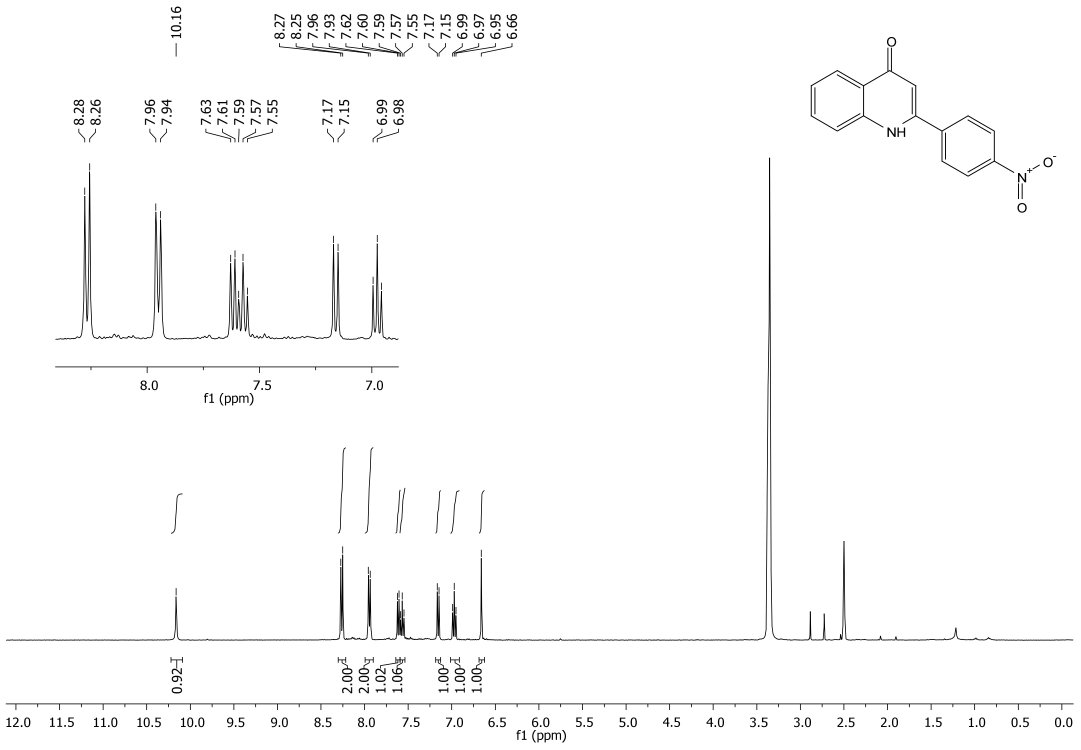

Figure S41. ^{1}H NMR of 2-(4-nitrophenyl)quinolin-4(1*H*)-one (**2l**).

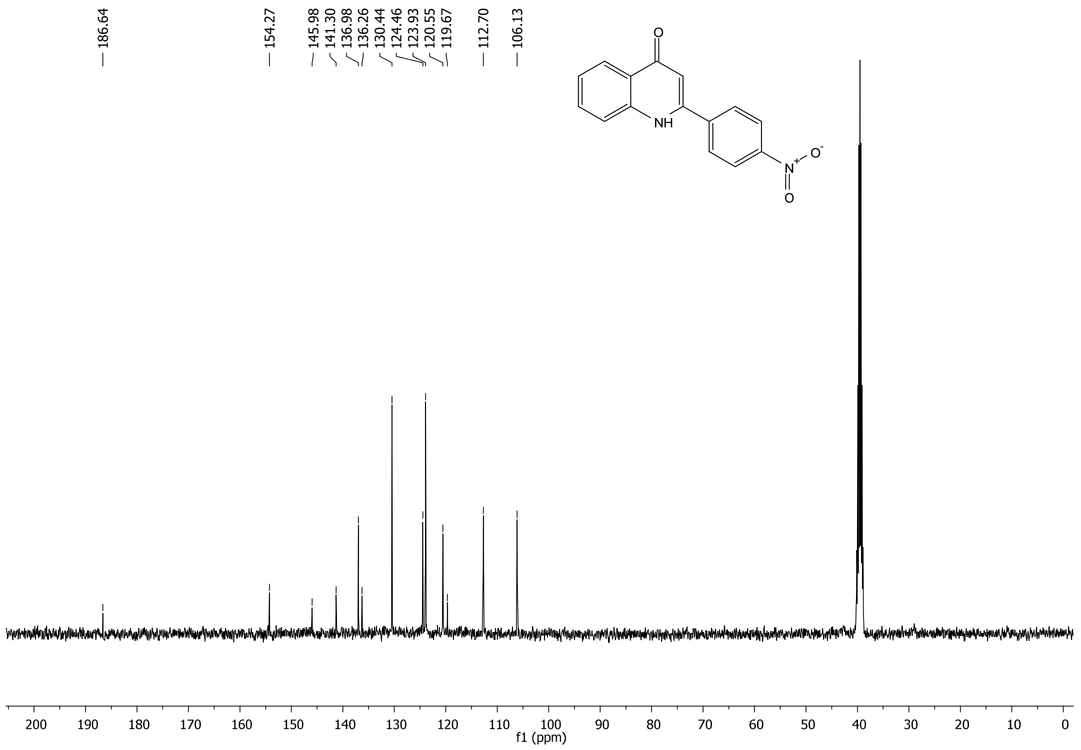

Figure S42. ^{13}C NMR of 2-(4-nitrophenyl)quinolin-4(1*H*)-one (**2l**).

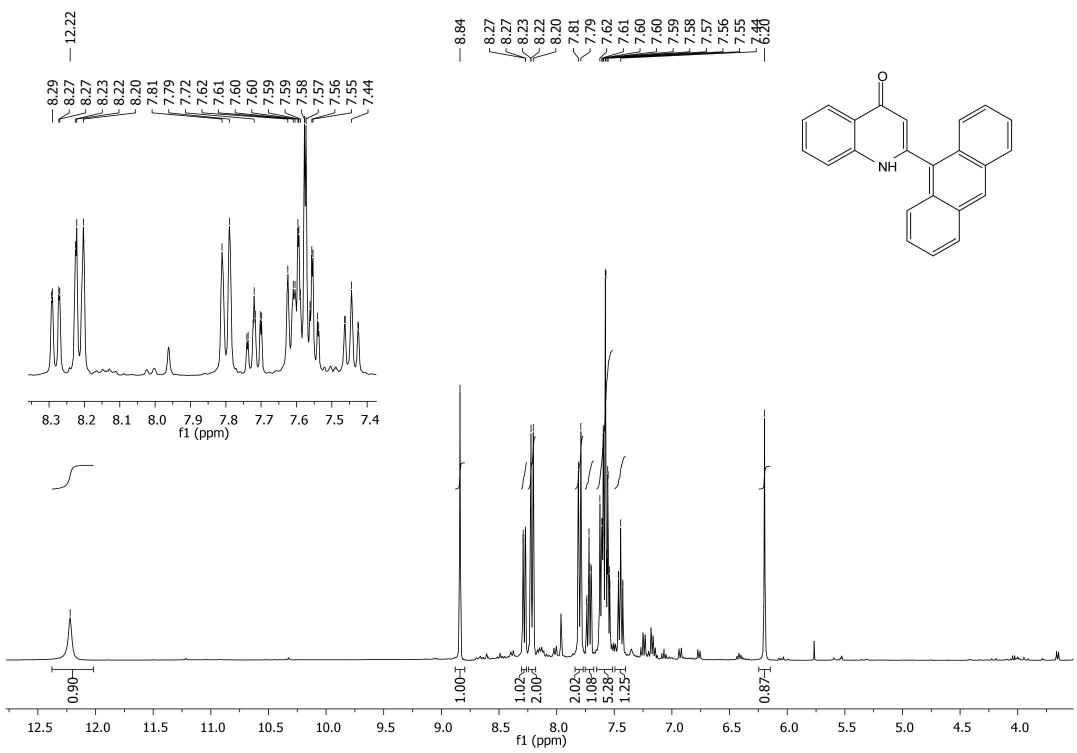

Figure S43. ^{1}H NMR of 2-(anthracen-9-yl)quinolin-4(1*H*)-one (**2m**).

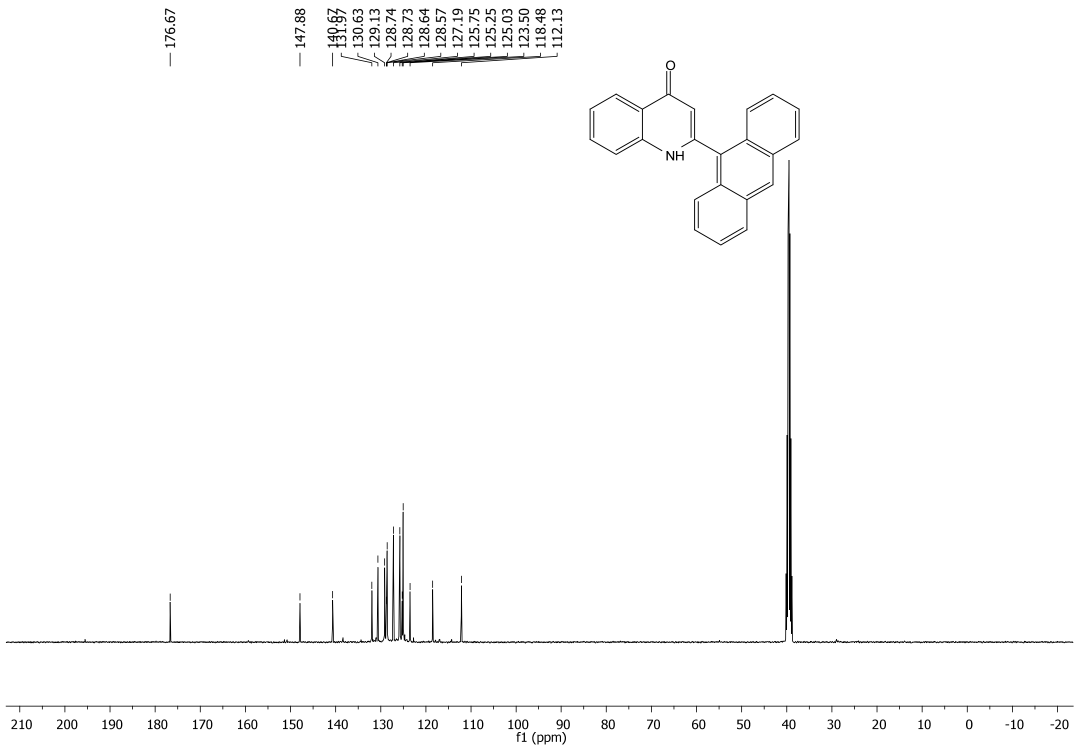

Figure S44. ^{13}C NMR of 2-(anthracen-9-yl)quinolin-4(1*H*)-one (**2m**).

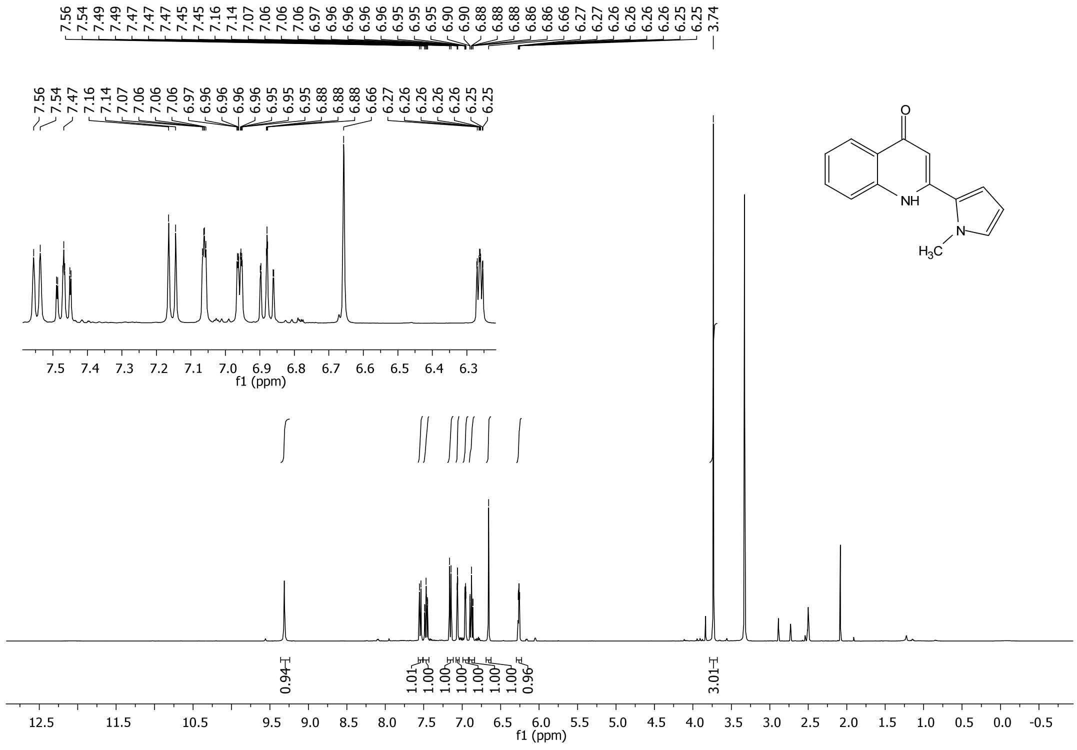

Figure S45. ^{1}H NMR of 2-(1-methyl-1*H*-pyrrol-2-yl)quinolin-4(1*H*)-one (**2n**).

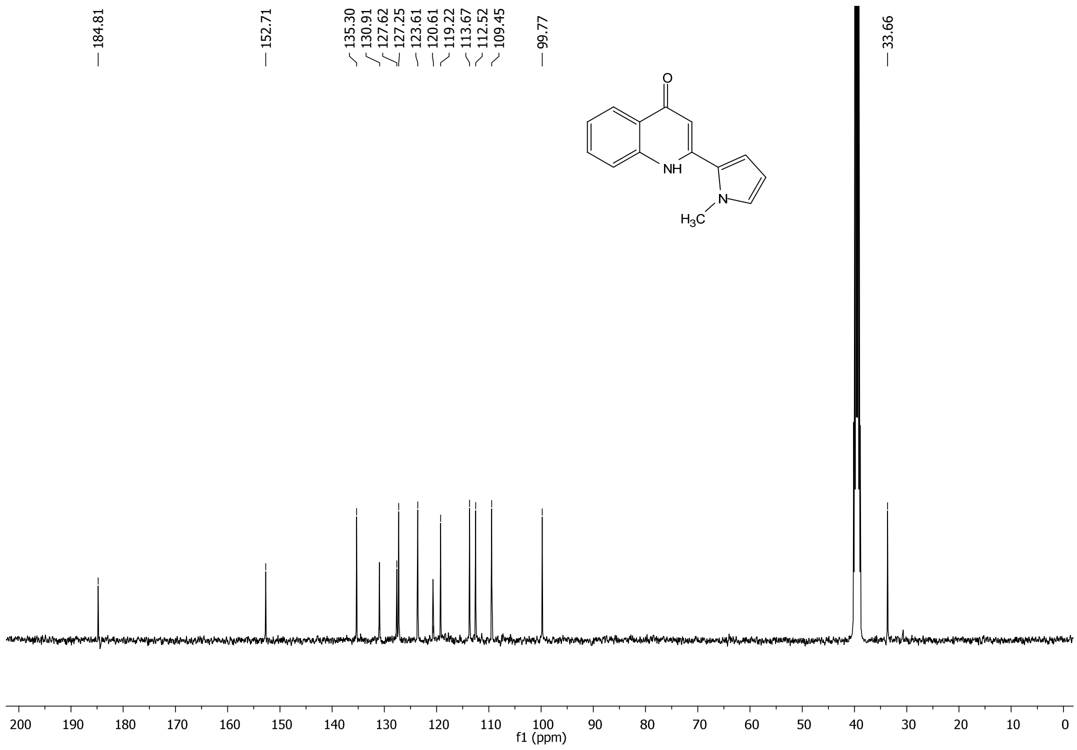

Figure S46. ^{13}C NMR of 2-(1-methyl-1*H*-pyrrol-2-yl)quinolin-4(1*H*)-one (**2n**).

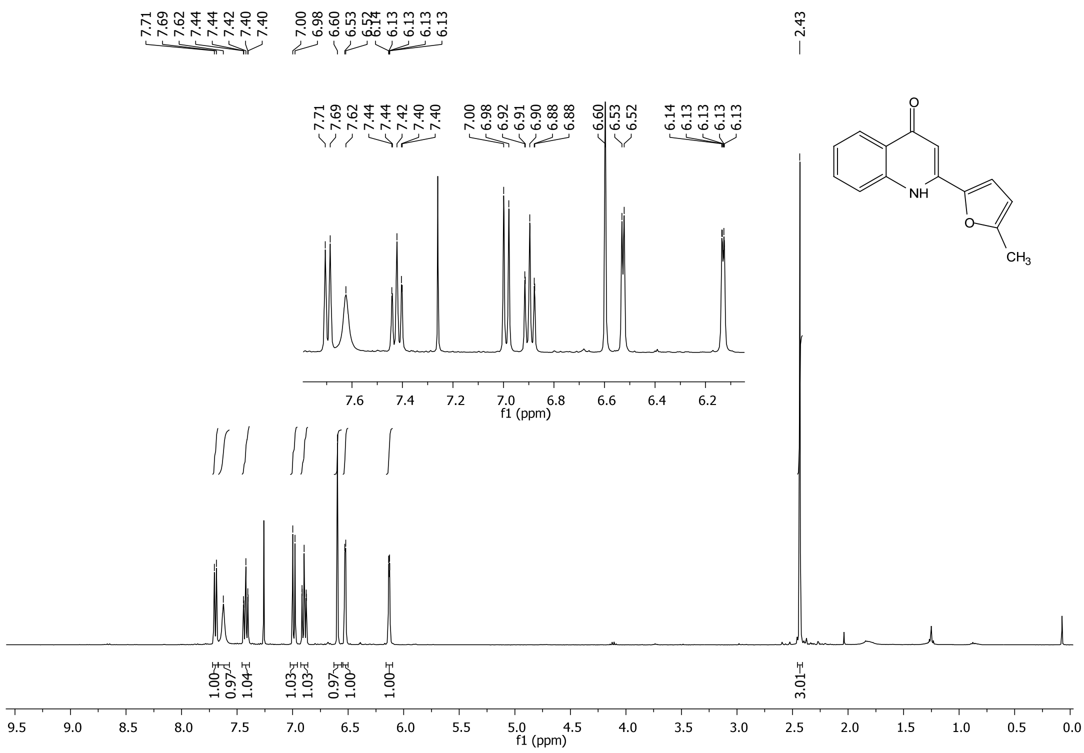

Figure S47. ^{1}H NMR of 2-(5-methylfuran-2-yl)quinolin-4(1*H*)-one (**2o**).

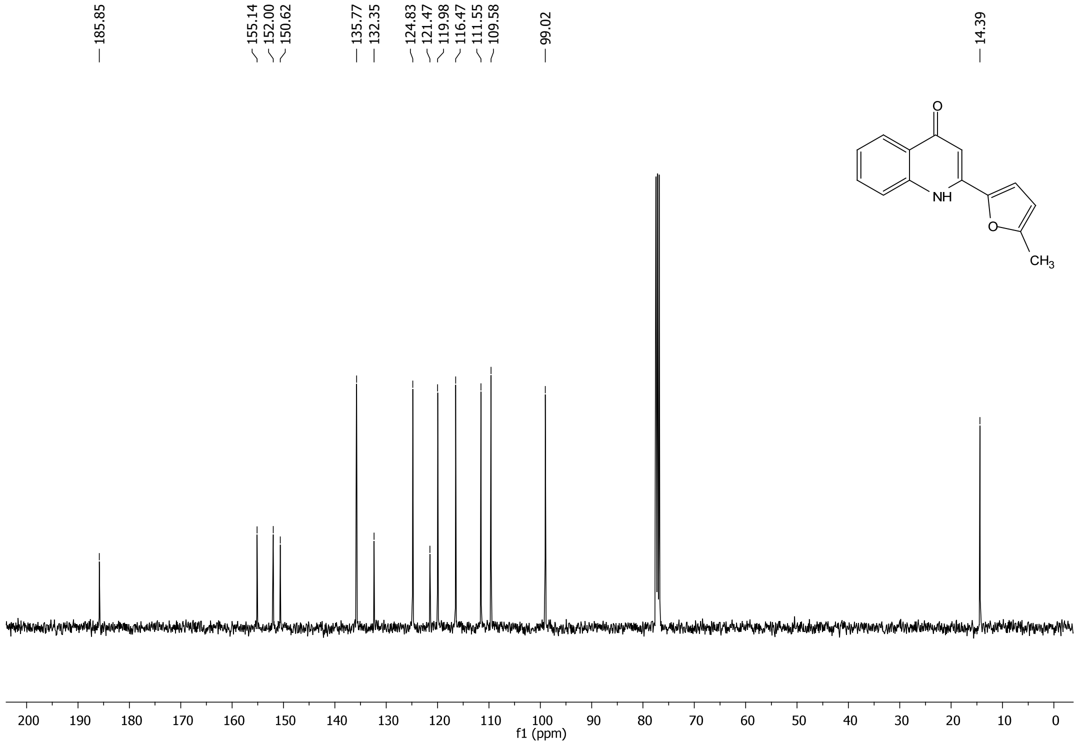

Figure S48. ^{13}C NMR of 2-(5-methylfuran-2-yl)quinolin-4(1*H*)-one (**2o**).

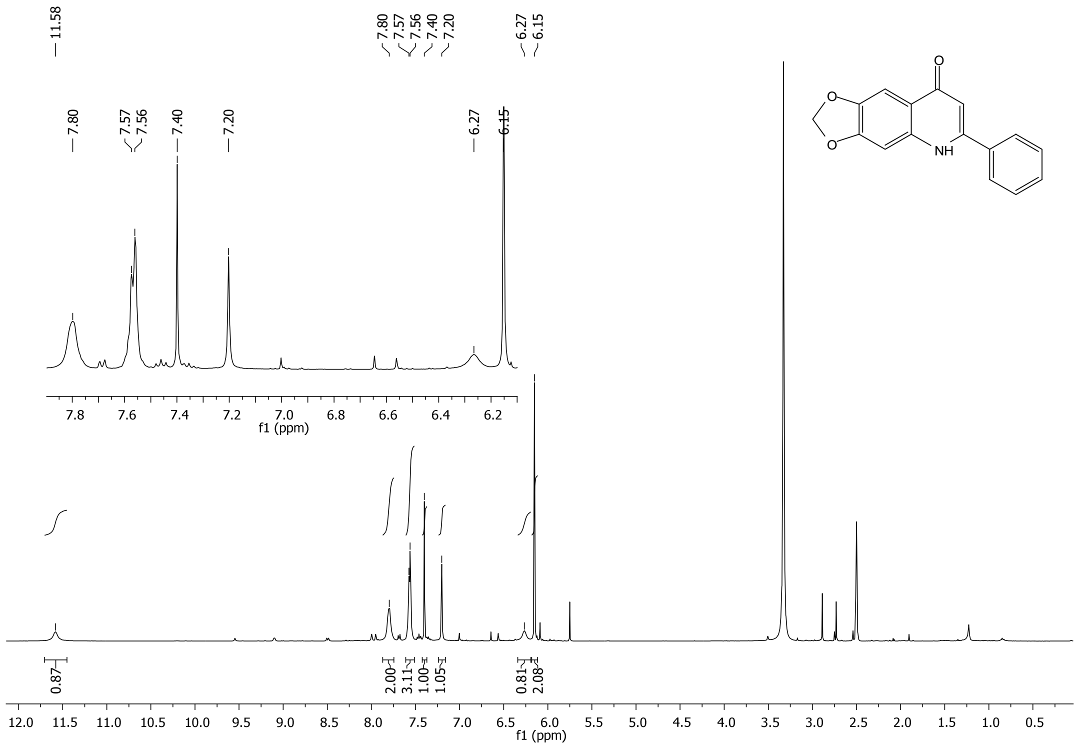

Figure S49. ^{1}H NMR of 6-phenyl-[1,3]dioxolo[4,5-g]quinolin-8(5H)-one (**2p**).

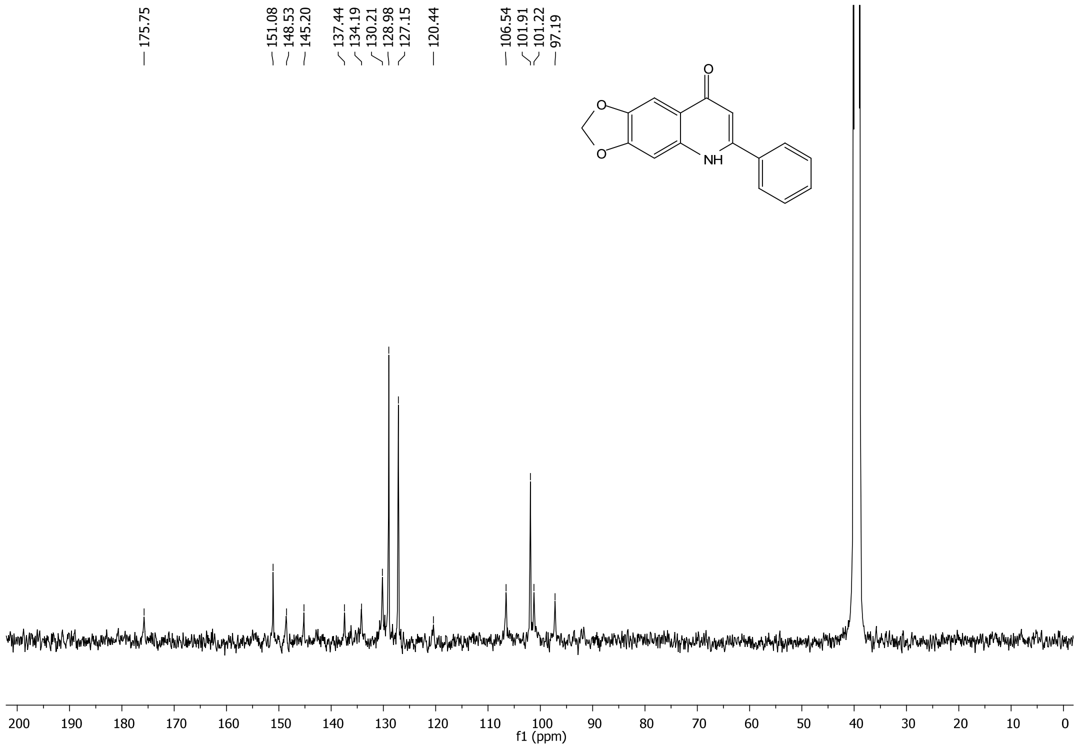

Figure S50. ^{13}C NMR of 6-phenyl-[1,3]dioxolo[4,5-g]quinolin-8(5H)-one (**2p**).

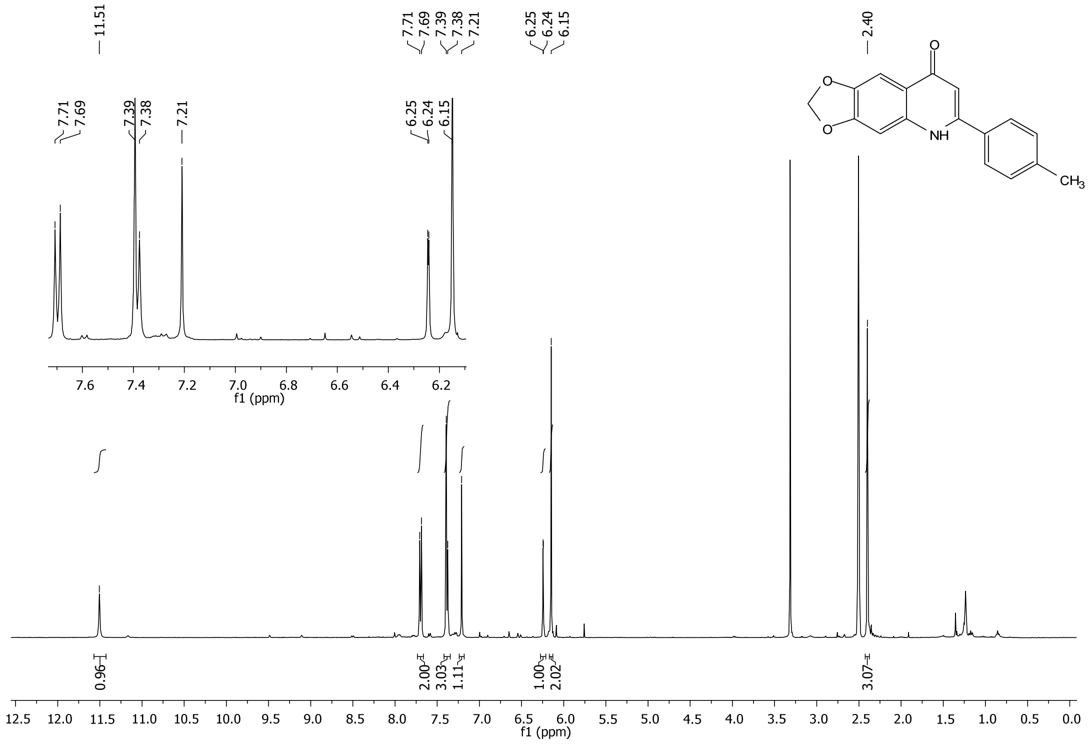

Figure S51. ^{1}H NMR of 6-(4-methylphenyl)-[1,3]dioxolo[4,5-*g*]quinolin-8(5*H*)-one (**2q**).

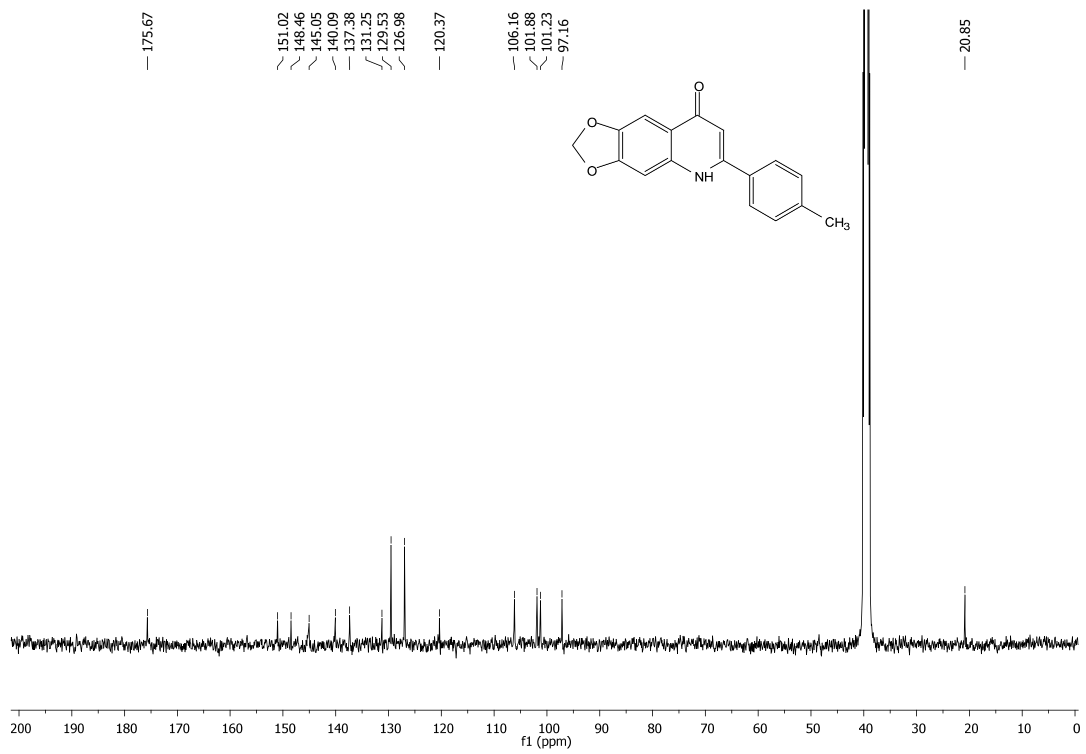

Figure S52. ^{13}C NMR of 6-(4-methylphenyl)-[1,3]dioxolo[4,5-*g*]quinolin-8(5*H*)-one (**2q**).

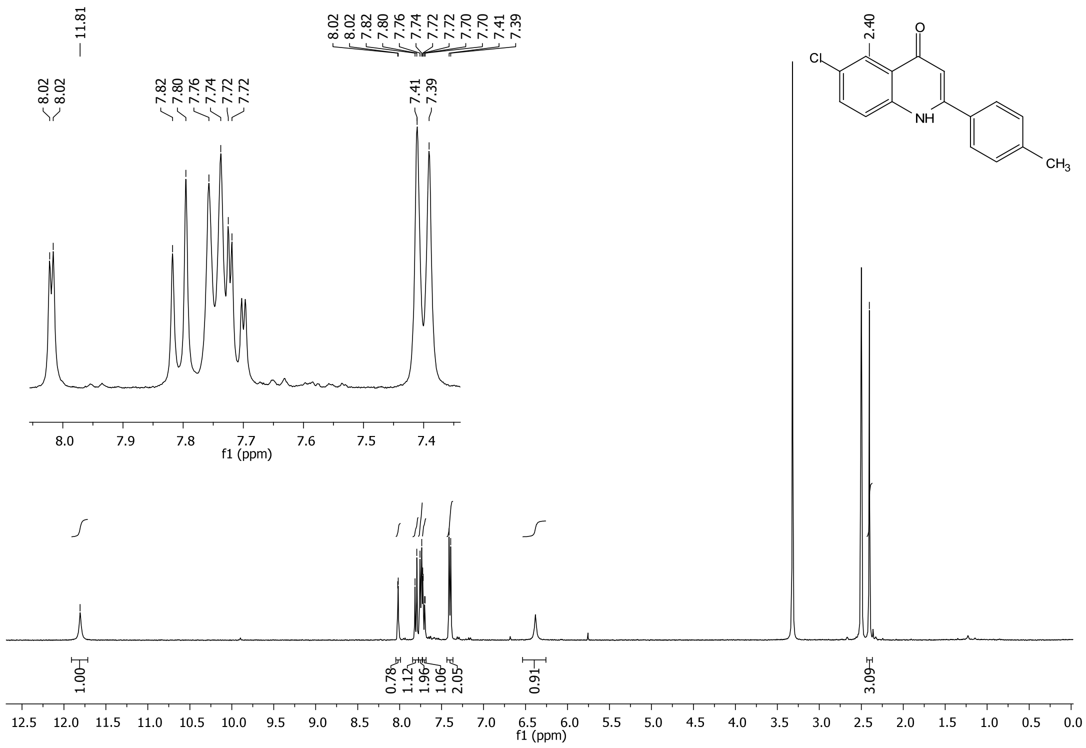

Figure S53. ^{1}H NMR of 6-chloro-2-(4-methylphenyl)quinolin-4(1*H*)-one (**2r**).

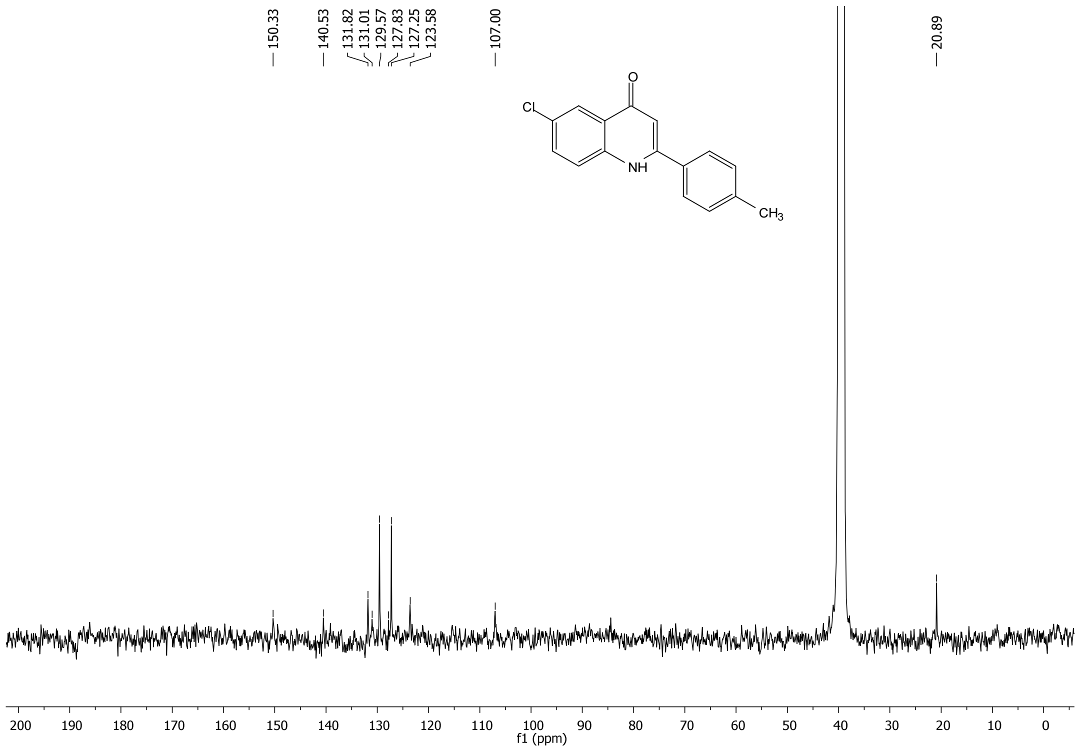

Figure S54. ^{13}C NMR of 6-chloro-2-(4-methylphenyl)quinolin-4(1*H*)-one (**2r**).

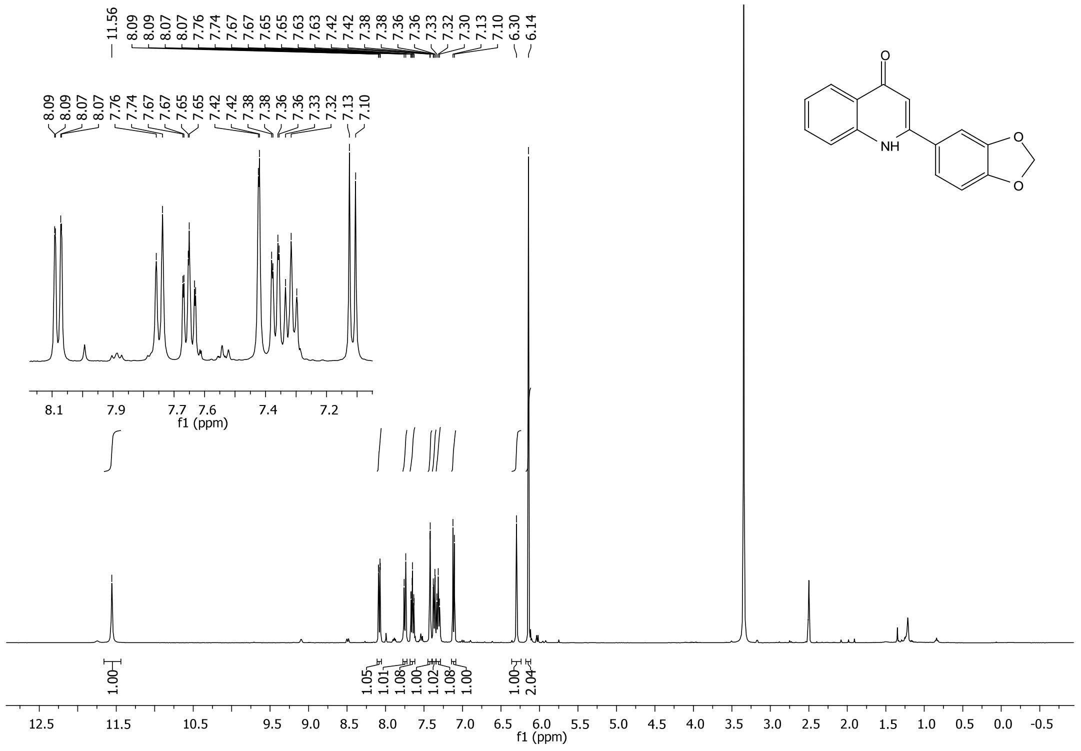

Figure S55. ^{1}H NMR of 2-(benzo[*d*][1,3]dioxol-5-yl)quinolin-4(1*H*)-one (**2s**).

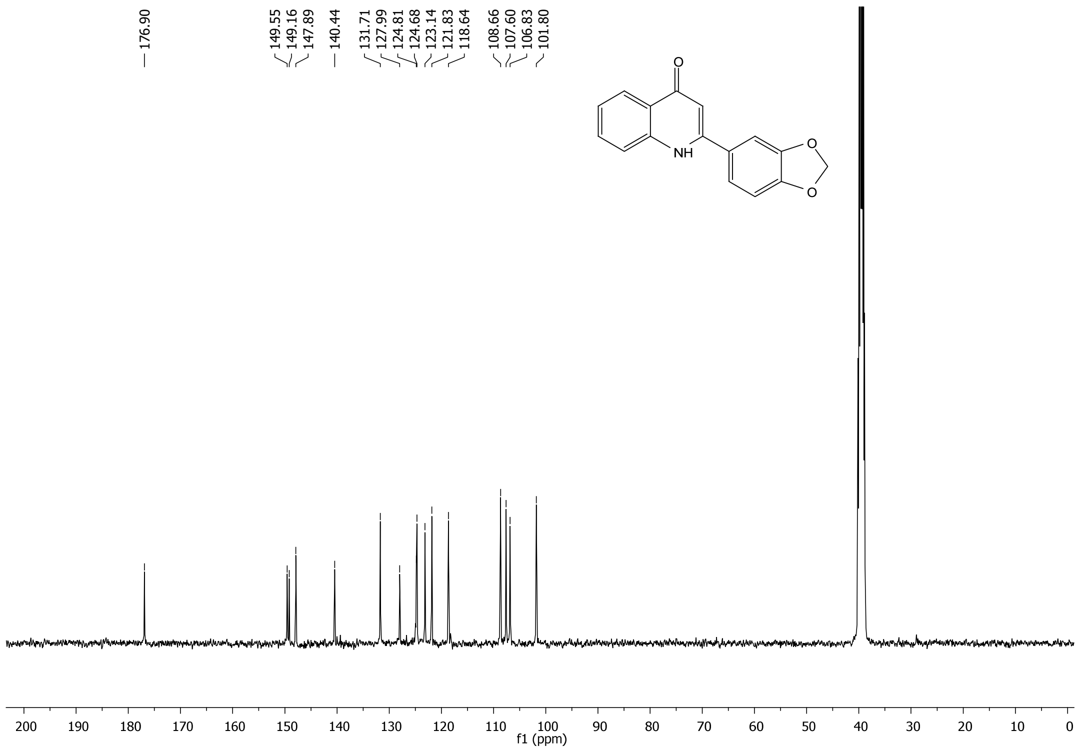

Figure S56. ^{13}C NMR of 2-(benzo[*d*][1,3]dioxol-5-yl)quinolin-4(1*H*)-one (**2s**).

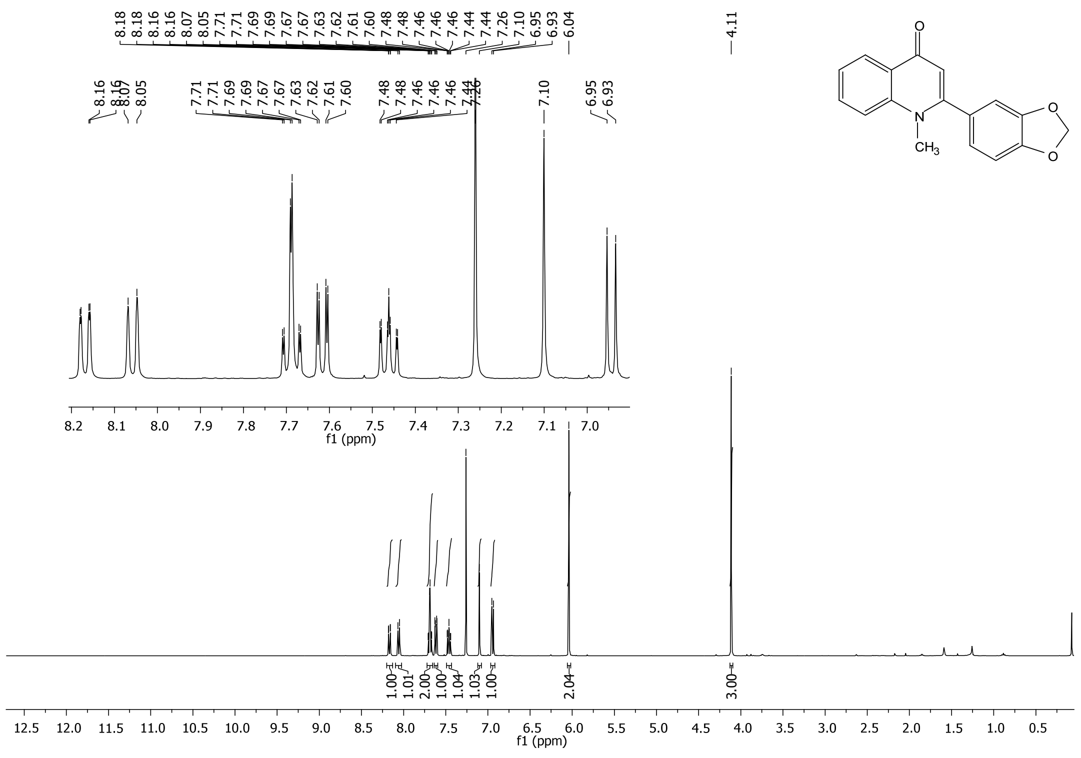

Figure S57. ^{1}H NMR of Graveoline.

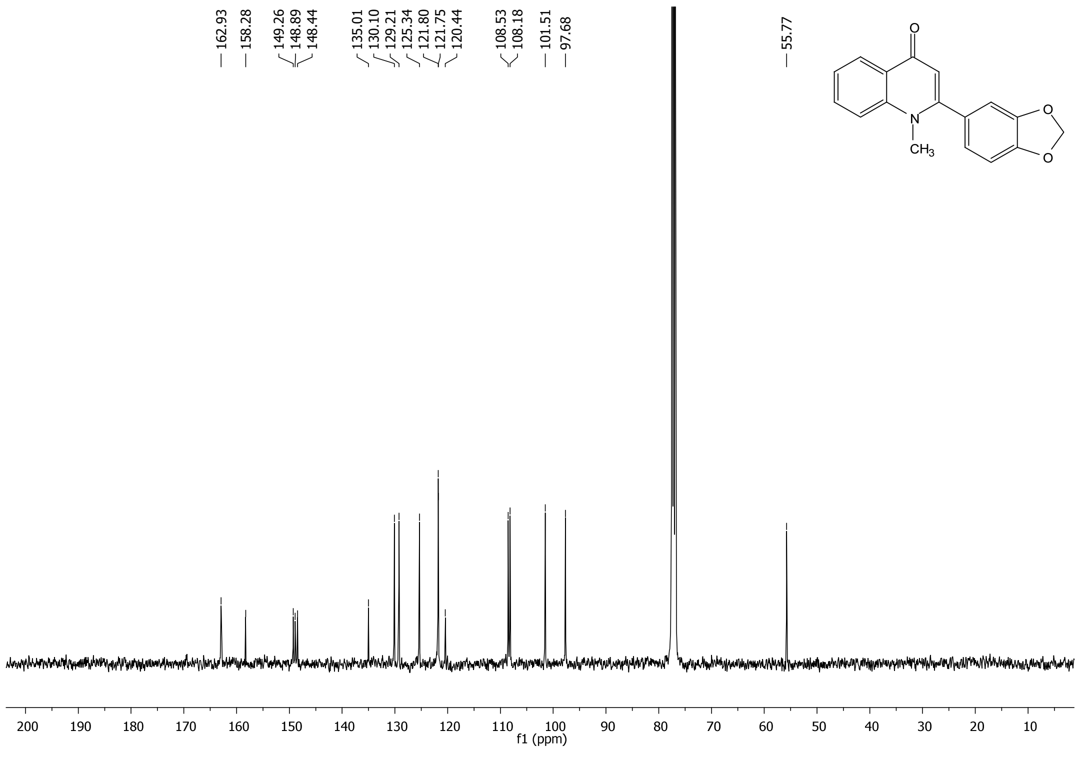

Figure S58. ^{13}C NMR of Graveoline.

3. References

1. Aksenov, N. A.; Aksenov, D. A.; Arutiunov, N. A.; Aksenova, D. S.; Aksenov, A. V.; Rubin, M., Unexpected cyclization of ortho-nitrochalcones into 2-alkylideneindolin-3-ones. *RSC Adv.* **2020,** *10*, 18440-18450.
2. Climent, M. J.; Corma, A.; Iborra, S.; Martí, L., Process Intensification with Bifunctional Heterogeneous Catalysts: Selective One-Pot Synthesis of 2′-Aminochalcones. *ACS Catal.* **2015,** *5*, 157-166.
